# Electrospun one-dimensional nanostructures: a new horizon for gas sensing materials

**DOI:** 10.3762/bjnano.9.202

**Published:** 2018-08-13

**Authors:** Muhammad Imran, Nunzio Motta, Mahnaz Shafiei

**Affiliations:** 1 Institute for Future Environments and School of Chemistry, Physics, and Mechanical Engineering, Queensland University of Technology (QUT), Brisbane, QLD 4001, Australia; 2 Faculty of Science, Engineering and Technology, Swinburne University of Technology, Hawthorn, VIC 3122, Australia

**Keywords:** 1D nanostructures, conductometric devices, electrospinning, gas sensors, optical sensors, resonators

## Abstract

Electrospun one-dimensional (1D) nanostructures are rapidly emerging as key enabling components in gas sensing due to their unique electrical, optical, magnetic, thermal, mechanical and chemical properties. 1D nanostructures have found applications in numerous areas, including healthcare, energy storage, biotechnology, environmental monitoring, and defence/security. Their enhanced specific surface area, superior mechanical properties, nanoporosity and improved surface characteristics (in particular, uniformity and stability) have made them important active materials for gas sensing applications. Such highly sensitive and selective elements can be embedded in sensor nodes for internet-of-things applications or in mobile systems for continuous monitoring of air pollutants and greenhouse gases as well as for monitoring the well-being and health in everyday life. Herein, we review recent developments of gas sensors based on electrospun 1D nanostructures in different sensing platforms, including optical, conductometric and acoustic resonators. After explaining the principle of electrospinning, we classify sensors based on the type of materials used as an active sensing layer, including polymers, metal oxide semiconductors, graphene, and their composites or their functionalized forms. The material properties of these electrospun fibers and their sensing performance toward different analytes are explained in detail and correlated to the benefits and limitations for every approach.

## Review

### 1 Introduction

The monitoring and control of air pollutants, toxic gases and explosives has become increasingly important for human wellness [1], security [2–3] as well as for the environment [4–11] in the last few decades. For instance, exposure to low concentrations of CO, CO_2_, NH_3_, NO_2_, CH_4_ and/or H_2_S, even in the range of a few parts per million (ppm), can cause suffocation, nervous system disorders, and/or asthma followed by death. Gas sensors are the primary devices used for the detection and monitoring of these pollutants. Employing nanotechnology in sensor applications has significantly improved the performance of such devices, providing enhanced sensitivity, selectivity, low power consumption and high integration flexibility. To date, many different gas sensing technologies have been developed. The predominant approaches to utilization are based on changes in the electrical conductance, optical properties, electrochemical potential or resonant frequency of the device [12–33].

Different types of nanostructures, including those based on metal oxides (MOx), organic and inorganic materials and carbon nanostructures, have shown promising sensing performance due to their unique characteristics, such as high surface-to-volume ratio, high surface active sites, high specific surface area and reactivity [12–13]. Among these nanostructured materials, one-dimensional (1D) materials are known to be highly suitable candidates due to their higher surface energy, increased number of reactive sites, effective charge carrier transport, and larger surface-to-volume ratio [34]. The large surface-area-to-volume ratio of nanofibers (NFs), hollow nanofibers (HNFs), nanotubes (NTs) and nanowires (NWs) with micro/mesoporous surfaces results in improved adsorption and better reaction kinetics of gas-sensitive materials.

**Figure 1:**
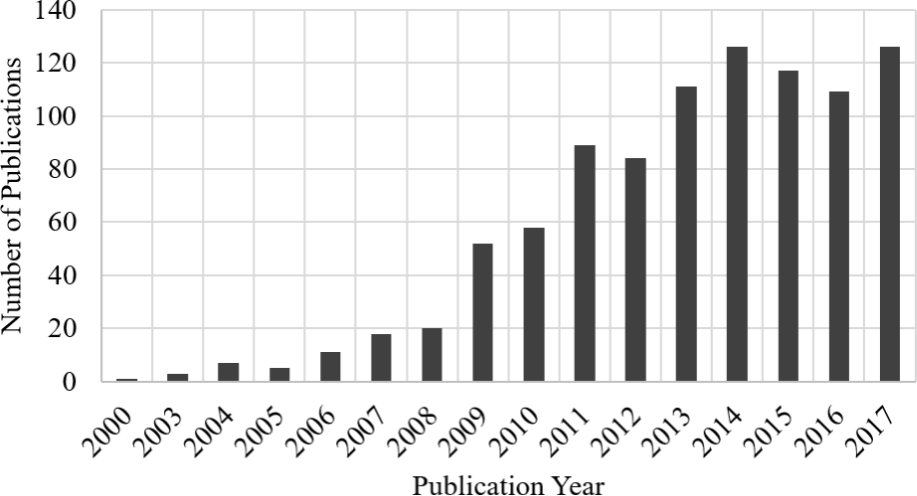
The annual number of patents and journal article publications on the topic of electrospun 1D nanomaterials used for gas sensing (Source: SciFinder^®^ searched on May 14, 2017).

Nanofibers can be produced by many different approaches. For example, by use of a molten-salt method, wet (or liquid) chemistry, nanocarving, self-catalyst growth, template-assisted (or sacrificial template) synthesis, chemical vapour deposition, thermal evaporation, spray pyrolysis or electrospinning [34–36]. Among these techniques, electrospinning is one of the most versatile and robust techniques for synthesis of functional nanofibers with unique structure and diverse properties [37–40]. The diameter of these functional fibers range between sub-micrometre to nanometre. The versatility of electrospinning has led to many publications in the field. The number of publications on electrospun fiber-based gas sensors has increased rapidly since the application of electrospinning to sample preparation. The number of patents and publications per year on electrospun 1D nanomaterials from 2000 until 2017 is shown in Figure 1.

A nanofiber film has a surface area approximately twice that of a continuous thin film. This property means that nanofibers are excellent candidates for gas sensing applications. Moreover, nanofibers derived from a variety of materials, such as polymers, metals, metal-oxides and composites, are fabricated in various assemblies (e.g., as mixed nanocomposites, double-layers, core–shell or hollow forms) using the electrospinning technique [37]. These electrospun nanofibers exhibit enhanced specific surface area, superior mechanical properties, nanoporosity and improved surface characteristics [37,40]. Such porous nanostructures provide a fundamental property that enhances the effective analyte adsorption and increases sensitivity. Therefore, the remarkable specific surface area and high porosity (≈70–90%) [41] due to the presence of small and large pores means that electrospun nanofibers are highly attractive as ultrasensitive sensors [42].

To date, many excellent review articles on the fabrication, alignment and application of electrospun nanofibers have been published [32,37,39–40]. However, to the best of our knowledge, a modern, comprehensive review on gas sensing applications of the electrospun 1D nanostructures (NFs, HNFs, NTs, and NWs) employing different types of materials integrated with different sensing principles does not exist. In 2009, Ding et al. [32] published a review article on gas sensors based on electrospun nanofibers, but since then, many reports on the development of the gas sensors employing electrospun nanofibers have been published. Recently, Choi et al. [43] reported a review on chemiresistive and optical sensors employing only semiconducting metal oxides and their functionalization by catalytic nanoparticles. Herein, we will comprehensively review and summarize the fabrication of electrospun 1D nanostructures based on diverse range of materials (including polymers, metal oxide semiconductors, graphene, and their composites or their functionalized forms) and their gas sensing performance in all available sensing architectures (including conductometric, acoustic resonators and optical). In addition, we provide concluding remarks and an outlook on this rapidly evolving research field on gas sensors based on electrospun 1D nanostructures.

### 2 Electrospinning

The electrostatic effect was first described in 1600 by Willian Gilbert [44–45] through a series of experiments using an electrically charged piece of amber. He noticed that a water droplet attained a conical profile in the proximity of charged amber and, if the charge was strong enough, tiny droplets would evolve from the larger water droplet. Gilbert’s experiments laid the foundation for electrospinning as well as electrospraying.

Electrospinning is a simple, robust and low-cost technique to generate polymer and composite fibers ranging from nanometres to a few micrometres in diameter [46–47]. In electrospinning, a high voltage source is used to produce fine fibers from a polymer solution or melt. A typical solution electrospinning setup comprises five major components: a metallic needle with a blunt tip, a syringe for containing the electrospun solution, a syringe pump to control the solution feeding rate, a direct current (DC) high voltage (HV) source, and a grounded conductive collector. Electrospinning is based on the electrostatic effect on a high viscosity fluid. In electrospinning, a hemispherical droplet of the polymer solution, suspended at the end of a capillary tube, is subjected to an electric force. Electric charges accumulate at the surface of the droplet and tend to elongate the droplet into a cone shape, where surface tension reduces the surface area by keeping it in a spherical shape. When the charged repulsive expansion exceeds surface tension based contraction, a charged jet of the solution is ejected from the tip of the Taylor cone and travels toward the target surface [48]. This charged jet exhibits chaotic motion as it travels toward the target. During the evolution of the electrospinning technique to modern times, Reneker and co-workers [49–50] have made a remarkable contribution by producing a diverse range of electrospun NFs of various morphologies, sizes and for various applications [51–55]. More recently, Deitzel et al. [56] and Shafiei et al. [57] developed a method to dampen the chaotic motion of the charged jet using electrostatic rings with a better control of the deposition area that is crucial for depositing 1D nanostructures on miniature sensing platforms (i.e., micro-electromechanical systems (MEMs)).

The electrospinning process is governed by various parameters such as viscosity, conductivity, molecular weight of fiber components, surface tension of polymer solution, electric potential, working distance, and flow rate. Each parameter significantly influences the morphology of the electrospun fibers, yet by their proper manipulation nanofibers with desired morphology and diameter are obtained. Bhardwaj et al. [58] and several other researchers summarized these parameters and their effect on fiber diameter and morphology for current electrospinning practices [58–62].

### 3 Nanostructure morphologies generated by electrospinning

Recently, a great deal of attention and research effort has been devoted to the development of electrospun fibers incorporated with functional nanoparticles (NPs) [39–40]. This practice significantly improves the performance of electrospun fibers/mats. In addition, the electrospun fibers/mats may reduce corrosion and/or oxidation in NPs, especially those with anisotropic structure [40]. Depending on the type of polymer and NPs, electrospun fibers incorporated with NPs are optimized for specific applications. This approach shows good potential for applications involving the self-assembly of anisotropic NPs to generate new functional devices [63].

There are two major methods for producing electrospun NP fibers [40]: (i) “indirect synthesis”, that requires some post-processing methods after electrospinning and (ii) “direct synthesis” of electrospun NP fibers during the electrospinning process. In the direct method, the composite fibers are electrospun from one single solution that contains the NPs. NP fibers formed in this way are not notably deformed or affected during the electrospinning process and their functionality remains viable in the final product [40]. To make the electrospinning of fibers containing NPs simple and easy, the NPs should be uniformly distributed within the solution. The properties of an electrospun hybrid fiber are tuned by controlling the density and distribution of the NPs in the fibers. Based on the type of NP (0D – dots, 1D – wires, 2D – plates), the nanofibers can be classified as:

- 0D NPs–electrospun fibers containing quantum dots or zero-dimensional particles,
- 1D NPs–electrospun fibers containing wires or similar elongated morphologies,
- 2D NPs–electrospun fibers containing plate-like or layered morphology particles and
- other organic or biological NPs–electrospun fibers [40].

**Figure 2:**
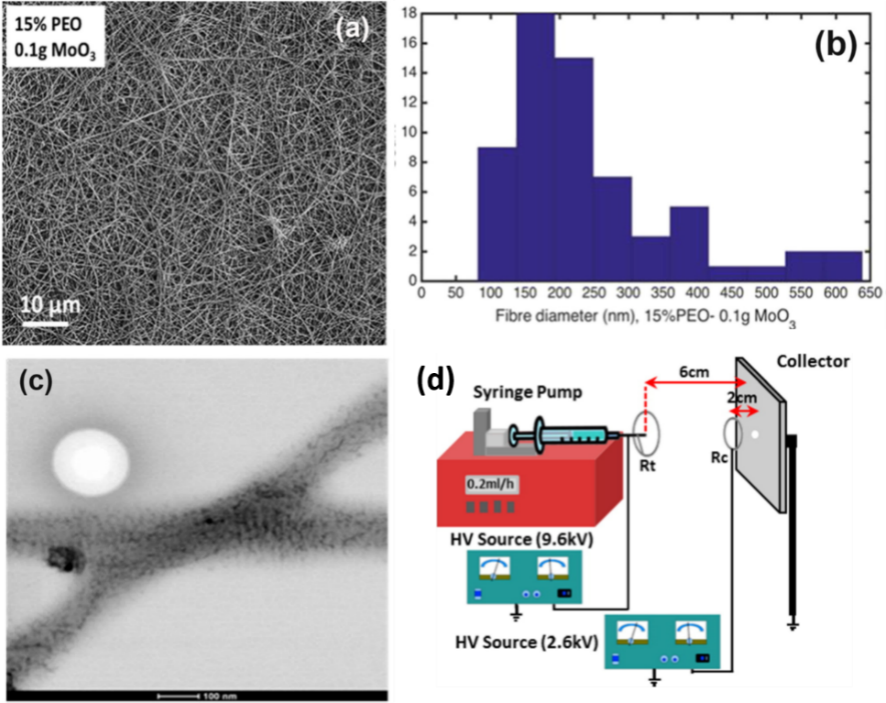
(a) SEM image; (b) size distribution of fiber diameters and (c) STEM image of electrospun nanofibers from a solution of 15% PEO with 0.1 g of MoO_3_ powder. (d) Schematic diagram of a multifield electrospinning setup. Reproduced with permission from [57], copyright 2017 Elsevier.

Recently, Shafiei et al. [57] reported electrospun ultrafine fibers with MoO_3_ nanoparticles embedded in poly(ethylene oxide) (PEO) polymer by using the direct method. The fibers are deposited directly onto a controlled and selective deposition area using a multifield electrospinning setup as shown in Figure 2. The scanning transmission electron microscopy (STEM) image confirms the uniform distribution of MoO_3_ NPs inside the electrospun fibers. Similarly, CuO NPs are embedded in polyurethane (PU) nanofibers using electrospinning [64]. These nanoparticles embedded in polymer nanofibers could be promising materials for room temperature gas sensing. Furthermore, graphene oxide (GO) sheets have also been incorporated with electrospun polyacrylonitrile (PAN) fibers [65–67]. The fibers reinforced with GO show better mechanical, electrical and thermal properties than the fibers without GO. These graphene-based composite electrospun fibers have been used for biosensor applications [65,68].

**Figure 3:**
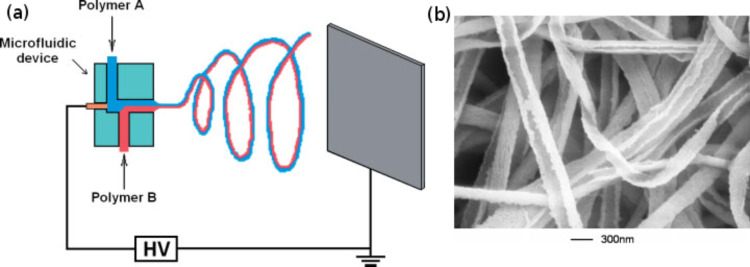
(a) Side-by-side electrospinning apparatus and (b) SEM image of poly(acrylonitrile)/polyurethane (PAN/PU) fibers. Reproduced with permission from [69], copyright 2005 Wiley.

Hybrid nanofibers with various morphologies, including mixed nanocomposite, dual-layer, core–shell and hollow nanofibers, are produced using different modified spinnerets [69–74]. Lin et al. [69] produced bi-component polymer fibers, showing the synergistic effects of the two different polymers, for the production of a new material using a microfluidic device as shown in Figure 3. These fibers are normally categorized by their cross-sectional structure such as: side-by-side, sheath–core and segmented-pie types. The nature of composite fibers produced in this way depends on their mutual interaction during electrospinning. Polymer solutions can either remain immiscible following a laminar flow or disperse to form a uniform homogeneous solution. The former gives rise to bi-component nanofibers, while the latter produces blended polymer fibers.

**Figure 4:**
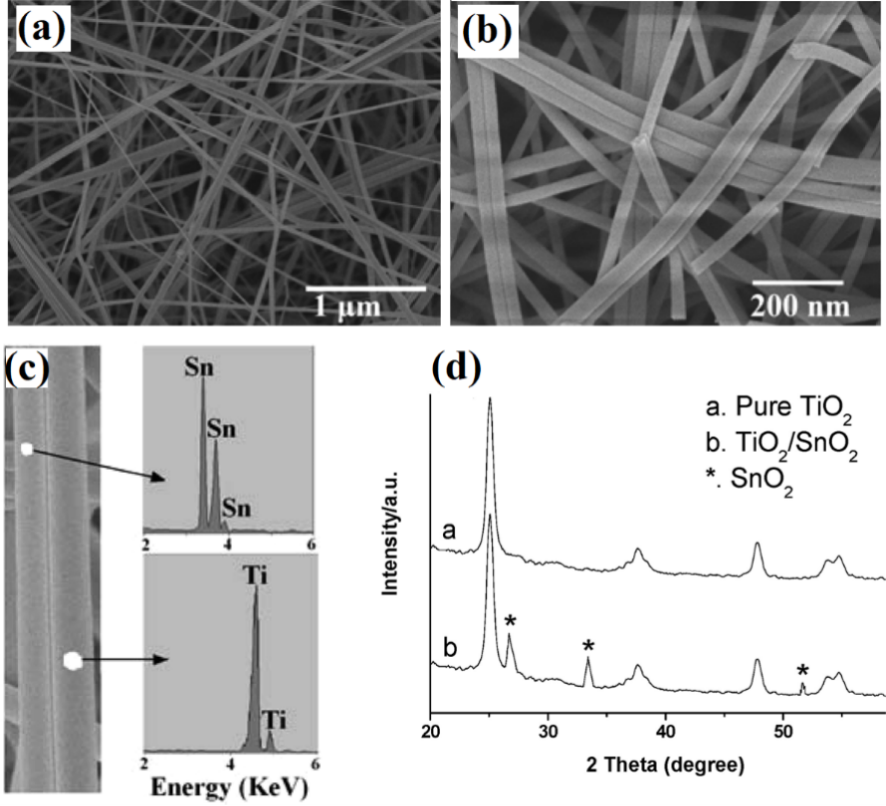
(a, b) Typical SEM images of the electrospun bi-component TiO_2_/SnO_2_ nanofibers; (c) EDS microanalysis of selected areas of the nanofiber, and (d) XRD diffraction patterns of the electrospun nanofibers. Reproduced with permission from [72], copyright 2007 ACS.

**Figure 5:**
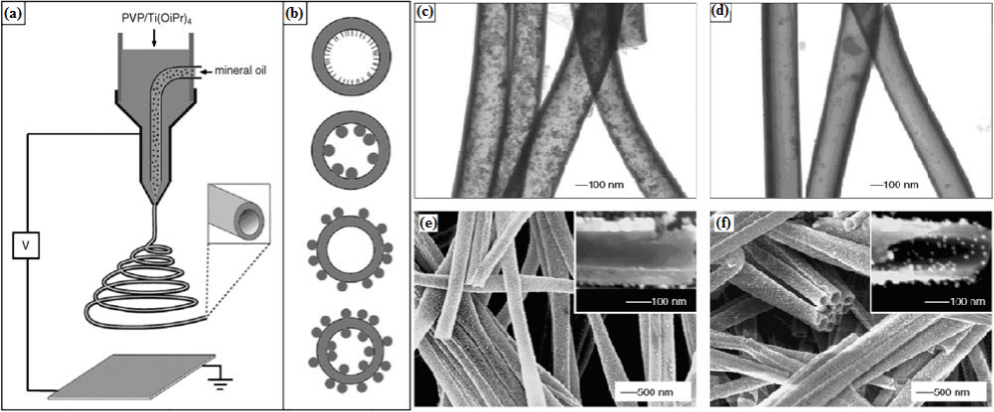
(a) Schematic illustration of the setup using a dual-capillary spinneret to directly electrospin hollow nanofibers with functionalized surfaces; (b) schematic drawings showing cross sections of hollow nanofibers whose surfaces are derivatized with functional molecules (the top plate) and nanoparticles (NPs) (the other plates). TEM images of hollow titania nanofibers immersed in an oil-based ferrofluid overnight. The hollow fibers are prepared by co-electrospinning, either with (c) or without (d) octadecyltrichlorosilane (OTS) added to the mineral oil. (e) SEM image of hollow fibers for which inner and outer surfaces are derivatized with CH_3_- and NH_2_-terminated silanes, respectively, and then immersed in citrate-stabilized Au colloids. Note that the Au colloids are selectively adsorbed onto the outer surfaces. (f) SEM image of hollow fibers with inner and outer surfaces treated with an NH_2_-terminated silane, followed by incubation with Au colloids. In this case, the Au colloids are adsorbed on both surfaces. Reproduced with permission from [73], copyright 2005 Wiley.

Dual-layer TiO_2_/SnO_2_ nanofibers have been reported by Liu et al. [72] using two syringes containing different solutions linked to a common spinneret. Each solution must have the same viscosity for uniform distribution in the final product. The resulting fibers from this technique are shown in Figure 4. Similarly, titania hollow fibers have been synthesized by Li et al. using a coaxial spinneret [73–74]. Titania hollow fibers can be produced by co-electrospinning a poly(vinyl pyrrolidone) (PVP) solution containing a titanium alkoxide (Ti(OiPr)_4_) and mineral oil. The mineral oil is removed at a later stage by calcination. In the same way, the interior of a hollow fiber is decorated by oil-dispersible nanoparticles using a silica capillary inside a stainless steel needle. The electrospinning setup and resulting hollow fibers are shown in Figure 5.

**Figure 6:**
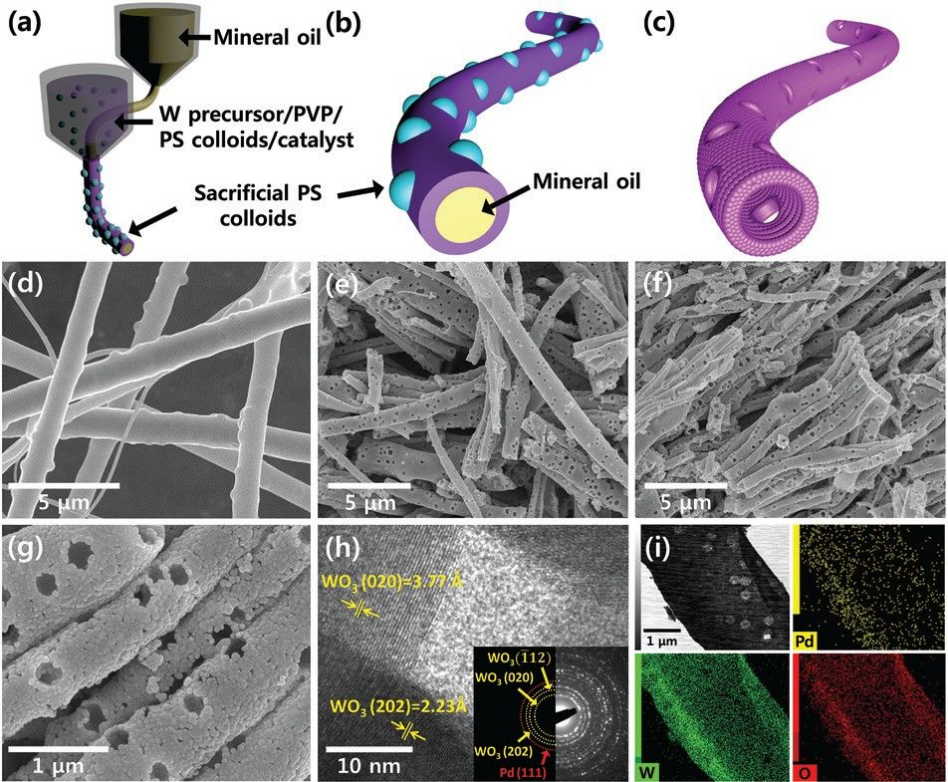
(a–c) Schematic illustrations of coaxial electrospinning using mineral oil in the core and composite solution in the shell. (d–i) SEM and TEM images of W precursor/PVP composite nanotubes decorated with PS colloid templates, and Pd-loaded macroporous WO_3_ NTs. Reproduced with permission from [75], copyright 2016 Royal Society of Chemistry.

Choi et al. [75] synthesized macroporous WO_3_ NTs using coaxial electrospinning combined with sacrificial templating. A porous structure using colloidal polystyrene (PS) particles in a solution with a W precursor and poly(vinyl pyrrolidone) was produced and a mineral oil was used to define the core. The PS particles and mineral oil are later removed by calcination. A schematic diagram of the electrospinning setup and resulting nanotubes are shown in Figure 6.

**Figure 7:**
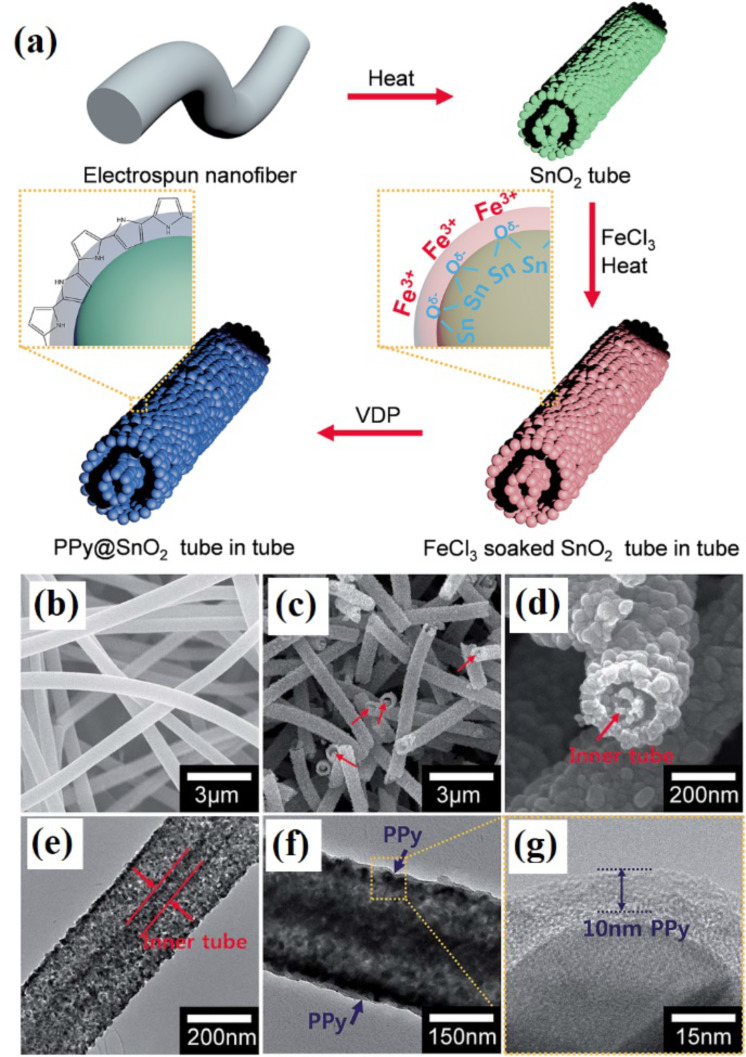
(a) Schematic diagram of the sequential fabrication of a PPy@SnO_2_ tube-in-tube structure. (b) FE-SEM image of the electrospun nanofibers. (c, d) Low- and high-resolution FE-SEM images of the tube-in-tube SnO_2_. (e) Low-resolution TEM images of the tube-in-tube SnO_2_. (f, g) Low- and high-resolution TEM images of the tube-in-tube PPy/SnO_2_. Reproduced with permission from [77], copyright 2017 Royal Society of Chemistry.

Fan et al. [76] developed a new fabrication strategy for synthesis of SnO_2_ NFs with a branch-on-stem morphology using electrospinning, oxygen plasma etching, sputtering and annealing. Electrospun PVP NFs were first etched with oxygen plasma to make a hierarchical template. Afterwards, a SnO_2_ film is deposited by sputtering and the PVP template is removed by annealing. The morphology of the NFs is dependent on sputtering time, resulting in uniformly distributed branches all over stem. Jun et al. [77] developed polypyrrole (PPy)-coated SnO_2_ tube-in-tube structures using single-nozzle electrospinning with a phase separation solvent method (Figure 7).

Liang et al. [78] reported a new method to synthesize electrospun hollow NFs using a two-step method. The In_2_O_3_ NFs are first produced by electrospinning and then are corroded by using 10% HNO_3_ to obtain hollow In_2_O_3_ NFs. The morphology of In_2_O_3_ nanostructures is also transformed from NFs to nanotubes (NTs) in a single capillary electrospinning process by changing the heating rate during the calcination process [79].

Similarly, In_2_O_3_ NFs are converted into nanoribbons (NRbs) by changing the experimental parameters [80]. The rapid evaporation of solvent and the concentration of the precursor are important parameters for the formation process of In_2_O_3_ NRbs. The In_2_O_3_ NFs have an average diameter of 180 nm, whereas the NRbs have an average width of 1 µm and thickness of 150 nm. Li et al. [80] found that just by increasing the ethanol concentration in the solvent mixture by keeping the precursor concentration constant, mixed NFs and NRbs are obtained. These mixed morphologies are further completely converted into nanoribbons by increasing the polymer concentration and salt content [80].

### 4 Gas sensors based on electrospun nanostructures

Gas sensors are devices specifically used to detect and discriminate between many different gases in the presence of other gases within low concentration ranges between a few parts per million (ppm) to parts per billion (ppb). The performance parameters of these gas sensors, including sensitivity, selectivity, response and recovery time, stability, reproducibility and reversibility, are strongly influenced by the properties of the sensing materials [12–14]. Chemical sensors are widely used for biomedical, healthcare, security and environmental applications. Table S1 in Supporting Information File 1 presents a summary of the different types of electrospun nanofibers reported to date that are used for gas sensors [4–11,32,81–89]. The following section discusses the details of these developed gas sensors based on the type of sensing platform and then by sub-categories of material(s) used to fabricate the electrospun nanofibers as the sensing layer. These materials include:

- metal oxide (MOx) semiconductors (e.g., SnO_2_, TiO_2_, SiO_2_) [83–84],
- doped MOx semiconductors [4–11],
- composite materials made of MOx semiconducting materials (e.g., ZnO-In_2_O_3_) [86],
- conducting polymer-based gas sensors (e.g., polypyrrole (PPy), polyaniline (PANI), polythiophene (PTh) and their derivatives) [32,87–88,90],
- MOx nanofibers surface functionalized by metal nanoparticles [75] and
- graphene sheets incorporated with MOx nanofibers [89].

#### 4.1 Conductometric gas sensors

Nanostructure-based conductometric sensors have found widespread commercial applications [91–92] due to their simplicity and enhanced gas sensing performance (high sensitivity, fast response/recovery and low operating temperature) and low cost. A typical conductometric gas sensor consists of an active sensing layer in which conductivity changes upon exposure to the target gas. The adsorption of gas molecules on the sensing layer leads to redox reactions by serving as an electron donor or acceptor which depends on the reductive or oxidative nature of the target gas compared to molecular oxygen. As the charge carrier concentration changes due to gas adsorption or desorption, the resistance of the sensing layer changes. To date, many types of nanomaterials in different structures have been synthesized and employed in conductometric devices for gas sensing applications [12,34,92].

**4.1.1 Pure semiconducting metal oxides:** Several types of electrospun metal oxide (MOx) semiconductors have been used for gas sensing applications. These semiconductors include titanium dioxide (TiO_2_) [93–95], tungsten trioxide (WO_3_) [27,96–110], copper oxide (CuO) [111], NiO [112], Co_3_O_4_ [113–114], iron oxide (Fe_2_O_3_) [115–116], tin dioxide (SnO_2_) [76,117–123], zinc oxide (ZnO) [124–130], and indium oxide (In_2_O_3_) [78,80,131–138]. Table S2 in Supporting Information File 1 summarizes the sensing performance of these electrospun pure MOx nanofibers.

Pure metal oxides have an intrinsic response towards a specific analyte gas that is remarkably dependent on grain size and specific surface area. A high response is expected for nanofibers with smaller grain size, smaller crystallite size, high porosity and larger surface area [130,139–140]. For example, the sensitivity of In_2_O_3_ nano/microtubes was improved by controlling the grain size via adjusting the calcination temperature [132]. The sensitivity increases as grain size is reduced with a corresponding increase in surface area. Grain sizes of 10, 15 and 23 nm were obtained for In_2_O_3_ NTs calcined at 400 °C, 600 °C and 800 °C. The In_2_O_3_ NTs sample calcined at 400 °C shows the highest performance for HCHO gas compared with its counterparts. The crystallite size can also be controlled by solution composition and polymer content followed by annealing and calcination [83]. A very low detection limit (9.7 ppb NO*_x_*) with an optimal response time (20 s) is achieved with nanocrystalline (5–10 nm) SnO_2_ NTs at room temperature [141]. Similar behaviour is exhibited by p-type NiO and CuO toward CO and NO_2_ [111–112]. The gas sensing behaviour also depends on the connectivity of the grains in highly crystalline nanofiber [113–114].

An opposite trend for crystal size is reported by Landau et al. [142] and Choi et al. [111] who obtained a higher response toward CO and NO_2_ with larger grain fibers. Moreover, the interparticle distance is also a key parameter in gas sensing properties. Less connected particles in Cr_2_O_3_ and Co_3_O_4_ nanofibers show a reduced gas sensing response [113]. Sensors calcined at 750 °C (grain size 36 ± 4 nm) showed 10–25% and 25–65% more sensitivity toward CO and NO_2_, respectively, than those calcined at 450 °C (grain size 17 ± 2 nm). The response of the NFs increases with an increase in surface area. TiO_2_ hollow fibers (HFs), of average diameter 200–2000 nm, exhibited higher response to CO at room temperature compared with solid fibers because gas molecules interact with the inner and outer diameters of the HFs. Moreover, the In_2_O_3_ and ZnO NTs with smaller diameter (≈50–100 nm) and thinner walls (≈10 nm) exhibited enhanced response compared with larger diameter NTs (≈500 nm) toward formaldehyde, CO and NO_2_ [126–128,133,143].

WO_3_ NTs with an average diameter of 200 nm showed a response of 45.2 toward 100 ppm of acetone at 250 °C compared with solid NFs with average diameter of 275 nm. These latter NFs give a response of 60.2 at 270 °C. The response time of WO_3_ NTs (5 s) is smaller than WO_3_ NFs (6–13 s) but the recovery time is longer (22 s) than WO_3_ NFs (4–9 s) because of different desorption rates in NTs compared with NFs [102,110]. A similar trend is shown by In_2_O_3_ NWs and NTs with similar response times but a longer recovery time for NTs compared with their counterparts [79,133]. Moreover, smaller diameter In_2_O_3_ NTs (≈100 nm) exhibited a higher response toward HCHO than larger diameter NTs (≈500 nm or 1 µm) [133].

The gas sensing response is also improved without tuning the microstructure of nanofibers just by the introduction of UV irradiation [105,144–145]. The response of TiO_2_ nanofibers is enhanced from 1.8/25 ppm to 18/25 ppm of hydrogen, whereas the response/recovery time reduced from 12.3/22.5 s to 2/6.9 s [145].

**4.1.2 Doped semiconducting metal oxide:** The intrinsic response of pure metal oxide 1D nanostructures can be tuned by changing the crystallite size, crystallinity and surface area. In general, these material parameters are modified by controlling the precursor concentration and/or calcination temperature [130]. The most effective approach to improve the gas sensing response of pure MOx 1D nanostructures is by functionalizing with different catalytic metals, MOx or noble metals [11,146]. Doping changes the reaction kinetics and electronic characteristics as well as the structural properties, including size and topology of the MOx 1D nanostructures, which leads to a change in their chemical sensing behaviour. [8,147–150]. Yamazoe et al.[151–152] explained the two types of functionalization mechanisms occurring in metal oxides as chemical sensitization and electronic sensitization. Traditionally, the doping is done by noble metals like Au [109,146,153–156], Ag [157–161], Pt [162–168], or Pd [75,107,148,169–176]. Moreover, noble metals can catalyse the gas sensing response of pure MOx NFs [86].

SnO_2_ is a widely-used metal oxide material for gas sensing applications because of its low cost and high chemical stability. However, wide application of SnO_2_-based gas sensors is limited by low sensitivity, slow response, lack of selectivity and the effects of aging. SnO_2_ NFs/NTs have been doped by alkaline earth (Ae) metals [86], lanthanides (Yb, Sr, Ce) [4,8,177], rare earth metals (Pr) [7], transition metals (Fe, Y, Ni) [5,178–179], copper [180], Pd [172] and Al [181]. In addition, NPs have been doped with Pt [153], Ag [157], Ca^2+^/Au [146,153–154] and LaOCl [182].

Doping with Ae metals exhibits an advantage in grain growth control [86]. For example, after thermal treatment, nanoparticle/nanograins show necked connections for each type of Ae-doped SnO_2_ NF. Therefore, a conduction channel can be established within each aggregate due to the space-charge layer region around each neck; this leads to fast capture and migration of electrons, and subsequently, enhanced gas sensing performance. The response of Sr/SnO_2_ NTs is 54.23% to 2000 ppm NH_3_, a value that is higher than other sensors due to the tubular structure. A lower detection limit of 10 ppm, faster response time of 6 s for 2000 ppm and 16 s for 10 ppm as well as improved reversibility was measured for Sr/SnO_2_ NTs toward NH_3_ gas at room temperature as compared with the pristine SnO_2_ NFs.

**Figure 8:**
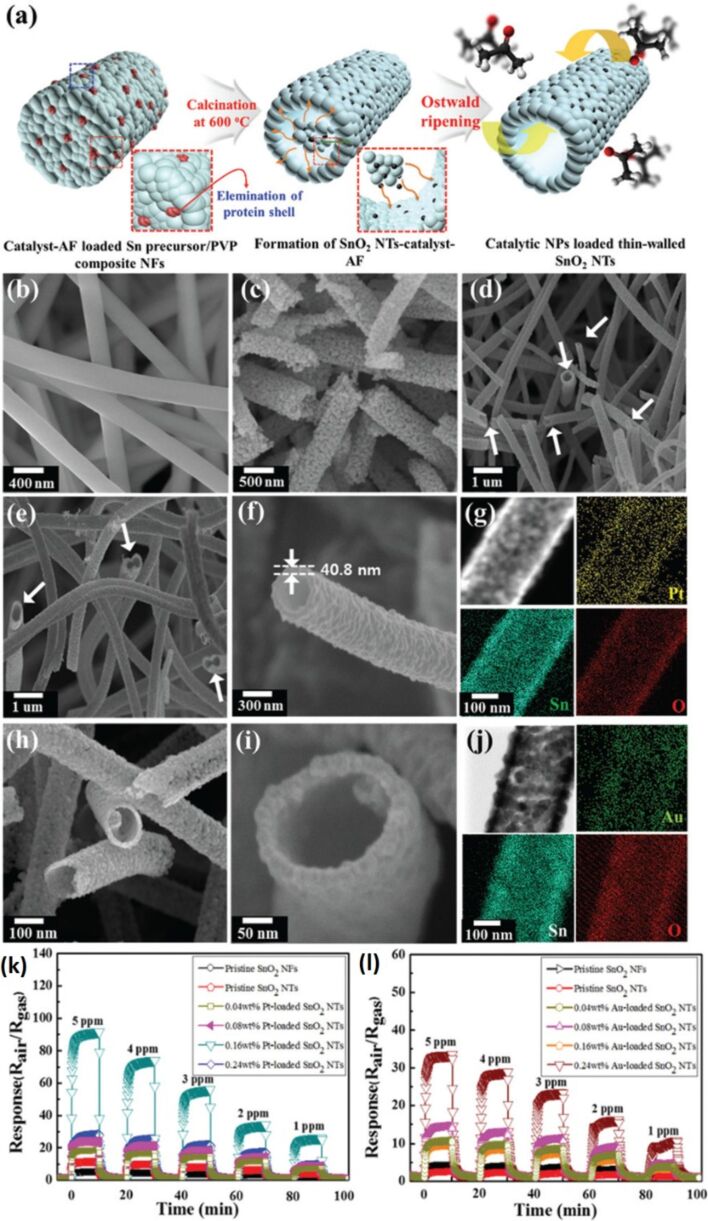
(a) Schematic illustration of the processing steps for SnO_2_ NTs functionalized by bio-inspired catalysts (e.g., Pt-loaded SnO_2_ NTs and Au-loaded SnO_2_ NTs). SEM image of: (b) as-spun Sn precursor/PVP composite NFs; (c) SnO_2_ NFs after calcination at 600 °C for 1 h at 4 °C min^−1^ heating rate; (d) thin-walled SnO_2_ NTs at 10 °C min^−1^ heating rate; (e) Pt-loaded SnO_2_ NTs; and (f) cross-sectional Pt-loaded SnO_2_ NTs. (g) EDX elemental mapping of Pt-loaded SnO_2_ NTs. SEM image of: (h) Au-loaded SnO_2_ NTs and (i) cross-sectional Au-loaded SnO_2_ NTs. (j) EDX elemental mapping of Au-loaded SnO_2_ NTs; (k) dynamic acetone sensing transition in a concentration range of 1–5 ppm at 350 °C and (l) dynamic H_2_S sensing transition in a concentration range of 1–5 ppm at 300 °C. Reproduced with permission from [183], copyright 2015 Royal Society of Chemistry.

Similarly, SnO_2_ NFs doped with Ca^2+^/Au exhibit a higher response (62) to 100 ppm of acetone at lower temperature (180 °C) with response and recovery times of 8 s and 5 s for 100 ppm of acetone [146]. SnO_2_ NTs have also been functionalized by bio-inspired Pt particles (2 nm diameter) at 0.16 wt % and Au particles (2.7 nm diameter) at 0.08 wt % using a protein nanocage by single-nozzle electrospinning [183] as shown schematically in Figure 8a. The diameter of the SnO_2_ NTs, as shown in SEM micrographs (Figure 8b–d), is 250–350 nm with a wall thickness of 40 nm. The elemental distribution for each NT is shown in Figure 8g,j. Pt-loaded SnO_2_ NTs exhibit a response of 92 toward 5 ppm of acetone compared to pure SnO_2_ NFs which respond 4.8 at 5 ppm acetone. SnO_2_ NTs show a response of 11 at 5 ppm acetone while Au-loaded SnO_2_ NTs show a higher response of 34 toward 5 ppm of hydrogen sulphide (H_2_S). In comparison, dense SnO_2_ NFs show a response of 2.6 at 5 ppm H_2_S and pristine SnO_2_ NTs show a response of 4.7 at 5 ppm H_2_S, respectively (Figure 8k,l) [183].

Similarly, a very low concentration of acetone (120 ppb) can be detected with a fast response/recovery time (11/6s) using thin-walled wrinkled layer SnO_2_ NTs functionalized with Pt by a controlled phase separation technique [163]. The selectivity of SnO_2_ NFs is also improved by doping with rare earth metals, e.g., Pr^2+^, Sr^2+^, and Y [5,7,177]. Many ethanol sensors have similar cross-sensitivity between ethanol and acetone. The sensitivity of a SnO_2_ gas sensor has been selectively increased from ≈10.8 to ≈18.9 toward 100 ppm of ethanol and the sensitivity toward 100 ppm acetone has been reduced from ≈8.9 to ≈3.9 by doping with Sr^2+^, resulting in a good discrimination between ethanol and acetone. The effect is obtained by inhibiting the growth of SnO_2_ grains resulting from substitution of Sn^2+^ with Sr^2+^ and enhanced surface area and reaction sites for analyte gas [177].

Al-doped SnO_2_ NTs exhibit a high response to low concentrations of formaldehyde by Sn^4+^ by Al^3+^ in a SnO_2_ lattice as well as increase in oxygen vacancies [184]. Pure and 8Al-Sn NTs (i.e., the Al/(Al + Sn) ratio is 8%) have nearly the same average diameter (120 nm inner diameter and 200 nm outer diameter) which suggests that Al doping has an insignificant effect on the morphology of SnO_2_ NTs. The optimum temperature for sensing response of Al-doped SnO_2_ NTs is 240 °C. The maximum response obtained from 8Al-Sn NTs toward 1000 ppb formaldehyde is as high as 7.82 at 240 °C. This response for 8Al-Sn is 4.1 times higher than that of pure SnO_2_ [184]. Al- and Co-doped SnO_2_ NFs (average diameter 80–120 nm) have also been evaluated for hydrogen sensing [181,185–186]. These Co-doped SnO_2_ NFs show a response of 24 toward 100 ppm of hydrogen at 330 °C with a response and recovery time of 2 s and less than 3 s, respectively [186].

Ni-doped SnO_2_ NFs are converted from solid to hollow NFs by tuning the heating rate. When the heating rate is as low as 2 °C/min, SnO_2_ NFs with solid cores are formed. Increasing the heating rate to 5 °C/min, a fraction of SnO_2_ NFs with hollow cores was formed. When the heating rate is as high as 10 °C/min, most of the SnO_2_ NFs are hollow. Ni-doped SnO_2_ NFs show diameters in the range 120 nm to 200 nm. The maximum response to acetone for these NFs is at 340 °C. By increasing the doping concentration of Ni in the range 0–10 atom %, response values are increased from 11.8 to 64.9 due to an increase in oxygen vacancies. The response and the recovery time are about 7 s and 30 s, respectively [179].

WO_3_ NFs/NTs functionalized by Pt [168,187], Pd [107,188], Cu [101], Ru [189], Rh_2_O_3_ [106], Au NPs [109], RuO_2_ NPs [189], La_2_O_3_ [104] as well as Pd-loaded ZnO nanocubes [1] have been extensively applied for sensing of acetone, ethanol, toluene, formaldehyde and volatile organic compounds (VOCs). WO_3_ NFs functionalized by Au NPs exhibit improved VOC sensing properties. Noble metals onto metal oxide NFs reduce the activation energy, thus increasing their efficiency [109]. The average diameter of as-spun fibers is 412 nm which, after annealing, reduces to 315 nm. The fiber diameter increases with increasing Au content. The average diameters of the WO_3_–Au-0.01M, and WO_3_–Au-0.1M composite NFs are 350 nm, and 370 nm, respectively. Au NPs act as nucleation sites (seed) on the surface that promote the WO_3_ crystal growth. The highest response toward 100 ppm of n-butanol is 7.3 for pure WO_3_ at 300 °C, 34.7 for WO_3_–Au-0.01 M and 152.7 for WO_3_–Au-0.1 M at 250 °C. WO_3_–Au-0.1M shows the highest voltage change when exposed to n-butanol [109].

Highly porous Pt- and Pd-doped WO_3_ NTs are synthesized using layer-by-layer (LBL) self-assembly of tungsten as well as catalyst precursor on PMMA electrospun nanofibers. Pristine WO_3_ NTs exhibit a high response (*R*_gas_/*R*_air_) of 63.59 to 5 ppm NO at 350 °C. On the other hand, a high response of 2.24 for the Pt-WO_3_ NTs and 2.35 for the Pd-WO_3_ NTs toward 5 ppm toluene at 400 °C is measured. A negligible NO response (1.25 for the Pt-WO_3_ NTs and 1.04 for the Pd-WO_3_ NTs at 5ppm) at 400 °C was found.

One problem associated with surface functionalization of NPs (3–50 nm) is their agglomeration. Traditional functionalization methods have limited ability to uniformly disperse NPs. A solution could be the encapsulation of NPs in polar proteins that could repel each other resulting in uniform dispersion over the entire surface area. Kim et al. [75,189–191] reported protein (apoferritin) encapsulated catalytic/noble metal NP functionalized MOx NFs with superior sensitivity and fast response in a high humidity environment (95% relative humidity (RH)). WO_3_ NFs functionalized by RuO_2_ [189] and Rh_2_O_3_ [106] NPs show improved response toward acetone at 350 °C. The fibers have an average diameter in the range 250–300 nm with no significant morphology change at different concentrations of Ru nanoparticles. The gas sensing response for different concentrations of acetone was in the range of 0.1–5 ppm at 350 °C. The highest response (78.61) was for Ru 0.090 wt %, that is 7.4% more than Rh_2_O_3_ (41.2) and the pristine WO_3_ (10.61). The fibers show maximum response at 350 °C. For selectivity tests, response to acetone is four times higher than the interfering gasses. The response and recovery time of 0.090 wt %-functionalized WO_3_ NFs is 7.9 s and 244 s, respectively [189].

**Figure 9:**
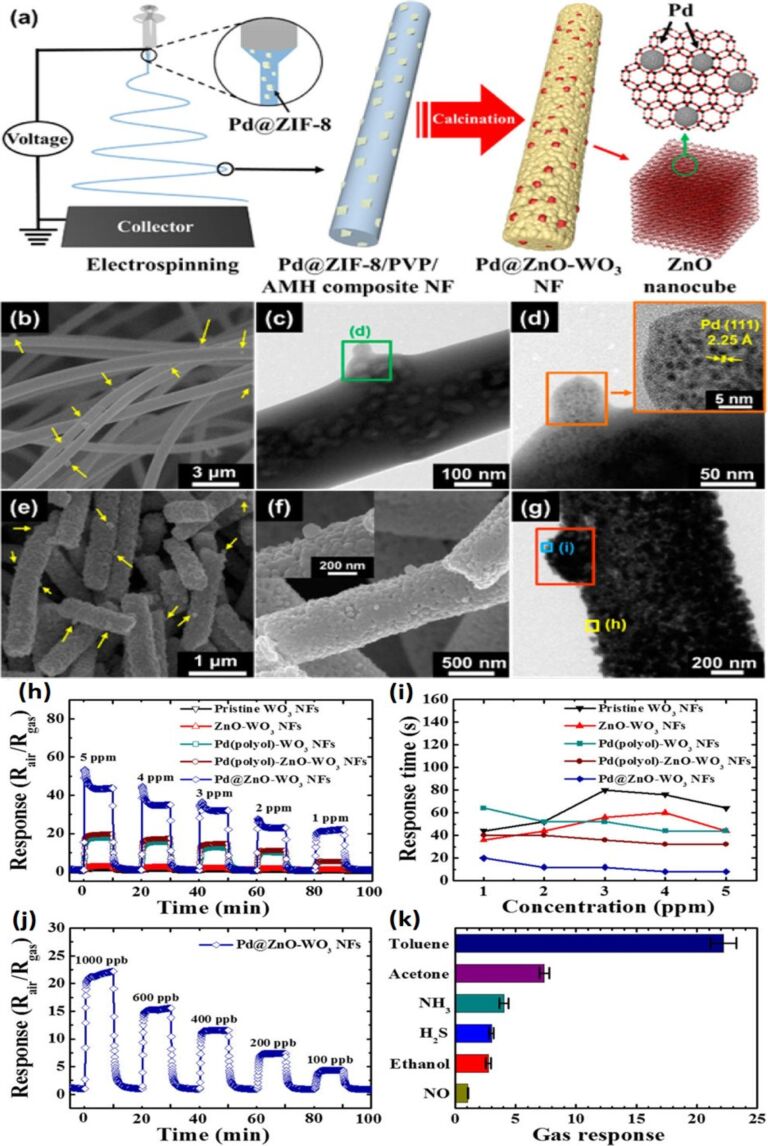
(a) Schematic illustration of synthetic process for the Pd@ZnO–WO_3_ NFs; (b) SEM image of as-spun ammonium metatungstate hydrate (AMH)/PVP/Pd@ZIF-8 NFs; (c,d) TEM images of AMH/PVP/Pd@ZIF-8 NFs and (inset) HRTEM image; (e,f) SEM images of Pd@ZnO–WO_3_ NFs and (inset) magnified image of surface; (g) TEM image of Pd@ZnO–WO_3_ NFs, response characteristics of pristine WO_3_, ZnO–WO_3_, Pd–WO_3_, Pd–ZnO WO_3_, and Pd@ZnO–WO_3_ NFs toward toluene in the concentration range of 1–5 ppm at 350 °C; (h) dynamic sensing transition; (i) response time evaluation; and (j) detection limit characteristics of the Pd@ZnO–WO_3_ NFs toward toluene down to 100 ppb at 350 °C; (k) selective toluene detection characteristics of the Pd@ZnO–WO_3_ NFs with respect to the multiple interfering analytes at a concentration of 1 ppm at 350 °C. Reproduced with permission from [1], copyright 2016 ACS.

WO_3_ NFs have also been functionalized by MOF-driven metal-embedded metal oxide catalysts and have been evaluated for toluene sensing [1]. WO_3_ NFs are functionalized by Pd-loaded ZnO nanocubes that result in multi-heterojunction Pd–ZnO and ZnO–WO_3_ interfaces. The as-spun Pd@ZnO-WO_3_ NFs have average diameter in the range 500–950 nm that reduces to 400–850 nm after calcination. A schematic shown in Figure 9a provides an interpretation of the SEM and TEM micrographs in Figure 9b–g. Figure 9c shows the Pd@ZIF-8 particles embedded in the WO_3_ NFs. The highest response is obtained at 350 °C using Pd@ZnO−WO_3_ NFs at a doping level of 0.136 wt % Pd@ZnO. Similarly, hollow SnO_2_ NTs have also been sensitized by MOFs. A Zn-based zeolite imidazole framework (Pd@ZIF-8, ≈80 nm) embedded with Pd NPs (≈2 nm) was used as a catalyst-loading platform for the efficient functionalization of a PdO@ZnO complex catalyst onto SnO_2_ NTs. Dual sensitized PdO@ZnO hollow SnO_2_ NTs (PdO@ZnO–SnO_2_ NTs) exhibited high response (*R*_air_/*R*_gas_ = 5.06) toward 1 ppm acetone at 400 °C with high selectivity, and fast response (20 s) and recovery (64 s) time under a highly humid atmosphere (95% RH) [192].

Gas sensing experiments were performed at an optimum temperature of 350 °C for detection of toluene in the concentration range of 1–5 ppm. The response of Pd@ZnO–WO_3_ NFs is (*R*_air_/*R*_gas_ = 22.22 to 1 ppm) as compared with pristine WO_3_ (*R*_air_/*R*_gas_ = 1.10), ZnO–WO_3_ NFs (*R*_air_/*R*_gas_ = 1.16), Pd(polyol)–WO_3_ NFs (*R*_air_/*R*_gas_ = 5.31), and Pd(polyol)–ZnO–WO_3_ NFs (*R*_air_/*R*_gas_ = 5.47) as shown in Figure 9h. The response time of Pd@ZnO–WO_3_ NFs is <20 s compared with pristine WO_3_ (44 s), ZnO–WO_3_ (36 s), Pd(polyol)–WO_3_ NFs (44 s), and Pd(polyol)–ZnO–WO_3_ NFs (32 s) (Figure 9i). The lowest concentration detected by Pd@ZnO–WO_3_ NFs is 100 ppb with a sensitivity of *R*_air_/*R*_gas_ = 4.37 at 350 °C (Figure 9j) [1,107].

ZnO is one of the most extensively used metal oxides for gas sensing applications [33]. ZnO-based 1D nanostructured materials have been doped by Ce [11,193], Pr [6], Er [194], La [190,195], Pt [190], Cu [190,196], Mn [197], Co [198], Al [199], Pd [176] and In [200] and have been used to detect acetone, acetic acid, ethanol, H_2_S, and CO.

ZnO hollow NFs functionalized by rare earth metals, such as Ce, show enhanced acetone sensing [193]. The Ce ion occurs either as Ce^4+^ and Ce^3+^ which is effective for improving the performance of chemical sensors. The surface morphology of Ce-doped ZnO HNFs is concave–convex and porous with an average diameter of 279 nm. This diameter is smaller than pure ZnO hollow NFs (316 nm); whereas the aperture at 240 nm is larger than pure ZnO hollow fibers (197 nm). The small diameter and larger aperture of the doped ZnO hollow fibers provides a higher specific surface area for gas interaction and significantly improves sensing performance. The highest response of Ce-doped ZnO HNFs (75.04/100 ppm) and (71.2/500 ppm) toward acetone is measured at an optimal operating temperature of 260 °C and 230 °C, respectively with a stability of over 40 days and retention of 96% of their initial performance. Furthermore, these sensors exhibit an excellent selectivity to acetone compared to other target gases, including ethanol, acetic acid, dimethylformamide (DMF) and ammonia [11,193].

Cho et al. [190] have synthesized Pt, Cu and La NPs (3–5 nm) to surface functionalize ZnO NFs using protein (apoferrtin (AF)) cage templates for enhanced acetone sensing. The as-spun ZnO NFs have an average diameter of 209 nm that decreases to 105 nm after calcination. The as-spun Pt, La, and Cu coated ZnO NFs have an average diameter of 100, 87 and 145 nm, respectively, after calcination. Pt and Cu NP-functionalized ZnO NFs exhibit approximately 6.4-fold (*R*_air_/*R*_gas_ = 13.07) and 3.0-fold (*R*_air_/*R*_gas_ = 6.04) enhanced acetone response compared with the response (*R*_air_/*R*_gas_ = 2.05) for pristine ZnO NFs at 450 °C. Whereas for La NP-functionalized ZnO NFs, a 9.3-fold improvement in nitrogen monoxide response (*R*_air_/*R*_gas_ = 10.06) is achieved compared with the response for pristine ZnO NFs. Functionalized ZnO NFs with 0.23 wt % Pt were shown to detect 29 ppb of acetone with a response of 2. The 0.23 wt % AF-Pt-NPs to ZnO NFs show the fastest response and recovery times of 12 s and 108 s, which are 8.3-fold and 2.3-fold faster than that (100 s, and 252 s) of pristine ZnO NFs to acetone at 5 ppm, respectively [190].

ZnO functionalized by rare earth metals (i.e., Er) exhibits improved ethanol sensing at an optimum temperature of 240 °C [194]. The diameter of the ZnO NFs decreased from 200 nm to 70 nm with an increase of Er content. The 0.5 atom %, 1.0 atom % and 2.0 atom % Er-doped ZnO NFs have an average diameter 165 ± 42 nm, 130 ± 35 nm and 70 ± 23 nm, respectively, compared with pure ZnO NFs which show average diameter of 200 ± 50 nm. The 1.0 wt % Er-doped ZnO NFs show the highest response of 37.3 toward 200 ppm of ethanol at 240 °C compared with that of pure ZnO NFs (10.1). The response/recovery time for pure ZnO NFs to 200 ppm ethanol is only 5/2 s, respectively, which is shorter than that of 1.0 atom % Er-doped ZnO NFs (12 and 3 s) [194]. Similarly, Al-doped ZnO NFs exhibit a response of 8.6 toward 100 ppm of ethanol at 250 °C with a response and recovery time of 5 s and 9 s, respectively [199]. Pd-doped ZnO NFs show a response of 5.5 toward 20 ppm of CO at 220 °C with response and recovery times in the range of 25–29 s and 12–17 s, respectively [176]. The 6 atom % Cu-doped ZnO NFs exhibit a high response of 18.7 to 10 ppm H_2_S compared with pure ZnO NFs with a response of 1.57 at 230 °C and response and recovery times of 18 s and 20 s, respectively [196].

Doping with multivalent ions (In^3+^, Al^3+^, Sn^4+^, etc.) may change the defect density and carrier concentration of a ZnO matrix [201–204]. A common type of ZnO doping is with indium, for which the product is called “IZO”. When the amount of indium is greater than 0.05, amorphous In_2_O_3_ forms and leads to a pronounced decrease in grain size. The optical band gap energy of IZO NTs also decreases with increased doping levels. Doped indium atoms may exist as trivalent cations that act as donor impurities at the substitutional sites of Zn^2+^, or may be present in amorphous In_2_O_3_. Both forms of In^3+^ may significantly influence gas sensing performance due to an increase in the number of free electrons. TEM images show the tubular structure of IZO with an average diameter in the range of 60–80 nm. The compactness of these NTs increases with an increase in indium content. The response values for IZO nanotube-based sensors with different indium contents indicate that the gas response decreases with higher indium doping levels. The response sharply increases with increasing ethanol concentration below 100 ppm. While for the ZnO sensor, the increased rate of response slows down above 1000 ppm ethanol. The ethanol responds to the IZO with 10% dopant in the nanotube sensor. However, the undoped ZnO exhibits lower response to ethanol by about 50% [200].

α-Fe_2_O_3_ nanostructured NFs/NTs have been functionalized by Ca [205], La [206], Pd [207], Sm [208], Al_2_O_3_ [209] and Ce [10] and applied to sensing of ethanol, acetone and formaldehyde. The sensing performance of α-Fe_2_O_3_ is improved by increased doping with Ca. Mismatch between the radii of Ca^2+^and Fe^3+^ ions is apparently responsible for grain refinement. For example, the grain size of α-Fe_2_O_3_ decreases from 28 to 7 nm with increase of Ca content in the range of 1–15 mol % compared with that of pure α-Fe_2_O_3_ (31 nm). Sensors with 7 mol % Ca doping show the highest response to ethanol (26.8/100 ppm) and acetone (24.9/100 ppm) at 200 °C compared with pure α-Fe_2_O_3_ with response of 5.26 to 100 ppm ethanol at 250 °C. However, the sensor shows cross sensitivity to ethanol and acetone. In comparison to Ca-doped α-Fe_2_O_3_, La-doped α-Fe_2_O_3_ NTs show a similar response (26/100 ppm) at 240 °C toward acetone with a much shorter recovery time of 10 s [206]. However, Nd-doped α-Fe_2_O_3_ NTs exhibit almost double the response (44) of La-doped α-Fe_2_O_3_ NTs at 240 °C toward 50 ppm of acetone with the response and recovery times of 19 s and 50 s, respectively. These response parameters are significantly different to that of pure porous α-Fe_2_O_3_ NTs (2.6). Nd-doped porous α-Fe_2_O_3_ nanotube sensors can detect 500 ppb of acetone with a response of 2.4 [207].

In_2_O_3_ 1D nanostructures functionalized by Co [210], Nd [211], Eu [212], Yb [213–214], Pd [148], Mg [215], Ag [161], Er [216], V [217] and Sm [218] have shown promising results for gas sensing applications. For example, In_2_O_3_ NWs functionalized by Co exhibited a response of 16.5 to 100 ppm of ethanol at 300 °C with very short response and recovery times (2 s and 3 s), respectively [210]. However, Pd-doped In_2_O_3_ NFs show a response of 26 toward 100 ppm of ethanol at lower temperature (240 °C) with shorter response and recovery times (1 s and 10 s), respectively. Rare earth metals and their oxides significantly improve gas sensing properties of semiconducting metal oxide materials by substitution in their lattice structure. For example, 3 wt % Eu_2_O_3_-doped In_2_O_3_ NTs show excellent discrimination between acetone and ethanol with a response of 44 toward 50 ppm ethanol as compared with acetone (11) at 260 °C with response and recovery time of 3 s and 21 s, respectively [219]. Similarly, Yb-doped In_2_O_3_ NTs (average diameter 200 nm) fabricated by single capillary electrospinning are used for formaldehyde sensing [213]. Yb-doped In_2_O_3_ NTs exhibit a response 3.8-fold higher (69.8) than pure In_2_O_3_ NTs (18.4) for 100 ppm of formaldehyde at 230 °C. The response and recovery times of Yb-doped In_2_O_3_ NTs to 100 ppm formaldehyde are about 4 s and 84 s, respectively. Yb-doped In_2_O_3_ NTs show a response of 2.4 toward 100 ppb of formaldehyde [213].

**Figure 10:**
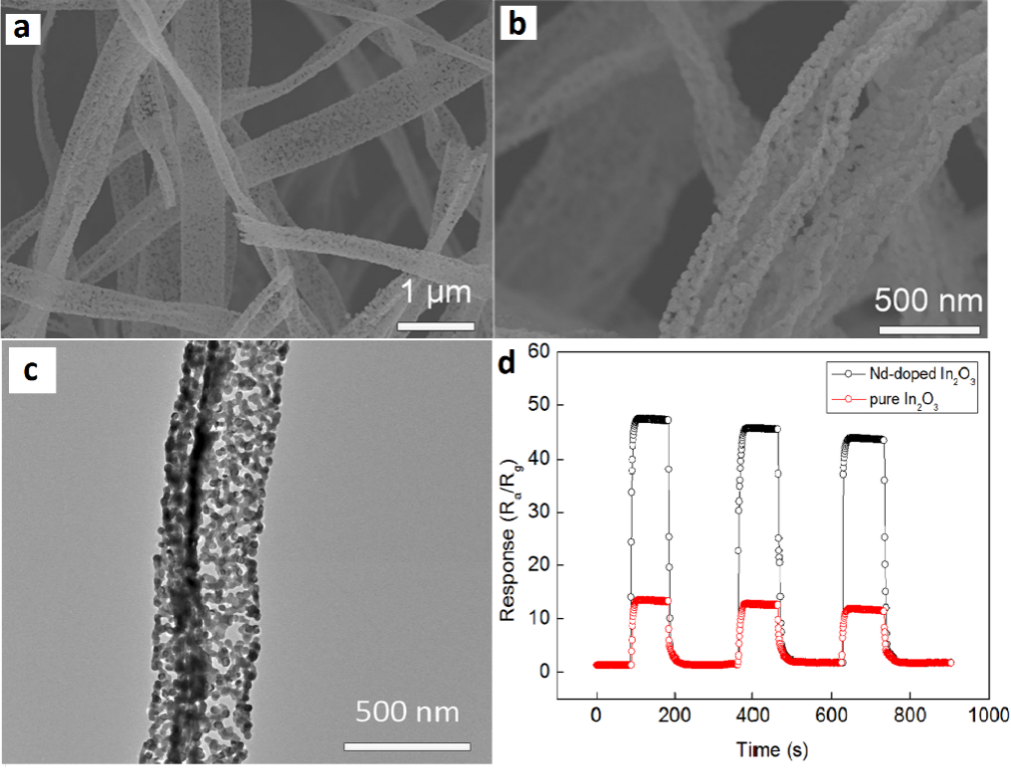
SEM images of (a) pure In_2_O_3_ porous NTs; (b) Nd-doped In_2_O_3_; (c) TEM image of Nd-doped In_2_O_3_ porous NTs; (d) response and recovery curves of pure and Nd-doped In_2_O_3_ porous nanotube sensors to 100 ppm formaldehyde at 240 °C. Reproduced with permission from [211], copyright 2016 Springer.

Mg-doped In_2_O_3_ NTs exhibit a high response at low temperature (150 °C). Pure and Mg-doped In_2_O_3_ NTs have an average diameter of 80 nm. The smooth surface of In_2_O_3_ NTs becomes coarser with doping of Mg. Mg doping introduces protrusions (mean size ≈29 nm) on the outer surface of Mg-In_2_O_3_ NTs. Mg doping leads to growth of some In_2_O_3_ grains on the outer walls of NTs to form protrusions. The response of Mg-doped In_2_O_3_ reduces with an increase in temperature and becomes stable after 300 °C. The maximum response of 173.14 toward 10 ppm of H_2_S is obtained at a much lower temperature of 150 °C. In comparison, pure In_2_O_3_ NTs exhibit a response of 12.31 at the same temperature. Mg-doped In_2_O_3_ NTs are shown to detect 0.5 ppm of H_2_S.The enhanced response is due to the substitution of Mg^2+^ ions as acceptors in the In^3+^ lattice resulting in high oxygen vacancies [215]. Similarly, Nd-doped In_2_O_3_ NTs showed enhanced formaldehyde sensing due to their porous and cracked morphology. Nd-doped In_2_O_3_ NTs have an average diameter of 200 nm as shown in Figure 10a–c. The optimum doping amount of Nd was 11 mol %. Nd-doped In_2_O_3_ porous NTs showed a high response of 46.8 to 100 ppm of formaldehyde at optimum temperature (240 °C) as shown in Figure 10d, whereas the response and recovery times are 8 s and 22 s, respectively. The detection limit of the Nd-doped In_2_O_3_ NTs was 100 ppb with a response of 2.4 [211].

W-doped NiO NTs (average outer diameter of 90 nm) have been applied to xylene gas sensing [220]. W-doped NTs with the molar ratio W^6+^: Ni^2+^ of 2:100, show outstanding sensing properties toward xylene with enhanced response, selectivity and repeatability characteristics. Mole number ratios of W^6+^ to Ni^2+^ at 0, 1, 2 and 4 mol % can be synthesized by electrospinning. With increased doping content, the grain size of W-doped NiO reduces, and the NTs become less porous. The 2 mol % W-doped NiO NTs show the highest response of 8.74 toward 200 ppm xylene at 375 °C, which is about 3.3-times higher than sensors based on undoped NTs. The response and recovery time of 2 mol % W-doped NiO NTs is 178 s and 152 s, respectively. Thus, W-doped NiO NTs can successfully detect low concentrations (15 ppm) of xylene with a response of 2.17 [220].

Pd-doped TiO_2_ NFs (average diameter 250 nm) show high sensitivity to NO_2_ at a relatively low temperature of 180 °C. The average diameter of pure TiO_2_ and Pd-doped TiO_2_ NFs is 450 and 250 nm after calcination at 600 °C. The rough surfaces of pure TiO_2_ NFs convert into smooth surfaces with Pd doping. Pd-doped TiO_2_ NFs are densely packed with nanocrystals of diameter 20–30 nm. The Pd-doped TiO_2_ and pure TiO_2_ NF-based sensors show the highest responses of 38 and 13 at temperatures of 180 °C and 200 °C, respectively. Moreover, the Pd-doped TiO_2_ NF sensor exhibits five times higher response than that of the TiO_2_ NF sensor at 0.8 ppm, even at an operating temperature that is 20 °C lower [175].

Pure and 0.08 wt % Pt-doped SnO_2_ NFs have been used for H_2_S gas sensing using a micro-machined (MEMS) platform [165]. The average diameter of pure SnO_2_ and 0.08 wt % Pt–SnO_2_ NFs are in the range of 200–300 nm and ≈120 nm, respectively. The decrease in fiber diameter is because of retardation of grain growth of SnO_2_ particles due to the presence of secondary Pt nanoparticles. The 0.08 wt % Pt-doped SnO_2_ NF sensors exhibit a response of 23–121 toward 4–20 ppm H_2_S at 300 °C, which is 25.9–40.6-fold higher than the response of pure SnO_2_ NFs. The introduction of the additive may increase the density of semiconductor surface adsorption sites, enhancing oxygen adsorption at the grain surface, leading to improvement in the sensing response. The Pt catalyst also may cause an increase in the response. As the temperature increases up to 500 °C, the response decreases. A response time of 1 s for a 0.08 wt % Pt-doped SnO_2_ NF sensor is significantly less than that for an undoped SnO_2_ NF sensor (2–7 s). However, all these sensors show longer recovery times. Even at 400 °C, the recovery values of an undoped SnO_2_ NFs sensor ranges from 267 to 281 s. These recovery values are much longer than the corresponding response time values (2–7 s). The 0.08 wt % Pt-doped SnO_2_ NF sensor shows recovery time values under similar conditions that range from 214 s to 267 s [165]. A detailed analysis of MOx sensors doped with different metals is shown in Table S3 in Supporting Information File 1.

**4.1.3 Composite semiconducting metal oxides:** The performance of semiconducting metal oxide gas sensors is improved by mixing two or more metal oxides to make composites. In many cases, these composites have advantageous properties of both metal oxides. Moreover, the porosity of the nanofibers can also be increased by mixing two or more metal oxides together having a mismatched crystal (lattice) size, resulting in enhanced gas diffusion (penetration) and subsequently fast response/recovery time. Furthermore, a hybrid structure (i.e. p–n junction, n–n junction or p–p junction) facilitates low operating temperature gas sensing with high sensitivity by a high proportion of oxygen vacancies and efficient electron transfer [221]. For example, ZnO–SnO_2_ composite HFs exhibited excellent response (83) to 20 ppm of ethanol at 260 °C with a response time of 4–7 s and recovery time of 4–5 s [222]. One of the problems with ethanol gas sensors is their similar sensitivity to acetone. ZnO–SnO_2_ hollow NF based sensors show excellent selectivity to ethanol as compared with acetone, ammonia, glacial acetic acid, DMF, and formaldehyde [222]. A ZnO shell grown on SnO_2_ NFs by a hydrothermal method exhibits a response of 392.29 toward 100 ppm of ethanol at 200 °C with response/recovery times of 75 s/12 s [223]. Importantly, the operating temperature of ZnO–SnO_2_ composite NTs decreases from 215 °C to 140 °C by decorating with Ag NPs using a seed-mediated growth method [158]. CuO/SnO_2_ mixed NFs synthesized by a double needle electrospinning technique have been used for H_2_S sensing. In this method, CuO NFs with an average diameter of 110 nm consist of larger nanograins than SnO_2_ nanofibers with an average grain size of 20 nm. CuO/SnO_2_ mixed NFs exhibit a very high response of 522 toward 10 ppm of H_2_S at 300 °C compared with pure SnO_2_ NFs (19). These CuO/SnO_2_ mixed NFs show response time/recovery times of 1/305 s [224].

In_2_O_3_-CeO_2_ NTs synthesized by electrospinning exhibit an excellent response toward H_2_S at low temperature (25–110 °C) and to acetone at relatively high temperature (300 °C) [225]. The outer diameter and wall thickness are tuned in the range of 90–180 nm and 15–9 nm, respectively, by changing the molar concentration of In_2_O_3_ and CeO_2_. The diameters and wall thickness of In_75_Ce_25_, In_50_Ce_50_, In_25_Ce_75_ and CeO_2_ NTs are ≈100, 120, 140, 180 nm and 12, 10, 9, and 9 nm, respectively. The diameter increases with an increase in Ce concentration in different samples whereas the wall thickness decreases [225]. The binary In_2_O_3_-CeO_2_ (In_75_Ce_25_) NTs exhibit a high response of 498 to 20 ppm of H_2_S at 80 °C and 30 toward 200 ppm of acetone at 300 °C, respectively. The response and recovery times for H_2_S and acetone are 64/204 s and 9/80 s, respectively [225].

Al_2_O_3_–In_2_O_3_ composites with a heterostructure and mesoporous tubular structure have been applied to room temperature NO*_x_* sensing [226]. These composite NTs can detect 291 ppb of NO*_x_* at room temperature with a response of 0.74 and a response time of 24 s. The atomic ratios of In and Al were 100:0, 100:15, 100:20 and 100:25, and are labelled as pure In_2_O_3_ NTs, meso-15AI NTs, meso-20AI NTs and meso-25AI NRs, respectively. The average diameter of the NTs is around 200 nm. The crystallite size of In_2_O_3_ reduces with increasing Al_2_O_3_ content. The grain size in pure In_2_O_3_ reduces from 30–50 nm to 8–13 nm in Al_2_O_3_–In_2_O_3_ composite NTs. The NTs containing 20% Al_2_O_3_ (meso-20AI NTs) show a response of 100 toward 97 ppm of NO*_x_* at room temperature with a response time of 28 s. This response time is 7.3-times higher than the pure In_2_O_3_ NTs. The response of the Al_2_O_3_–In_2_O_3_ composite NF sensor decreases in the order of meso-20AI NTs > meso-25AI NRs > meso-15AI NTs > pure In_2_O_3_ NTs > porous pure Al_2_O_3_ NRs. However, the response time increases in the order of pure In_2_O_3_ NTs > meso-25AI NRs > meso-15AI NTs > meso-20AI NTs > porous pure Al_2_O_3_ NRs [226].

**Figure 11:**
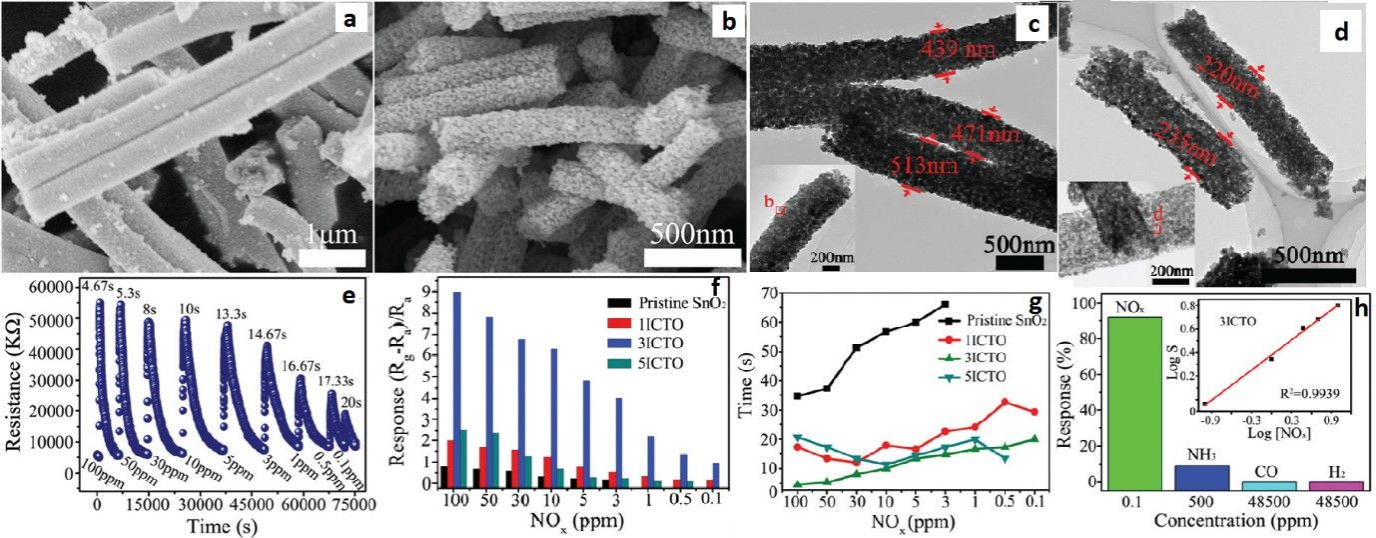
SEM images of (a) pristine SnO_2_ and (b) 3ICTO; TEM images of (c) pristine SnO_2_ NRs; (d) 3ICTO NRs; (e) dynamic response–recovery curves of the 3ICTO sensor for 100 ppm–0.1 ppm NO*_x_* at RT; (f) gas response and (g) response time for the four samples; (h) response of the 3ICTO sensor to different gases, the inset shows a linear dependence relation between the logarithm of the response and concentration of NO*_x_*. Reproduced with permission from [227], copyright 2015 Royal Society of Chemistry.

Xu et al. [227] synthesized In_2_O_3_ composite SnO_2_ (ICTOs) NRs using electrospinning for room temperature NO*_x_* sensing. Pristine SnO_2_ nanorods show an average diameter of 474 nm, whereas the average diameter of 3ICNO (Sn:In atomic ratio 25:0.3) is 230 nm. The TEM images (Figure 11a–d) show that NPs are connected through a neck between grains that improve electron conduction. The dynamic response of the 3ICTO sensor is shown in Figure 11e. The 3ICTO nanorods show the highest response of 8.98 toward 100 ppm of NO*_x_* at room temperature. The response time for 3ICTO is 4.67 s, that is, 11-times higher than pristine SnO_2_ nanorods. The composite nanorods detect 0.1 ppm of NO*_x_* with a response of 0.92 and response time 20 s (Figure 11e,f). The response time of all these sensors are shown in Figure 11g. The slowest response of 3ICTOs at 0.5 ppm is 35 s. The 3ICTO sensor shows a very high selectivity toward NO*_x_* in the presence of interfering gases as shown in Figure 11h.

**Figure 12:**
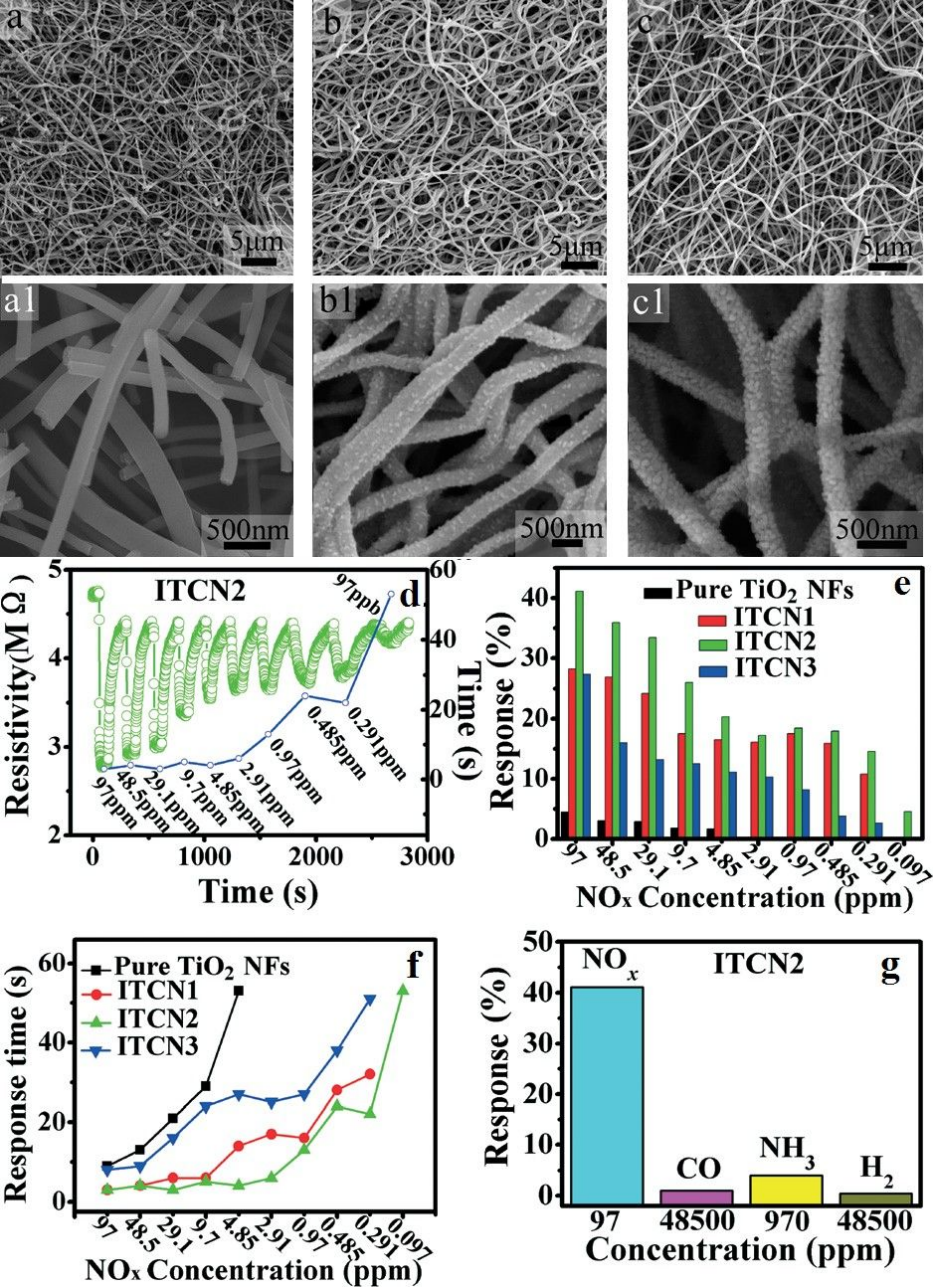
SEM images of (a), (a1) pure TiO_2_ NFs; (b), (b1) ITCN1; and (c), (c1) ITCN2. (d) The response–recovery cyclic curves vs the response time of ITCN2; (e) the bar graph represents the gas sensitivities of the samples; (f) the response time curves of the pure TiO_2_ NFs, ITCN1, ITCN2 and ITCN3; and (g) a bar chart showing the response value of the ITCN2 sensor for four different gases. Reproduced with permission from [228], copyright 2014 Royal Society of Chemistry.

P-type TiO_2_–In_2_O_3_ composite NFs have been synthesized by electrospinning for improved electrical conductivity and sensitivity to ppb levels of NO_2_ at room temperature [228]. Atomic ratios of Ti to In of 10:0; 8:1; 6:1; 5:1, are labelled as pure TiO_2_, ITCN1, ITCN2, and ITCN3, respectively. The pore size at the optimum atomic ratio of Ti and In 14.3 atom % (ITCN2) is 4–6 nm, whereas the average nanoparticle size is about 9 nm. The surface morphology of the TiO_2_–In_2_O_3_ composite NFs is shown in the Figure 12a–c. ITCN2 has an average diameter of 200 nm. The ITCN NFs show a loose mesoporous NF structure because nanoparticles are not closely bound in the centre of the fiber. The response time of ITCN2 is shown in Figure 12d. The highest response is 41.1% with a response time of 3 s for 97 ppm NO*_x_*. It is clear from the bar graph that the response of ITCN is better than pure TiO_2_ sensors. The lowest detectable concentration for ITCN2 is 97 ppb. The ITCN2 sample also shows the fastest response time at the lowest detectable concentration (97 ppb). The response time of ITCN2 is 53 s for 97 ppb at room temperature (Figure 12e,f). The responses of pure TiO_2_ NFs, and ITCN1, ITCN2 and ITCN3 for 97 ppb NO*_x_* at room temperature are 4.48%, 28.2%, 41.1%, 27.4% with response/recovery times of 9 s, 3 s, 3 s, and 8 s, respectively. The response of ITCN2 is 9.2-times higher than that of pure TiO_2_ NFs, whereas the response time of ITCN2 is 3-times faster than pure TiO_2_ NFs [228].

The Fe_2_O_3_–In_2_O_3_ composite NTs exhibit better formaldehyde sensing properties than other In_2_O_3_ composite NTs [229]. Fe_2_O_3_–In_2_O_3_ composite NTs have an average diameter of 200 nm. The mixing of Fe_2_O_3_ shows no effect on the diameter of the Fe_2_O_3_–In_2_O_3_ composite NTs. The composite NTs show almost two times higher response (33) for 100 ppm of formaldehyde at optimum temperature (240 °C) than pure In_2_O_3_ fibers with response and recovery times of 5 s and 25 s, respectively [229].

Similarly, ZnO–In_2_O_3_ composite NFs have been evaluated for trimethylamine (TMA) sensing [230]. The surface morphology and grain size of calcined ZnO–In_2_O_3_ composite NFs is dependent on composition of the NFs. For example, the grain size reduces with an increase in In_2_O_3_ content. The maximum response for ZnO–In_2_O_3_ composite NFs with the composition of Zn/In 67:33, 50:50, and 33:67 (atom %) to 5 ppm TMA is 133.9 at 300 °C, 82.9 at 350 °C, and 119.4 at 375 °C. The response of the ZnO–In_2_O_3_ composite nanofibers to TMA are up to 4.8-times and 12.0-times higher than those of pure ZnO and In_2_O_3_ nanofibers, respectively. The sensor with Zn/In 67:33 was found to be the best with respect to gas response, selectivity and sensing response speed [230].

Liu et al. [231] have synthesized SnO_2_/In_2_O_3_ hetero-NTs (SINs) using coaxial electrospinning for formaldehyde sensing. The samples with 0.1 g and 0.15 g of SnCl_2_.2H_2_O are labelled as SINs 0.1 and SINs 0.15, respectively. The SnO_2_/In_2_O_3_ hetero-NTs have an average diameter in the range of 80–120 nm. The NTs are composed of nanoparticles with diameter in the range of 10–50 nm. The hetero-NTs SINs 0.1 show very high response of 400 to 500 ppm of formaldehyde at optimum temperature (300 °C) with response and recovery times of 60 s and 97 s, respectively. The lowest possible concentration detectable by the sensors is 250 ppb with a response of 1.44 [231].

Du et al. [232] have synthesized SnO_2_/In_2_O_3_ composite hetero-NFs using modified bipolar electrospinning with a double jet modified by oxygen plasma. The morphology of the composite hetero-NFs changes significantly with this approach. The average diameter of the SnO_2_ NFs is in the range of 200–250 nm with a crystallite size of 20 nm. In_2_O_3_ NFs have an average diameter of 100–150 nm with a crystallite size of 40–50 nm. The surface of SnO_2_, In_2_O_3_, and SnO_2_/In_2_O_3_ NFs become rough and their diameter increases to 450 and 500 nm after treatment with oxygen plasma. The optimum temperature also reduces from 375 °C to 290 °C after plasma treatment. The oxygen-plasma-treated composite NFs show a higher response of 35.69 toward 50 ppm formaldehyde at optimum temperature and a low detection limit of 0.5 ppm formaldehyde is measured. Moreover, treated composite fibers exhibit response and recovery times of about 20 s and 40 s, respectively.

**Figure 13:**
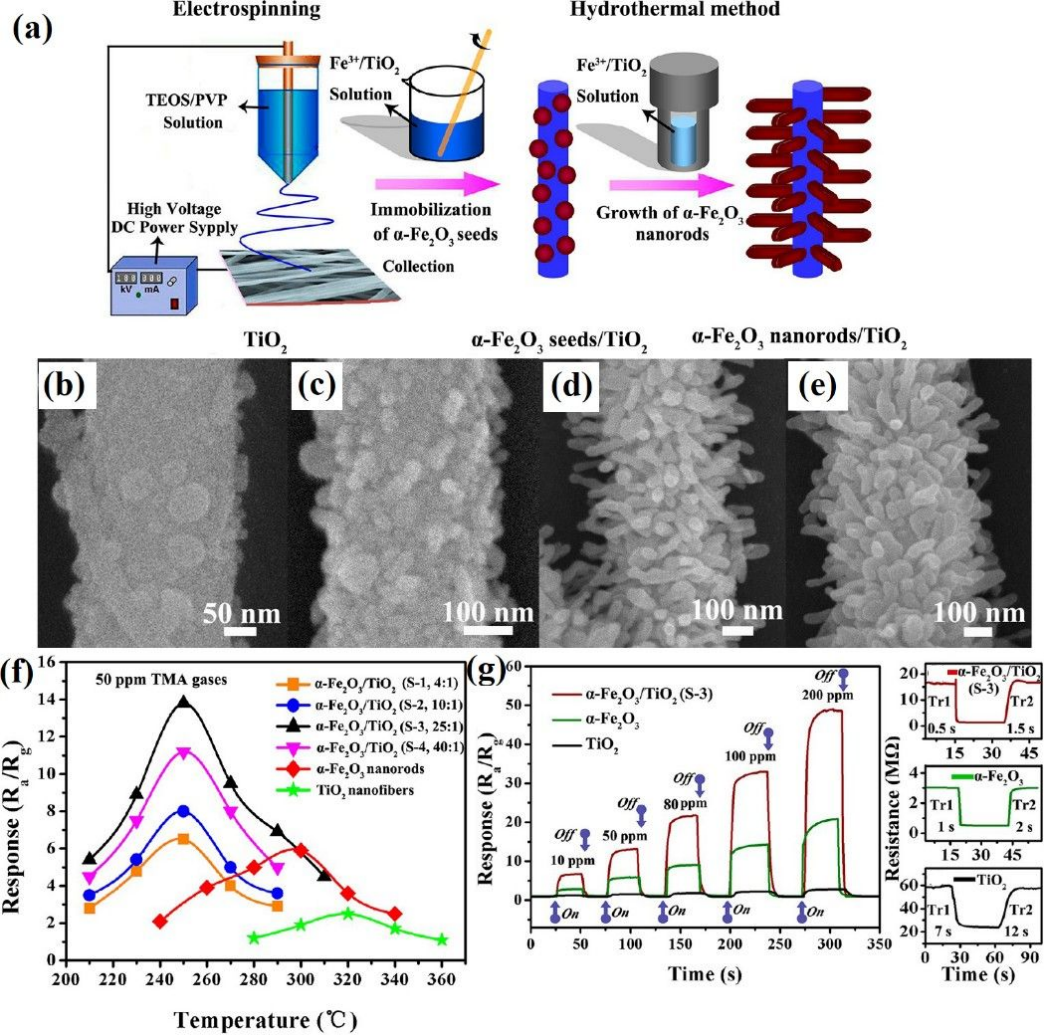
(a) Synthesis strategy for branch-like α-Fe_2_O_3_/TiO_2_ hierarchical heterostructures; FESEM images of hierarchical α-Fe_2_O_3_/TiO_2_ heterostructures with different molar ratios: (b) S-1, 4:1, (c) S-2, 10:1, (d) S-3, 25:1; and (e) S-4, 40:1; (f) effect of operating temperature on different molar ratios as well as pristine α-Fe_2_O_3_ nanorods, and TiO_2_ nanofibers, and (g) dynamic TMA-sensing response curves and response/recovery time of the pristine α-Fe_2_O_3_ nanorods, TiO_2_ nanofibers, and α-Fe_2_O_3_/TiO_2_ heterostructures. Reproduced with permission from [234], copyright 2013 ACS.

Electrospun ZnO–TiO_2_ NFs have been converted to 3D hierarchical heterojunctions composed of highly dispersed ZnO nanorods by a hydrothermal process and applied to ethanol sensing [233]. The ZnO–TiO_2_ heterojunction NFs have a brush-like morphology. The average diameter of TiO_2_ NFs and ZnO nanorods are about 100 and 300 nm, respectively. The ZnO NRs of about 2 µm length grow on the outer surface of TiO_2_ NFs. A ZnO–TiO_2_ sensor shows a fast response with a maximum value of 50.6 at the operating temperature of 320 °C, whereas the response and recovery times are 50.6 and 5–10 s to 500 ppm of ethanol [233]. Similar branch-like nanostructures of α-Fe_2_O_3_ nanorods/TiO_2_ nanofibers have been fabricated using electrospinning technique and hydrothermal process [234] (Figure 13). The morphology of α-Fe_2_O_3_/TiO_2_ hierarchical heterostructure nanofibers changes drastically when changing the molar ratio between α-Fe_2_O_3_ and TiO_2_ precursors. The SEM images for the different molar ratios are shown in the Figure 13b–e. Furthermore, these hierarchical branch-like α-Fe_2_O_3_/TiO_2_ structures have been applied to gas sensing applications because of their rough, loose, and well-aligned surface morphology, including a branch-like heterostructure. The morphology–operating temperature behaviour relation has also been studied and is shown in Figure 13f. The maximum response of four α-Fe_2_O_3_/TiO_2_ sensors at optimum temperature 250 °C is (S-1) 6.5, (S-2) 8.0, (S-3) 13.9, and (S-4) 11.2, respectively. The developed hierarchical branch-like α-Fe_2_O_3_/TiO_2_ materials also show rapid response/recovery times of 0.5 s and 1.5 s compared with their counterparts [234].

**Figure 14:**
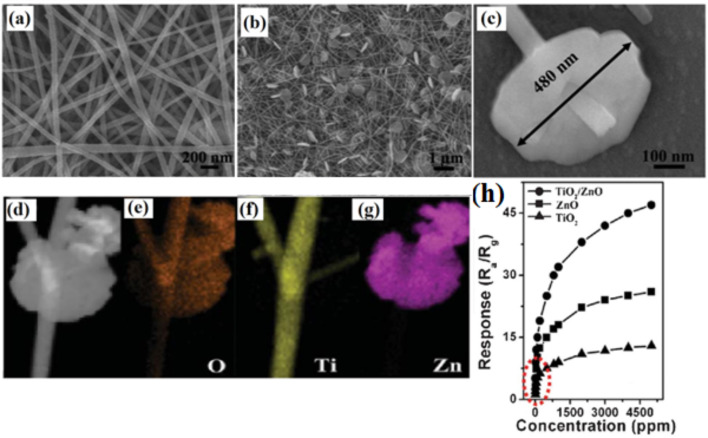
SEM images of (a) pure TiO_2_ NFs and (b,c) TiO_2_/ZnO heterostructures. (d–g) STEM image of TiO_2_/ZnO heterostructures; (h) response of TiO_2_/ZnO heterostructure nanofibers at 280 °C, pure ZnO at 300 °C and pure TiO_2_ at 350 °C versus ethanol concentration. Reproduced with permission from [235], copyright 2013 Royal Society of Chemistry.

Lou et al. [235] report TiO_2_/ZnO composite nanostructures that show minimal response at a lower temperature of 280 °C compared with the brush-like hierarchical heterojunctions. The TiO_2_/ZnO sensor shows a higher response of 15.7 to 100 ppm of ethanol at 280 °C compared with that of pure ZnO (9.7) and TiO_2_ NFs (5.0). Lou et al. [235] have also proposed a heterostructure comprising ZnO nanosheets on TiO_2_ NFs for this sensor. The morphology of the heterostructures is shown in Figure 14a–c. The diameter of the NFs is about 70–100 nm with nanosheets uniformly distributed over the surface of TiO_2_ NFs. The operating temperature has been significantly reduced with the TiO_2_/ZnO heterostructure when compared to pure TiO_2_ NFs or ZnO NFs. The response of the TiO_2_/ZnO heterostructure is about 5.2, 9.4, 12.5, 15.7, and 19.6 to 10, 20, 50, 100 and 200 ppm of ethanol, respectively (Figure 14h). The response and recovery times for TiO_2_/ZnO are about 5/3 s, which compares with 7/13 s and 6/3 s for the pure ZnO and TiO_2_ NFs, respectively [235].

Feng et al. [103] report on In_2_O_3_–WO_3_ heterojunction NFs for acetone sensing. The molar percentage of 0, 0.5, 1.5, and 3% In_2_O_3_–WO_3_ heterojunction NFs are labelled as S1, S2, S3, and S4. The average diameter of almost all samples is 170 nm. The largest response is exhibited by a sensor with a molar ratio of In_2_O_3_/WO_3_ 1.5:100 for 50 ppm of acetone at 275 °C. The largest response is 12.9 for 50 ppm of acetone which is 2.5-times higher than the pure WO_3_ NFs. The lowest possible detectable concentration is 0.4 ppm of acetone with a response of 1.28. The response and recovery times are about 6 s and 64 s for the S3 sensor [103].

In_2_O_3_ NFs coated with SnO_2_ nanoparticles have been synthesized by combining electrospinning and sol–gel processing in order to test for ammonia sensing [236]. The atomic percentage of SnO_2_ is about 0%, 7.5 atom %, 16 atom %, and 21 atom % in In_2_O_3_, SnO_2_/In_2_O_3_-1, SnO_2_/In_2_O_3_-2 and SnO_2_/In_2_O_3_-3 NFs, respectively. The SnO_2_ NPs coated onto In_2_O_3_ NFs show an average diameter in the range of 30–80 nm. The highest response of 21 is shown by SnO_2_/In_2_O_3_-2, toward 1 ppm of ammonia with response and recovery times as 7s and 10 s, respectively. The fabricated SnO_2_ NP-coated In_2_O_3_ NFs are able to detect 0.1 ppm of ammonia [236].

Photoactivated TiO_2_/Pd/N/Fe_2_O_3_ composite fibers have been applied to H_2_ sensing [237]. The TiO_2_ nanofibers have an average diameter of 70 ± 20 nm and their surface is smooth with few cracks. The operating temperature is lowered from 290 °C to 130 °C by using visible-light irradiation and additive materials. The response of the sensor increases from 11 (pure TiO_2_ NFs) to 368 (TiO_2_/Pd/HNO_3_/Fe_2_O_3_/UV_2_ NFs of 450–470 nm diameter) at an operating temperature of 150 °C. The detection limit for hydrogen sensing is 25 ppm for pure TiO_2_ NFs but reduces to 0.5 ppm for a TiO_2_/Pd/HNO_3_/Fe_2_O_3_/UV_2_ sensor. The response time also reduces from 25 s to 0.9 s and recovery times are reduced from 40 s to 2 s. The Pd concentration is optimized (Pd/TiO_2_ = 9% molar ratio) by observing the sensor response in dark and UV irradiation. At 150 °C, the response time is 25, 9, 5, and 0.9 s and recovery time is 40, 22, 10, and 2s for the pure TiO_2_, Pd-TiO_2_, TiO_2_/UV_1_ (360–390 nm), and Pd-TiO_2_/UV_2_ (400–420 nm) samples, respectively [237]. A detailed analysis of MOx sensors developed with different MOx materials is shown in Table S4 in Supporting Information File 1.

**4.1.4 Conducting polymer–semiconducting metal oxide composites:** Gas sensors based on conducting polymers have shown excellent electronic conductivity and electrochemical properties [32,87,90]. Conducting polymers are organic materials that show an enhanced resistivity toward external stimuli. These conducting polymers show chemical selectivity, which allows them to act as excellent materials for gas sensors. Gopalan et al. [88] used electrospinning to produce composite NFs by poly(diphenylamine) and poly(methyl methacrylate) (PMMA) for sensing applications. Sensors developed in this way can detect 10 ppm of ammonia at room temperature with a linear response from 10 to 300 ppm.

**Figure 15:**
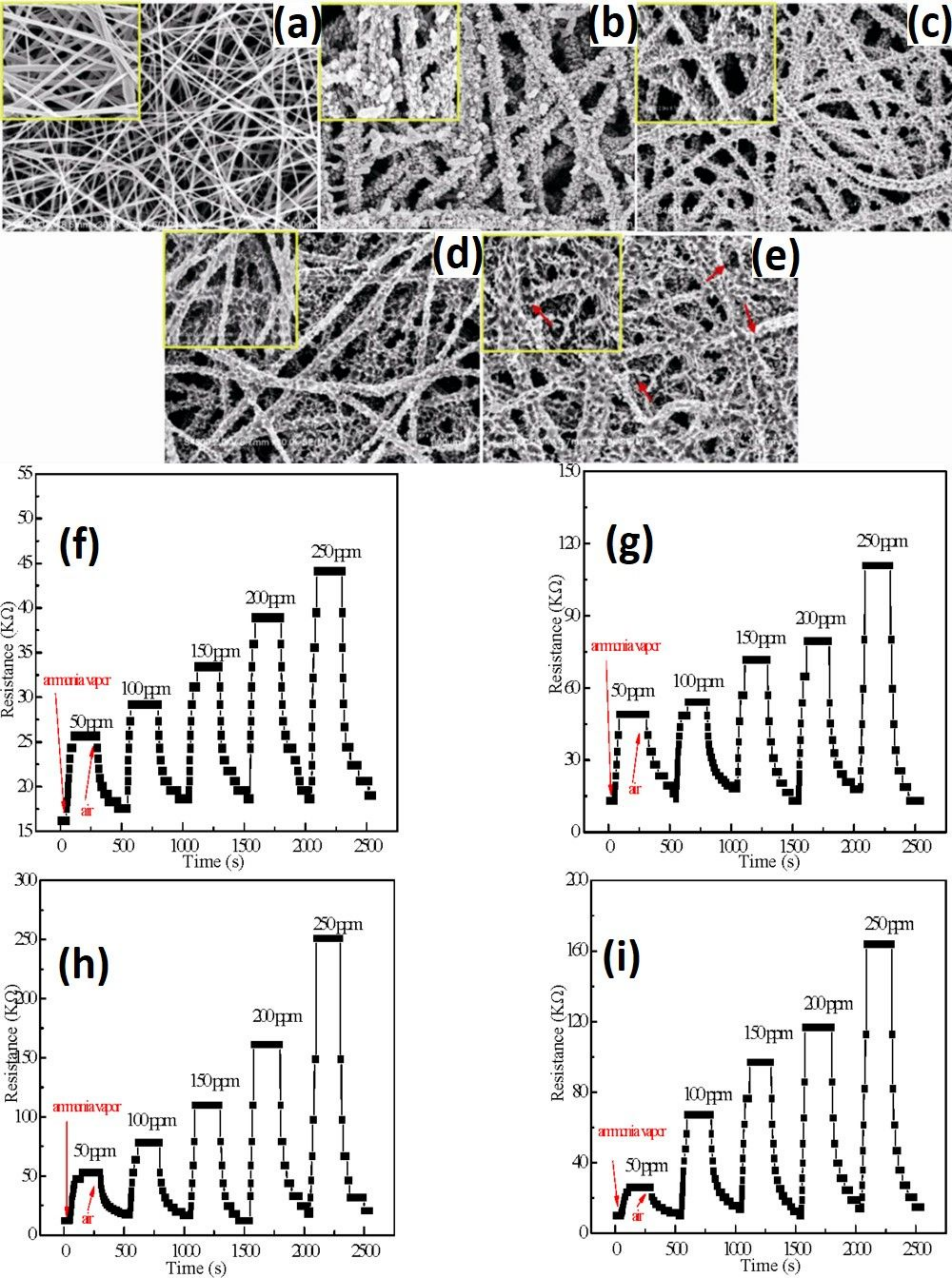
SEM image of (a) PA6 NFs; (b) PANI/PA6 NFs; (c) PANI/PA6 NFs sputtered for 30 min; (d) TiO_2_–PANI/PA6 NFs sputtered for 60 min; (e) TiO_2_–PANI/PA6 NFs sputtered for 90 min; dynamic response and recovery of (f) PANI/PA6; (g) TiO_2_–PANI/PA6 sputtered for 30 min; (h) TiO_2_–PANI/PA6 sputtered for 60 min and (i) TiO_2_–PANI/PA6 sputtered for 90 min to ammonia vapour of different concentrations. Reproduced with permission from [238], copyright 2012 MDPI.

Ji et al. [87] produced PMMA NFs and their composites with polyaniline (PANI) using electrospinning and in situ solution polymerization. The sensor displays a sensing magnitude of 77 toward triethylamine (TEA) vapour of 500 ppm at room temperature. A linear, reversible and reproducible response to TEA vapours with different concentrations (20–500 ppm) is observed. The doping acid concentration only changed the sensor resistance and did not have any influence on the sensing performance. For example, the sensor with toluene sulfonic acid as the doping acid exhibits the highest sensing magnitude (77) toward 500 ppm of TEA. Similarly, composites of PANI and metal oxides have been synthesized by electrospinning and applied to gas sensing. Wang et al. [238] synthesized polyaniline/polyamide 6 (PANI/PA6) composite NFs by in situ polymerization and electrospinning. Later, TiO_2_–PANI/PA6 composite NFs were fabricated by RF magnetron sputtering. The SEM images of the resulting fibers are shown in Figure 15a–e. The PANI/PA6 and TiO_2_–PANI/PA6 NFs have uniform diameter with a rough surface. The morphology of the PANI/PA6 fibers is distorted by the TiO_2_ deposition and this distortion becomes prominent at 90 min sputtering time. The effect of TiO_2_ nanoparticles on the dynamic response of the sensor is shown in Figure 15f–i. The sensors with 60 min sputtering time performed the best with the highest value of response as measured by resistance value (Figure 15h).

Polyaniline/titanium dioxide (PANI/TiO_2_) composite NFs have been prepared by electrospinning and used for NH_3_ sensing [239]. In this case, Mn_3_O_4_/TiO_2_ fibers are fabricated by electrospinning and, in a later stage, oxidatively polymerized aniline is prepared with Mn_3_O_4_. SEM images of the electrospun Mn_3_O_4_/TiO_2_ composite NFs and the as-obtained PANI/TiO_2_ composite NFs show that the diameter of the Mn_3_O_4_/TiO_2_ fibers is greater than the PANI/TiO_2_ NFs. This size difference might be due to the conversion of Mn_3_O_4_ to PANI. The highest sensitivity is for the sample which has an Mn to Ti ratio of 3:1. If the ratio of Mn to Ti is below this optimal value, the sensitivity increases with Ti content in the composite. However, if the ratio is above the optimal value, the sensitivity decreases with Ti content in the composite [239].

Core–shell titania–poly(3,4-ethylenedioxythiophene) (TiO_2_-PEDOT) nanocables have been prepared by electrospinning of TiO_2_ combined with vapour phase polymerization of PEDOT [240]. TiO_2_ used as a template serves as a core with an average diameter of 78 nm and with a PEDOT sheath of about 6 nm. The prepared nanocables are used for NO_2_ and NH_3_ sensing [240]. The detection limit for NO_2_ is 7 ppb, whereas for NH_3_ the limit is 675 ppb.

Li et al. [241] synthesized SnO_2_ nanofibrous sheets (NSs) coated with polypyrrole (PPy) for NH_3_ sensing at room temperature. The vertically aligned SnO_2_ NSs are grown by hydrothermal treatment at 135 °C and then coated with PPy by vapour phase polymerization of pyrrole. The SnO_2_ nanofibers have an average diameter in the range 100–200 nm. The NSs were further doped with compounds such as hydrochloric acid (HCl), (±)-10-camphorsulfonic acid (CSA), *p*-toulenesulfonic acid (TSA) and poly(styrene sulfonic acid) (PSSA). The order of sensitivity of these dopant acids is PSSA > HCl > TSA > CSA. The nanocomposite sensors exhibit sensitivity of ≈6.2 %/ppm in the range of 1–10.7 ppm of NH_3_ and successfully detect low concentrations of NH_3_ (257 ppb). The response and recovery times for SnO_2_/PPy nanocomposites toward 5 ppm of NH_3_ are 259 s and 468 s, respectively. Similarly, SnO_2_ NSs have also been doped with PANI and tested for NH_3_. As with the nanocomposites, SnO_2_ NFs/PANI sensors show a high response (relative resistance change of ≈3700% toward 10.7 ppm of NH_3_) and a detection limit of 46 ppb at room temperature [242].

**Figure 16:**
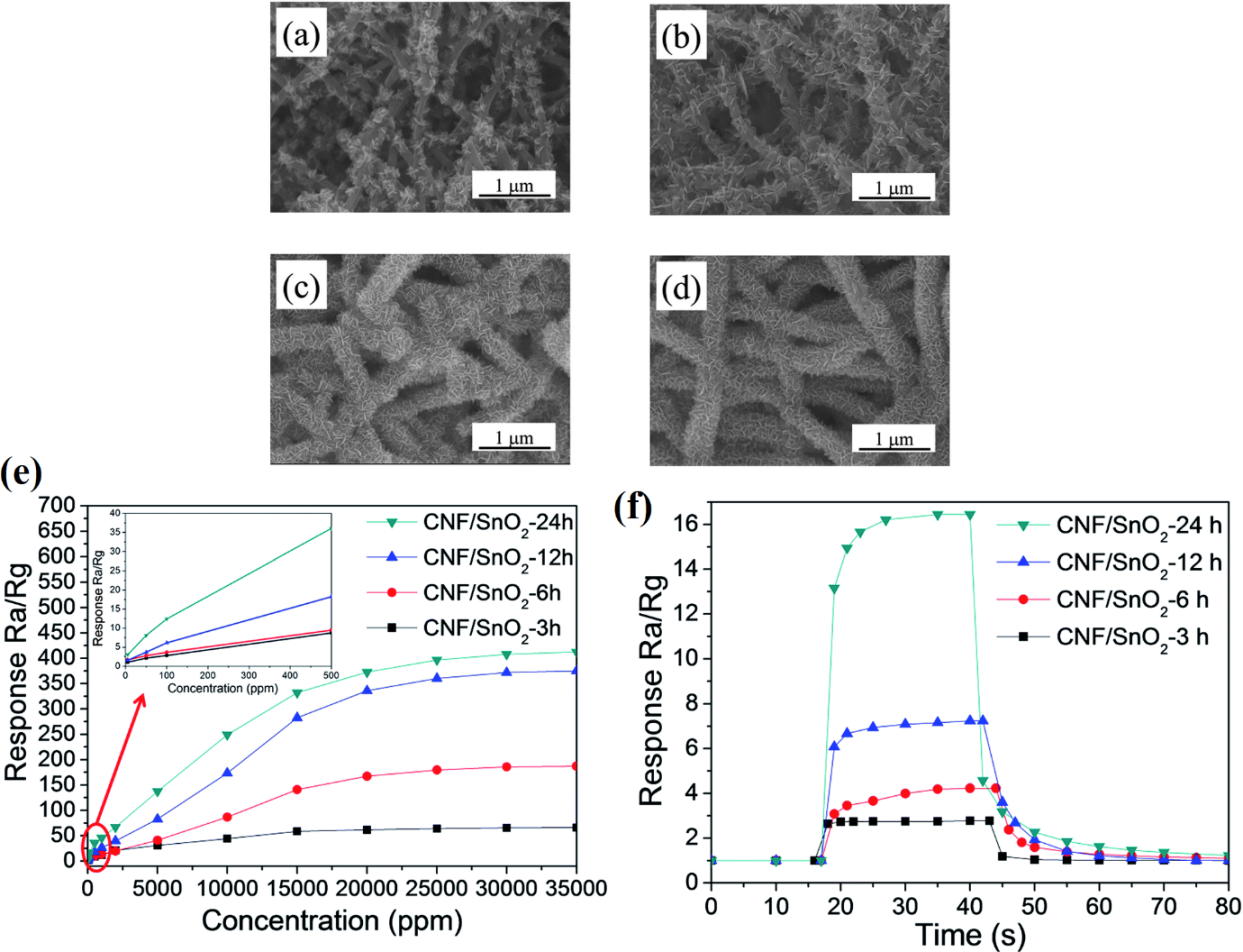
(a) SEM images of the hierarchical SnO_2_/PAN p–n junction nanostructures at different hydrothermal reaction times (a) 3 h; (b) 6 h; (c) 12 h; and (d) 24 h. (e) Linear plots of the response of the sensors based on the hierarchical p–n junction nanostructures with different hydrothermal reaction time against H_2_ in the range of 5 ppm – 3.5% at 200 °C. (f) Response and recovery behaviour of the hierarchical p–n junction nanostructures with different hydrothermal reaction times at 100 ppm H_2_. Reproduced with permission from [243], copyright 2015 Royal Society of Chemistry.

Wang et al. [243] synthesized hierarchical p–n junction nanostructures made of n-type SnO_2_ NSs standing on p-type carbon nanofibers by combining electrospinning and hydrothermal treatment. The morphology of the heterostructures is shown in Figure 16a–d. The average diameter of the SnO_2_/PAN NFs is in the range of 150–500 nm. The size of SnO_2_ NSs is controlled by the reaction time during hydrothermal treatment. The maximum response is obtained at 200 °C with the highest response from a sample with 24 h hydrothermal reaction time (Figure 16e). The SnO_2_/PAN NSs exhibit a maximum response of ≈16.3 at 200 °C for 100 ppm of hydrogen with response and recovery times of 4 s and 16 s, respectively (Figure 16f) [243].

Sn-SnO_2_/PAN heterostructure NFs have been synthesized for ethanol sensing by electrospinning followed by annealing [244]. The Sn-SnO_2_/PAN NFs have an average diameter in the range of 350–400 nm after heat treatment. The Sn-SnO_2_/PAN NFs show a maximum response of 46.15 at 220 °C toward 1000 ppm of ethanol compared with pure SnO_2_ NFs with 15.16 at 280 °C [244].

**4.1.5 Graphene–semiconducting metal oxide composite:** Reduced graphene oxide (rGO) nanosheets (NSs) can be incorporated into MOx NFs to improve their sensitivity and selectivity. Abideen et al. [89] have produced rGO/NS-loaded ZnO NFs that comprise nanograins with an average diameter of 20 nm. These rGO/NS-loaded ZnO NF-based gas sensors show an excellent sensitivity to CO, C_6_H_6_, and C_2_H_5_OH. The amount of rGO is optimized and the sensor containing 0.44 wt % rGO NSs shows a higher response (*R*_a_/*R*_g_ ≈ 119) toward 5 ppm of NO_2_ than the other sensors. Moreover, the same sensor shows a response of 22.6, 19.1, and 19.1 toward 5 ppm of CO, C_6_H_6_, and C_2_H_5_OH, respectively. The response and recovery times for 1 ppm of NO_2_ are 174 s and 107 s, respectively [89].

Furthermore, Abideen et al. [245] have also synthesized graphene NS-loaded SnO_2_ NFs using electrospinning and optimized the amount of graphene in SnO_2_ NFs for gas sensing. The average diameter of the NFs is in the range 200–300 nm, whereas the grain size is significantly affected by the graphene content. The graphene NS-loaded SnO_2_ NFs achieve a maximum response of 3.13 at 300 °C, whereas pristine SnO_2_ NFs show a maximum response of 1.94 at 325 °C. The graphene NS-loaded SnO_2_ NFs show a very short response time of 51.2 s at optimum graphene content (0.5 wt %) [245].

**Figure 17:**
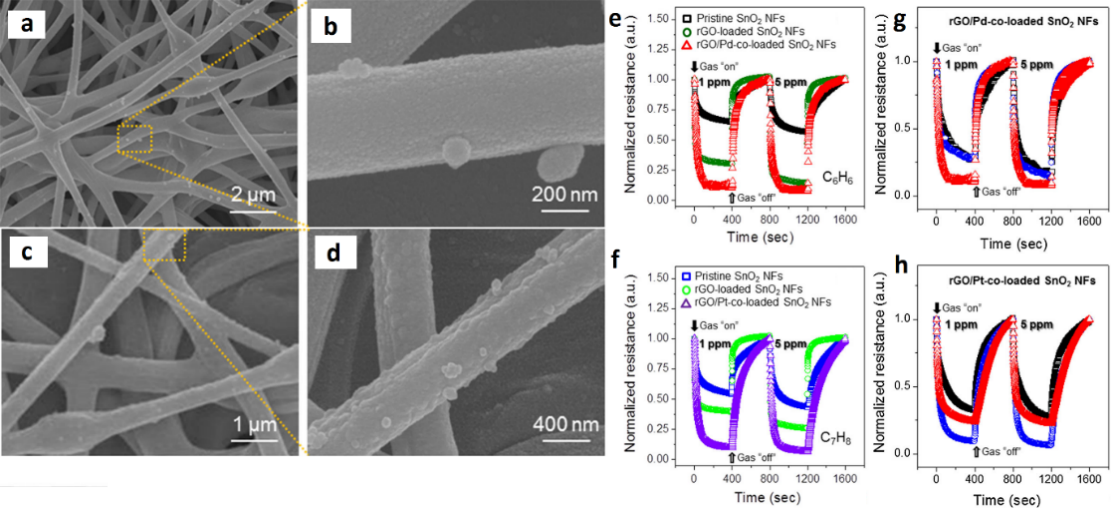
rGO/Pd co-loaded SnO_2_ NFs: (a,c) low- and (b,d) high-magnification FE-SEM images. (e) Normalized dynamic resistance for pristine SnO_2_ NFs, rGO-loaded SnO_2_ NFs, and rGO/Pd co-loaded SnO_2_ NFs in the presence of 1 ppm and 5 ppm C_6_H_6_ at 200 °C. (f) Normalized dynamic resistance for pristine SnO_2_ NFs, rGO-loaded SnO_2_ NFs, and rGO/Pt co-loaded SnO_2_ NFs in the presence of 1 ppm and 5 ppm C_7_H_8_ at 200 °C. (g) Normalized dynamic resistance for rGO/Pd co-loaded SnO_2_ NFs toward 1 ppm and 5 ppm CO, C_6_H_6_, and C_7_H_8_ gases at 200 °C. (h) Normalized dynamic resistance for rGO/Pt co-loaded SnO_2_ NFs toward 1 ppm and 5 ppm CO, C_6_H_6_, and C_7_H_8_ gases at 200°C. Reproduced with permission from [246], copyright 2017 Springer.

Kim et al. [246] have synthesized (Pt or Pd) co-loaded SnO_2_ NFs containing rGO using electrospinning. They have also compared the gas sensing properties of (Pt or Pd) co-loaded SnO_2_ NFs with rGO-loaded SnO_2_ to 1 ppm and 5 ppm of C_6_H_6_, C_7_H_8_, and CO. The SEM images of the NFs and their gas sensing performance are shown in Figure 17. Pt or Pd nanoparticles with a diameter of 50–200 nm are dispersed on the surface of NFs as shown in the high-magnification SEM image (Figure 17b). The response of pristine SnO_2_ NFs, rGO-loaded SnO_2_ NFs, and rGO/Pd co-loaded SnO_2_ NFs is 1.6, 3.3, and 8.3 for 1 ppm of C_6_H_6_, respectively at 200 °C [246].

#### 4.2 Surface acoustic wave gas sensors

The surface acoustic wave (SAW) based sensor relies on detecting the change in the velocity of an acoustic wave on a piezoelectric substrate surface caused by the adsorption of analytes. Liu et al. [247] developed electrospun polyethylene (PEO) nanofibrous membrane based SAW gas sensors for detection of toluene, H_2_O_2_, isopropanol and nitrobenzene. PEO NFs were fabricated on an ST-cut quartz (42° angle with *z*-axis) SAW sensor. The PEO NFs have a diameter in the range of 100–300 nm with a sensing layer thickness of 8.1 µm. The normalized frequency shift of the developed sensor is −767, −343, −357, and −537 toward 90% saturated concentration of isopropanol, H_2_O_2_, toluene, and nitrobenzene at room temperature. The response of the developed sensor is very fast with 1 min of adsorption, 2 min of diffusion, and 1 min of desorption time.

**Figure 18:**
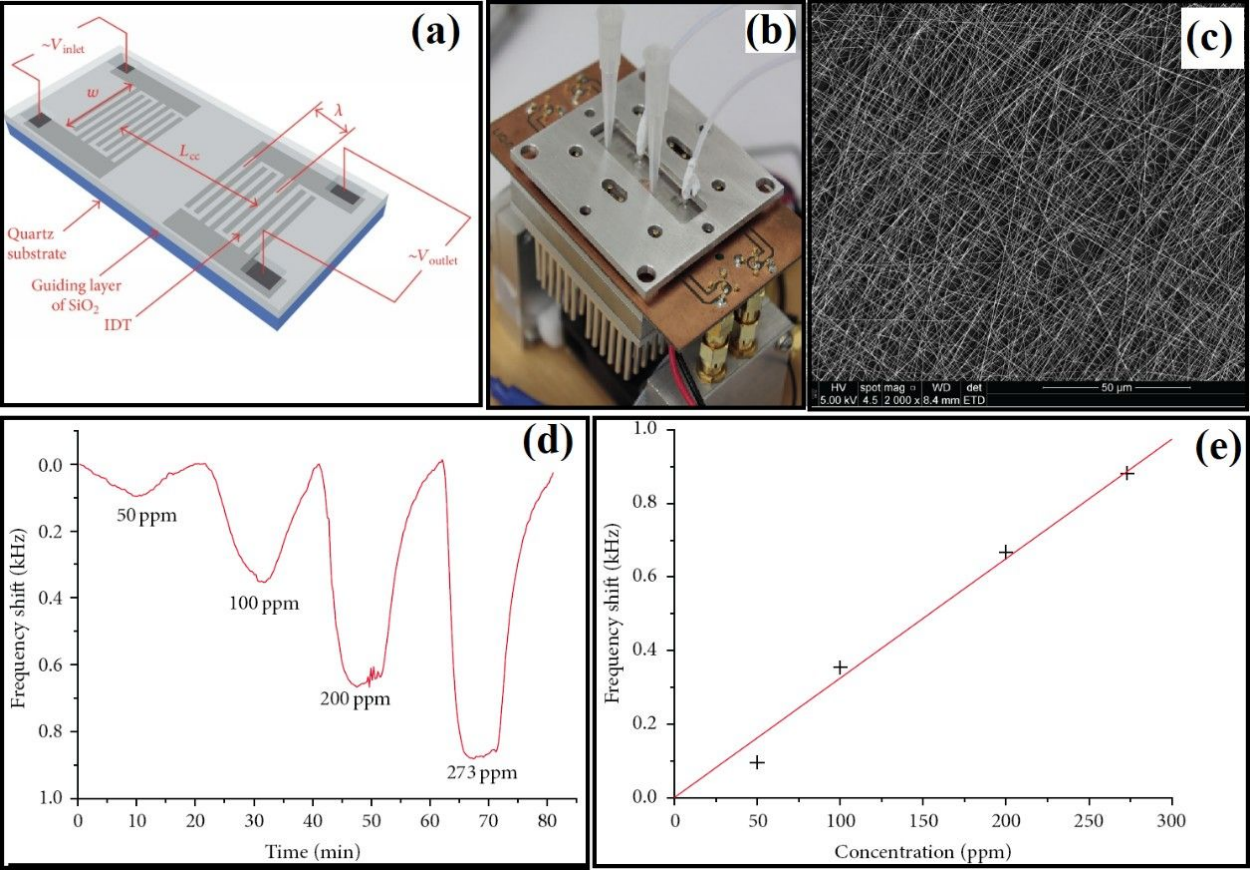
(a) 3D scheme representation; (b) real experimental setup of a Love-wave sensor with two RF ports, layer composition, and geometrical parameters; (c) SEM images of SAW resonators deposited with electrospun PVP nanofiber; (d) dynamic frequency shift of the SAW sensor with a sensitive layer of electrospun PVP nanofibers for different concentrations of toluene and (e) the linear relation between frequency shift and the concentration [250]. Images reproduced from [250], copyright 2017 Matatagui et al.

A humidity sensor has been fabricated with PANI/poly(vinyl butyral) (PVB) composite nanofibers deposited on a SAW resonator with a central frequency of 433 MHz [248]. Nanofibers synthesized from several polymers with different hydrophilicity, and viscoelasticity are tested as a template. PVB nanofibers with an average diameter of 100–200 nm show the maximum frequency shift. A frequency shift of 3.428 MHz and 8.134 MHz, respectively, has been measured for PEO and PVP nanofibers. For the same humidity levels, a much lower sensitivity was obtained for PMMA, poly(vinylidene fluoride) (PVDF) and PVB with frequency shifts of 0.312 MHz, 0.334 MHz and 0.425 MHz, respectively [248]. In SAW humidity sensors, the frequency shift response is predominantly due to changes in mass as well as electroacoustic and viscoelastic load of the sensing layer upon exposure to water molecules. A high humidity sensitivity (≈0–75 kHz/%RH) and an ultrafast response (1 s and 2 s for humidification and desiccation, respectively) is reported for composite nanofibers [248]. Moreover, the sensor is able to detect humidity as low as 0.5% RH [248]. Electrospun PVP ﬁbres on a 36° LiTaO_3_ SAW sensor have been applied to hydrogen sensing [249]. The sensor shows a recovery time of 200 s upon exposure to hydrogen for 120 s. A maximum frequency shift of 5.6 kHz for 1% hydrogen is measured. Response of the electrospun fibers from 56% and 58% PVP solutions was similar and higher than response for fibers with lower concentrations of PVP [249]. Similarly, electrospun PVP has been applied as a sensitive layer on SAW sensor for VOCs sensing (Figure 18a,b) [250]. The PVP fibers deposited on a SAW device is shown in Figure 18c. The PVP fibers have an average diameter of 120 nm with a film thickness in the range of 1.5–5 µm at 18 KV and 22 cm. The developed sensor has been tested for different concentrations of toluene (50, 100, 200, and 273 ppm) as shown in Figure 18d,e. The minimum frequency shift that could be detected is 30 Hz. The slope of the fit in Figure 18e shows the sensitivity of 3.25 Hz/ppm that leads to a detection limit of 10 ppm [250].

CeO_2_ NPs coated with PVP nanofibers based on low and high frequency SAW resonators operating at 879 MHz (LF) and 1.56 GHz (HF), respectively, have been fabricated for RH detection [251]. The samples of 0.1, 0.5, 1 and 2 mmol CeO_2_ with constant 300 mg PVP are denoted as PC0.1, PC0.5, PC1 and PC2. SEM images of these CeO_2_ NPs reveal a size range of 400–480 nm along with CeO_2_/PVP nanofibers (diameter 450 nm). The sensor PC1-LF exhibits the maximum shift of −300 kHz, while PC1-HF shows an enhanced frequency shift (−2.5 MHz). The frequency shift of the SAW humidity sensor using the same sensitive material increases as the resonant frequency is raised with a maximum frequency shift of PVP-HF of −2.09 MHz. Moreover, PC1-HF does not exhibit any remarkable deterioration in frequency response and maintains its sensing characteristics (frequency shift of approximately −2.3 MHz from 11% to 95% RH) [251].

The humidity sensing performance of electrospun multiwalled carbon nanotube (MWCNTs)/nafion composites based on SAW devices was also investigated by Sheng et al. [252]. The MWCNTs had an average diameter in the range of 10–20 nm. Nafion appears as white-grey beads around MWCNTs in composite fibers. The resonance frequency of the sensor decreased with an increase in humidity level. The sensor showed sensitivity up to 427.6 kHz/% RH with excellent linearity (R2 > 0.98) in the range from 10% RH to 80% RH. The dynamic response of the sensor indicates a very short response time <3 s [252].

#### 4.3 Quartz crystal microbalance gas sensors

Gas sensors based on a quartz crystal microbalance (QCM) offer superior sensitivity and resolution compared to other types of sensors because frequency is a quantity that can be measured with a very high degree of accuracy and precision [252–253]. QCMs are cost-effective and eliminate the need for time-consuming sample preparation to suit a particular type of transducer. Other benefits of QCM sensors include room temperature operation and simple packaging requirements. QCMs are usually fabricated from thin disks of quartz, with circular electrodes patterned on both sides, onto which electrical signals are applied. The piezoelectric crystal transforms the electric signal applied on the metal pads to acoustic waves. In a simplified model from Sauerbrey [254], the wavelength of the oscillation is half the crystal thickness. The natural frequency of the resonant acoustic waves is determined by the crystal thickness. When a mass is deposited on the crystal, it increases the thickness, increasing the wavelength of the acoustic waves, i.e., decreasing the frequency. The relationship between the change in the oscillation frequency, Δ*f*, of a QCM to the change in mass added to the surface of the crystal, Δ*m*, is given by the Sauerbrey equation [12,253–254]:


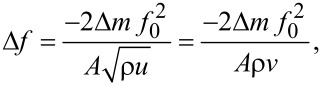


where *f_0_* is the resonant frequency of the crystal, *A* is the area of the crystal, and ρ, *u* and ν are the density, shear modulus and shear wave velocity of the substrate, respectively. As can be seen, any variations in mass changes the oscillating frequency, making the QCM suitable for sensing applications. The QCM response is measured as a change in the frequency per change in mass on the device unit area. As the operational frequency increases, or as the crystal thickness decreases, the QCM sensitivity increases. For example, the mass detection limit for a 10 MHz QCM can be measured to about less than 1 ng/cm^2^ [255].

**4.3.1 Non-conducting polymer:** A PVP fibrous membrane based QCM device has been used for ethanol sensing [256]. The sensitivity of the sensor is improved by increasing the PVP concentration with a maximum response at 12% PVP and then sensitivity decreases at higher concentrations. For low concentrations of PVP, the fibers show beads that reduce the overall surface area. On the other hand, higher PVP concentrations (>12%) resulted in thicker fibers and the surface area reduces with a corresponding decrease in gas sensing response. As the vapour concentration increases, the sensitivity increases for two distinct patterns: a Langmuir pattern for a low concentration (5–15 mg/L) and convex for high concentrations (>17.5 mg/L) of the ethanol vapour. The surface area can be increased by increasing the thickness of fibrous membrane on the QCM, but diffusion of the gas takes more time with a thick porous layer and results in an increase in response time. The number of adsorption sites and sample porosity are enhanced by increasing the thickness of the fibrous layer [256].

Nanoporous polystyrene (PS) fibrous membrane functionalized by the polyethyleneimine (PEI) deposited on the QCM has been used for formaldehyde sensing [257]. The morphology and surface area of the fibrous PS membranes with fiber diameter of 110–870 nm are controllable by tuning the concentrations of PS solutions. PEI particles are found in clusters on the surface of a bead-on-string morphology after being functionalized by PEI. The fibers formed from 7 wt %, 10wt % and 13 wt % PS solution have a bead-on-string morphology consisting of thin fibers (average diameter of 266 nm, 294 nm and 500 nm, respectively) with numerous micrometre-sized beads along the fiber axis. This morphology may be due to the low viscosity of the solution used for electrospinning. On the other hand, beads are preferred in order to prevent separation between the fibrous membrane and the QCM electrode. The high-resolution images of the NFs show that fibers have well developed nano-textures with a rough surface morphology and the beads also show a porous structure. PS fibers that are formed from 10 and 13 wt % PS solutions show surface area values of 37.23 m^2^/g and 47.25 m^2^/g, respectively. The NFs from the 13 wt % PS solution have the largest fiber diameter but also have the highest surface area because of a porous structure. PEI particles with diameter of 50 nm to 1.2 µm are randomly distributed over the surface of fibrous PS membranes [257].

The maximum frequency shifts of the QCM-based PEI–PS (7 wt %) sensor exposed to 10, 30, 70, and 140 ppm of formaldehyde are 7, 8, 14, and 19 Hz, respectively. The sensor based on PEI–PS with 10 and 13 wt % NFs shows a maximum frequency shift of 7 and 15 Hz, respectively. On the other hand, the maximum frequency shifts of the QCM-based PEI–PS (7, 10, and 13 wt %) sensors are 19, 43, and 75 Hz, respectively for 140 ppm of formaldehyde. When exposed to 140 ppm of formaldehyde, the maximum frequency shifts of the QCM-based PEI–PS sensors with various PEI coating loadings (1000, 2000, and 6000 Hz) are 5, 33, and 75 Hz, respectively. The developed sensor also shows excellent selectivity for formaldehyde [257].

**Figure 19:**
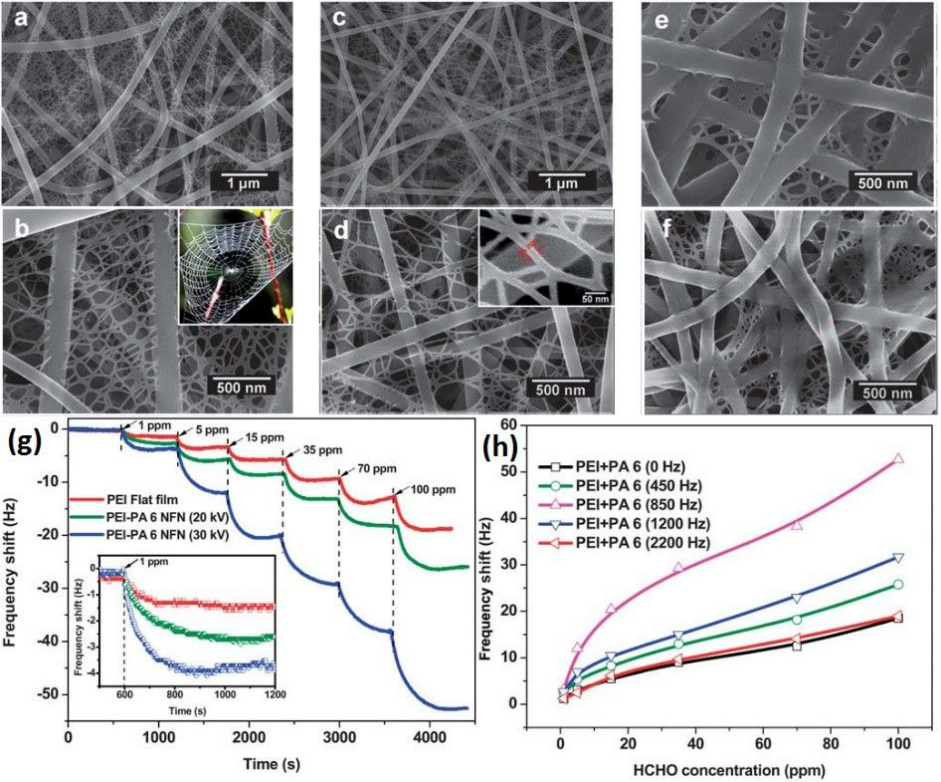
FE-SEM images of PA 6 NFN membranes formed with different voltages: (a, b) 20 kV and (c, d) 30 kV; and their corresponding samples modified with PEI: (e) PEI-PA 6 (20 kV) and (f) PEI-PA 6 (30 kV). Inset of (b) shows the optical image of the spider-web morphology. (g) Dynamic response of QCM sensors coated with three different sensing structures upon exposure to increased formaldehyde concentrations. The inset is the amplified image for 1 ppm formaldehyde detection; (h) dependence of frequency shift for QCM-based PEI-PA 6 NFN (30 kV) sensors with various PA 6 coating loadings on formaldehyde concentration (1–100 ppm). Reproduced with permission from [258], copyright 2011 Royal Society of Chemistry.

A polyethyleneimine (PEI) functionalized polyamide 6 (PA 6) nanofibrous net (NFN) (PEI-PA 6 NFN) has been evaluated for formaldehyde sensing using a QCM platform [258]. The NFN structure contains a nanofibrous web as shown in Figure 19b (inset). This NFN membrane shows advantages of large specific surface area, high porosity, large stacking density and strongly tight adhesive force to the devices. These advantages result in ready facilitation of analyte diffusion and oscillation transmission into the membranes. Figure 19a–f shows SEM images of a 2D spider-web like nano-net comprising interlinked ultathin nanowires with a diameter of 26 nm. These 1D nanowires are supported by conventional electrospun PA 6 fibers [258]. A response of 1.4 Hz toward 1 ppm formaldehyde has been measured for PEI flat film based QCM sensors. These sensors show maximum frequency shifts of 3.8, 5.7, 9.7, 13.9 and 19.0 Hz upon exposure to 5, 15, 35, 70 and 100 ppm fomaldehyde, respectively. However, in comparison a maximum frequency shift of 2.4 and 4 Hz has been measured for the PEI-PZ 6 NFN (20 and 30 kV), respectively under the same conditions. As shown in Figure 19g, maximum responses of 19.0, 25.6 and 52.8 Hz toward 100 ppm formaldehyde for QCM sensors coated with PEI flat film, PEI-PA 6 NFN (20 kV) and PEI-PA 6 NFN (30 kV) have been measured [258]. Similarly, PANI functionalized PA6 nanofibers were used for HCl gas detection using QCM with a detection limit of 7 ppb at RT [259].

**4.3.2 Conducting polymer:** Polyacrylic acid (PAA) has proven to be one of the best polyelectrolyte sensing materials for ammonia and trimethyleamine (TMA) because of the interaction between analyte molecules and the carboxyl groups of PAA [260–261]. The PAA fibrous membrane (FM) morphologies for different ratios of water/ethanol (100/0, 50/50, and 0/100) result in an average diameter of 1.1 µm, 6.7 µm, and 2 µm, respectively. A PAA fibrous membrane formed with water as the solvent shows the highest response (232) to 1 ppm of ammonia compared with those obtained from the mixed solvent and pure ethanol [260]. A low detection limit up to 130 ppb for ammonia at a relative humidity of 40% is measured. The maximum frequency shift of PAA-QCM sensors is 113, 60, and 94 Hz when formed from H_2_O/ethanol with weight ratios of 100/0, 50/50, and 0/100, respectively. The maximum frequency shift of the PAA-QCM sensors exposed to 130 ppb, 450 ppb, 1 ppm, 2 ppm, and 5 ppm of ammonia is 12, 37, 60, 111, and 232 Hz, respectively. The maximum frequency shift of the PAA-QCM sensors increases with increase in the coating load. Sensors with higher coating loadings show a higher response for the same concentration of ammonia. The effect of humidity is linear for the PAA-QCM sensors. The frequency shifts increase from 32 to 225 Hz with increased relative humidity from 25% to 45%, because H_2_O molecules are pre-sorbed by the high proportion of hydrophilic carboxyl groups of PAA in the fibrous membranes [260]. The morphology of the PAA nanofibrous membrane has been changed to nanonet (nanofibers interconnected with a web like nanosheet) by adding a certain amount of NaCl into PAA, which further increases the surface area as well as the sensing properties [261].

Poly(styrene-*block*-maleic acid) (PS-*b*-PMA) NFs have been used as a novel sensing layer for QCM-based ammonia sensors [262]. PS-*b*-PMA, a block copolymer, is an ideal sensing material due to the interaction between ammonia molecules and carboxyl groups of PS-*b*-PMA. Different specific surface areas of PS-*b*-PMA are synthesized and named as samples A–F with respect to the mixture weight ratio of acetone/DMF at 0/10, 1/9, 1/4, 1/3, 3/7 and 5/5, respectively. The fiber diameters are broadly distributed in the range of 261–744 nm, although the majority are distributed in the 364–485 nm range. The specific surface area is varied by controlling the acetone concentration in a mixture of acetone and DMF. The specific surface areas for samples A–F are 1.9, 2.7, 3.7, 4.6, 3.6 and 0.9 m^2^/g, respectively [262]. The frequency shifts of the PS-*b*-PMA nanofibrous films exposed to 1.5, 2.5, 5, 10, 25 and 50 ppm of ammonia are 0.1, 0.6, 1.2, 1.5, 6.5 and 28.2 Hz, respectively. The frequency shifts of all sensors show good linear change with increased ammonia concentration. The developed sensors also show good reversibility by drying with nitrogen. In addition, the sensor shows similar frequency shifts with repeated injection of the same ammonia concentration in three experiments [262].

Jia et al. [263] report on the use of phenyl acetic acid (PA)-modified polystyrene (PS) nanofibrous membranes as the sensing material for the detection of ammonia in a QCM-based gas sensor. The sensing layer is prepared by PS NFs and later PA was dispersed on the surface of the NFs. The average diameter of the PS and PA modified nanofibers are 474 nm and 488 nm, respectively. The frequency shifts in the PS/PA-coated QCM sensors exposed to 1.5, 5, 10, 25 and 50 ppm of ammonia are 0.5, 1.1, 1.2, 2.1 and 0.4 Hz, respectively. The developed sensor detects concentrations as low as 1.5 ppm of NH_3_. The response shows a decline for increased ammonia concentrations [263].

**4.3.3 Conducting/Non-Conducting Polymer Blend:** A poly(acrylic acid) (PAA) and poly(vinyl alcohol) (PVA) blend has been used as a sensing layer with a QCM sensing platform for NH_3_ detection [264]. In this blend, PVA – which is water soluble – is used as a template, thus a strong bond between the PAA and water facilitates fiber formation and water evaporation. The viscosity of solutions increases with an increase in the weight percentage of PAA to PVA due to hydrogen bonding between carboxyl groups of PAA and hydroxyl groups of PVA. The conductivity of blend solutions is also increased from 74.9 to 102.2 mS/m with increased weight percentage of PAA to PVA from 11 to 33 wt % [264]. The average NF diameter increase from 200 to 330 nm with increased weight percentage of PAA to PVA from 0 to 33 wt %. The PVA nanofibrous membrane remains insensitive to NH_3_ whereas 11, 18, 25, and 33 wt % of PAA to PVA blended nanofibrous membranes show average resonance frequency shifts of 40, 150, 240, and 380 Hz, respectively. The frequency shift increases with an increase in PAA concentration into the blend. The average frequency shift for 50, 100 and 200 ppm is 150, 410, and 730 Hz, respectively. The frequency shift increases from 12 to 46 Hz with increased relative humidity from 50 to 60%. The frequency shift of 18 wt % PAA to PVA at the relative humidity of 50, 55, and 60% is 65, 150, and 330 Hz when exposed to 50 ppm of NH_3_ [264–265]. Electrospun nanofibers synthesised by blending polyvinylamine (PVAm) and polyacrylonitrile (PAN) has been deposited on QCM and utilized for formaldehyde sensing [266]. The developed sensor showed an extremely low detection limit of 500 ppb with rapid response time of 120 s. The developed sensor showed maximum a frequency shift of 0.2, 0.4, 0.7, 2.5, 4.2 Hz upon exposure to 0.5, 1, 5, 15, 35 ppm of formaldehyde, respectively [266].

**Figure 20:**
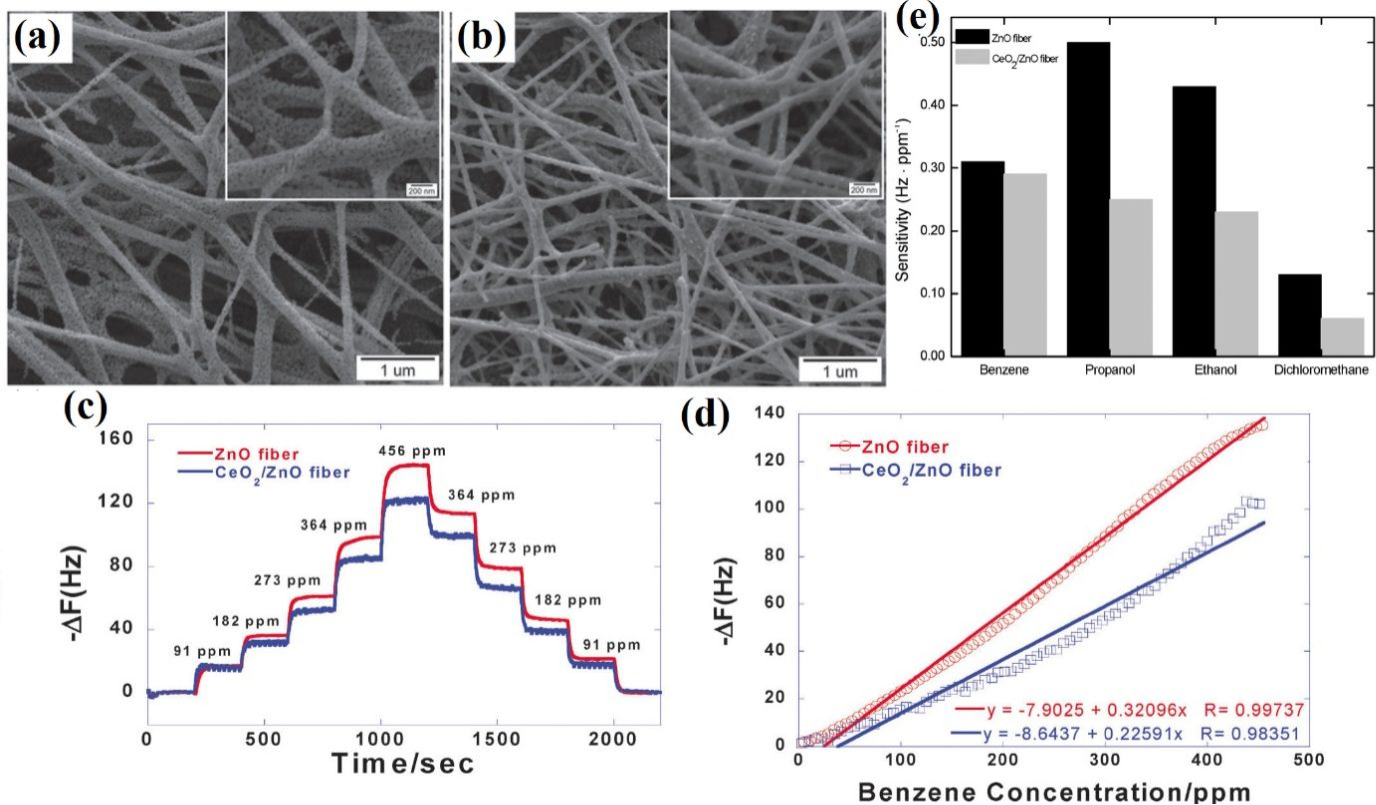
SEM images of (a) ZnO and (b) CeO_2_/ZnO nanofibers; (c) The step response for adsorption and desorption of benzene with ZnO and CeO_2_/ZnO fiber coated QCMs. (d) The linear adsorption response of ZnO and CeO_2_/ZnO fibers as a function of benzene concentration. (e) Sensitivity of the ZnO fiber and CeO_2_/ZnO-coated sensor to benzene, propanol, ethanol and dichloromethane vapours. Reproduced with permission from[267], copyright 2014 Royal Society of Chemistry.

**4.3.4 Semiconducting metal oxide:** A nanostructured complex of polyethyleneimine (PEI)-functionalized TiO_2_ NFs (PEI–TiO_2_) has been used as a sensing layer on a QCM for formaldehyde detection [21]. The developed sensor shows a high sensitivity of 13.7 toward 100 ppm of formaldehyde and a low detection limit of 1 ppm of formaldehyde at room temperature. The NFs have a porous structure with an average diameter of 625 nm. The highly porous structure of the fibers may be due to rapid phase separation during the calcination process. TiO_2_ ﬁbre morphologies change after dispersion with ethylene glycol (EG). TiO_2_ nanoparticles are transferred to the surface of fibers, perhaps during the magnetic stirring, thus leading to the hollow structure of these ﬁbres. The QCM sensor, coated with only TiO_2_ fibers, exhibits a frequency shift of only 0.2 Hz upon exposure to 1 ppm formaldehyde vapour. The frequency shift of the TiO_2_ fiber-coated QCM sensors exposed to 5, 15, 35, 70 and 100 ppm of formaldehyde is 0.3, 0.5, 0.9, 1.2 and 1.6 Hz, respectively. On the other hand, frequency shifts of 0.4 and 0.8 Hz toward 1 ppm formaldehyde vapour were observed for a PEI coating at loadings of 2700 and 6600 Hz. The PEI coating load of 6600 Hz shows a maximum response of 13.7 Hz to 100 ppm formaldehyde, which is eight times more than the response for the sensor based on TiO_2_ fibers [21]. Similarly, electrospun ZnO and CeO_2_/ZnO NF has been deposited directly on the QCM electrodes and been annealed in air at 500 °C for 5 hours. The developed QCM sensors were applied for volatile organic compound (VOC) sensing (such as benzene, propanol, ethanol and dichloromethane) [267]. The average diameter before annealing was 315 ± 95 and 270 ± 70 nm, which reduced to 160 ± 55 nm for ZnO and 110 ± 30 nm for CeO_2_/ZnO fibers, respectively (Figure 20a,b). The dynamic response of the sensors is shown in Figure 20c, where the maximum frequency shift is 116 Hz and 147 Hz toward 456 ppm of benzene vapors for CeO_2_/ZnO and ZnO fibers, respectively (Figure 20d). On the other hand, the sensor shows a poor selectivity toward benzene in an interfering environment of propanol, dichloromethane and ethanol (Figure 20e) [267].

**Figure 21:**
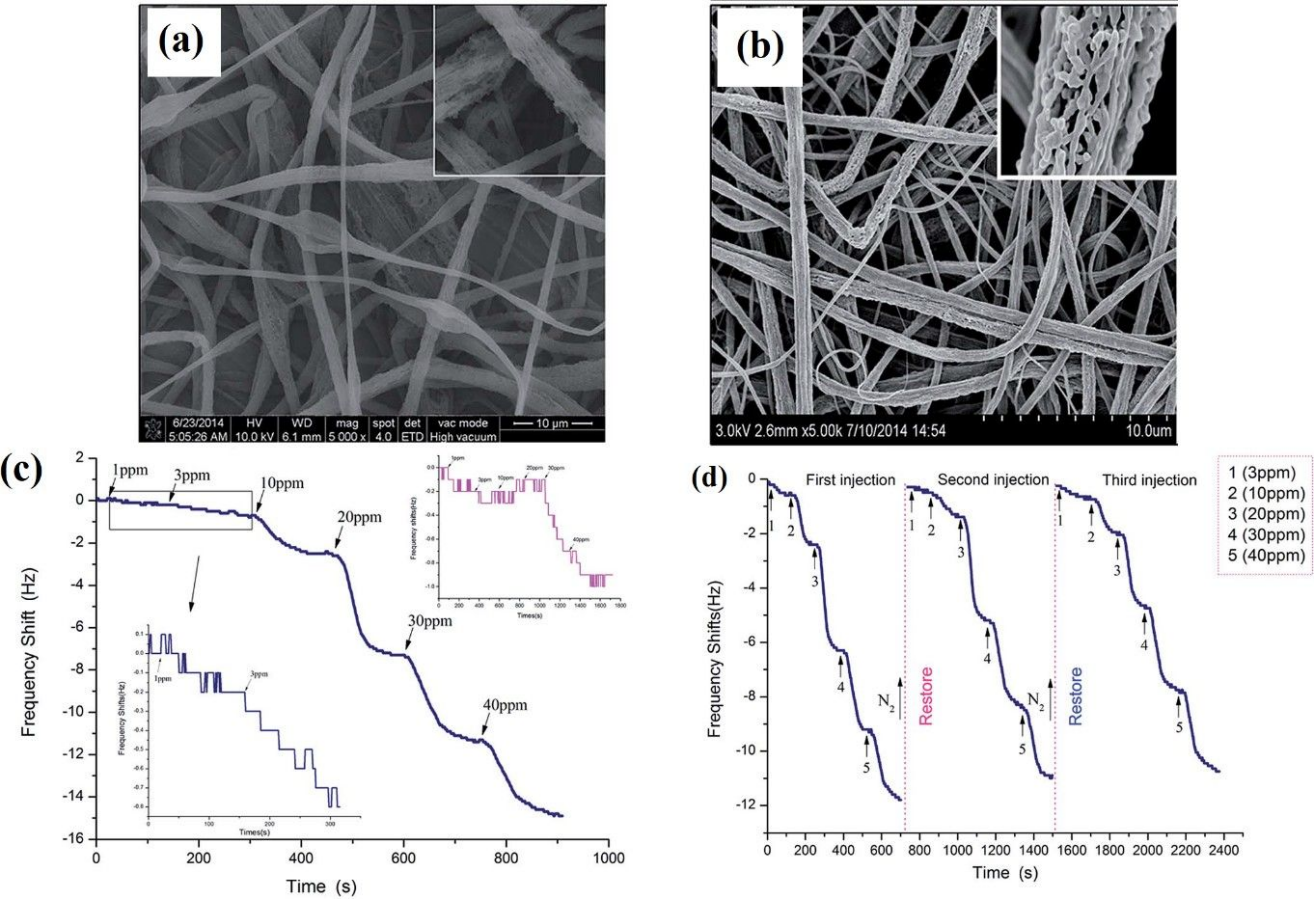
FE-SEM images of electrospun (a) pure porous PS NFs and (b) G-COOH/PS composite NFs. (c) Dynamic response of G-COOH/PS NFs to NH_3_; inset shows the response of PS NFs. (d) Reversibility testing for QCM sensors coated with G-COOH/PS membranes upon exposure to increased ammonia concentrations. Reproduced with permission from [268], copyright 2015 Royal Society of Chemistry.

**4.3.5 Graphene–conducting/non-conducting polymer composite:** Nanostructured complexes based on carboxyl graphene oxide (G-COOH) and polystyrene (PS) NFs have been utilized for ammonia detection based on a QCM platform [268]. The G-COOH/PS NFs (average diameter of 569 nm) consist of nanowires of diameter 37 nm. SEM images of the G-COOH/PS composite NFs indicate a mesoporous structure and that G-COOH sheets are randomly distributed within NFs (Figure 21a,b). The G-COOH shows a selective interaction between ammonia and the carboxyl group. The pure PS fibers show a beads-on-string morphology with an average diameter of 1 µm, whereas G-COOH/PS composite NFs show a random fiber morphology with an average diameter of 569 nm. Both types of NFs show nanowires with an average diameter of 37 nm. The diameter of the G-COOH/PS composite NFs is less than that of the pure PS NFs, which may be due to an increase in electrical conductance of the composite solution after addition of G-COOH [268].

The pure PS nanofibrous membrane shows an insignificant response for all concentrations less than 30 ppm NH_3_ with a maximum frequency shift of 0.5 and 0.3 Hz at 30 and 40 ppm, respectively. The frequency shift of the G-COOH/PS nanofibrous membrane coated QCM sensor exposed to 1, 3, 10, 20, 30 and 40 ppm of ammonia is 0.3, 0.5, 1.8, 4.8, 4 and 3.5 Hz, respectively (Figure 21c). A reversibility test was performed using N_2_ gas inserted after the sensor attained saturated. By exposing the sensor to repeated adsorption–desorption cycles, good reversibility of the as-prepared sensor was observed (Figure 21d) [268].

#### 4.4 Optical gas sensors

Recently, optical sensors have attracted a great deal of interest due to their exceptional physical property and mechanical advantages. An optical transducer is a new type of platform for sensing which is known to have better gas selectivity compared to other types of sensors [269]. Furthermore, optical sensors show immunity to electromagnetic interference (EMI) which allows for a wide range of applications not possible with other sensors. In addition, optical sensors are resistant to corrosive, reactive and flammable environments [270]. These sensors are able to integrate with existing fiber networks for remote and distributed sensing capabilities. Different spectrophotometric techniques have been developed, the most common being absorbance/transmittance/reflectance, Raman, Fourier-transform infrared spectroscopy (FTIR) spectroscopy and surface plasmon resonance (SPR).

PAN/ZnO composite nanofibers have been synthesized by combining atomic layer deposition (ALD) with electrospinning and applied to optical gas sensing of VOCs [271]. The ZnO layer is deposited on PAN nanofibers using ALD. The gas sensing characteristics are measured by photoluminescence (PL) spectroscopy at room temperature using a solid-state laser with wavelength 355 nm. The ZnO NFs are obtained with 5 min deposition time for electrospinning and coated with 50 cycles of ALD ZnO at 100 °C. The thickness of the ZnO coating at 20 nm was confirmed by TEM. The ZnO/PAN NFs show two emission bands, i.e., near-band emission (NBE) and deep-level emission (DLE). ZnO/PAN NFs exhibit a change in PL in an ethanol environment. The introduction of ethanol increases the NBE and decreases the DLE. The ratio of the NBE peak intensity and DLE before and after ethanol exposure is 0.83 ± 0.04 and 1.31 ± 0.03 toward 150 ppm of ethanol at room temperature, respectively [271].

Electrospun composite NFs of PAN containing metal oxide nanoparticles (Fe_2_O_3_, ZnO) (10% Sb_3_O_4_, 90% SnO_2_) have been synthesized and applied to CO_2_ sensing using FTIR spectroscopy [272]. The average diameter of pure PAN NFs reduced from 200 nm to 50–150 nm for composite NFs while the porosity increased from 70% for pure PAN NFs to 86% for composite counterparts. The absorption spectrum in air without composite NFs was lower than spectra for composite NFs. The PAN/Fe_2_O_3_ shows the highest absorption peak to 2000 ppm CO_2_ (C=H at 2356 cm^−1^) compared with the PAN/Sb-SnO_2_ [26,272].

**Figure 22:**
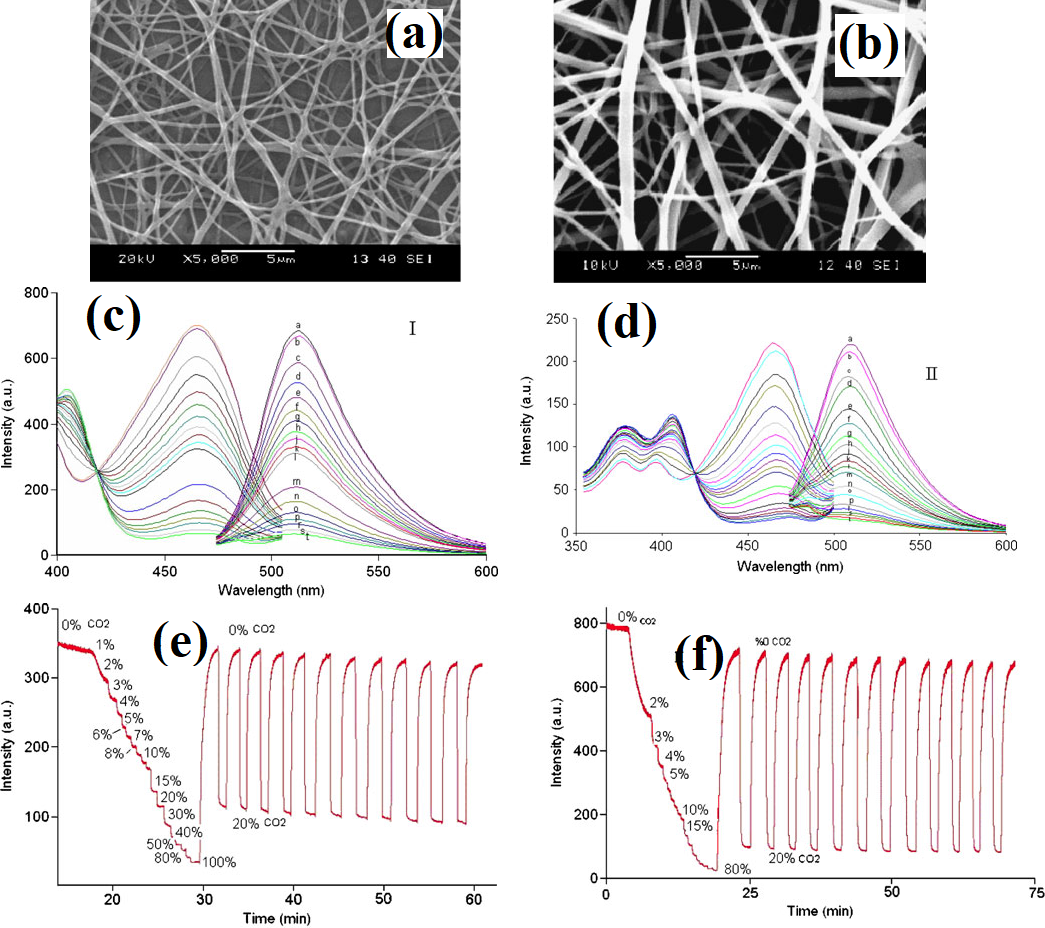
SEM images of electrospun nanofibrous membranes of (a) PMMA and (b) ethyl cellulose (EC). Excitation and emission spectra of electrospun fiber ion pair form of HPTS in (c) EC (d) PMMA after exposure to certain concentrations of CO_2_ (λ_max_(ex)= 465 nm, λ_max_(em) = 513 nm) – a: initial, b: 1% CO_2_, c: 2%, d: 3%, e: 4%, f: 5%, g: 6%, h: 7%, i: 8%, j: 8%, k: 9%, l: 10%, m: 20%, n: 30%, o: 40%, p: 50%, r: 60%, s: 80%, and t: 100% CO_2_. Emission-based kinetic response of ion pair form of HPTS in (e) EC and (f) PMMA to gaseous CO_2_. Reproduced with permission from [273], copyright 2010 Springer.

A fluorescence sensor has been developed for CO_2_ detection by using ion pair form of 8-hydroxypyrene-1,3,6-trisulfonic acid (HPTS) as a sensing material. Poly(methyl methacrylate) (PMMA) and ethyl cellulose (EC) are used as polymeric material whereas EC and PMMA have an average diameter in the range 370–527 nm (Figure 22a,b). The change in fluorescence spectra of electrospun materials as a function of different concentrations of CO_2_ is shown in Figure 22c,d. The fluorescence intensity decays with increasing CO_2_ concentration. The gaseous carbon dioxide converts into carbonic acid by reacting with water in nanofibers that interacts with the HPTS (fluorescent dye). The dynamic sensing results of both matrix materials are shown in Figure 22e,f for various concentrations of CO_2_. The response and recovery time varies between 1–5 min for both matrix materials [273].

Electrospun fibers of a fluorescent conjugated polymer (**P**) (benzothiophene-based conjugated polymer with sulfur-containing fused rings as the backbone) has been applied as a sensing layer for detection of explosives, including picric acid (PA), trinitrotoluene (TNT), 2,4-dinitrotoluene (DNT), and nitrobenzene (NB) [2–3]. Polystyrene is applied as a supporting material and is doped with conjugate polymer **P** during electrospinning. The nanofibers have an average diameter in the range of 800–1000 nm. Fluorescence microscopy images of electrospun nanofibers before and after exposing nanofibers to TNT vapours show the fluorescence is dramatically quenched. The conjugate polymer **P** exhibits maximum florescence intensity at 4.1 × 10^−7^ M concentration of TNT. The fluorescence intensity decreases with an increase in explosive concentration. The order of fluorescence quenching efficiency is PA > TNT > DNT > NB with values of 85%, 65%, 25% and 12%. The order of efficiency follows the same sequence as the electron deficiency of these explosives. The electrospun nanofibers exhibit different sensing efficiencies for different explosives that is related to their vapour pressure and electron deficiency. The conjugate polymer **P** nanofibers show the highest quenching efficiency to NB at around 4 times that of DNT [2–3].

**Figure 23:**
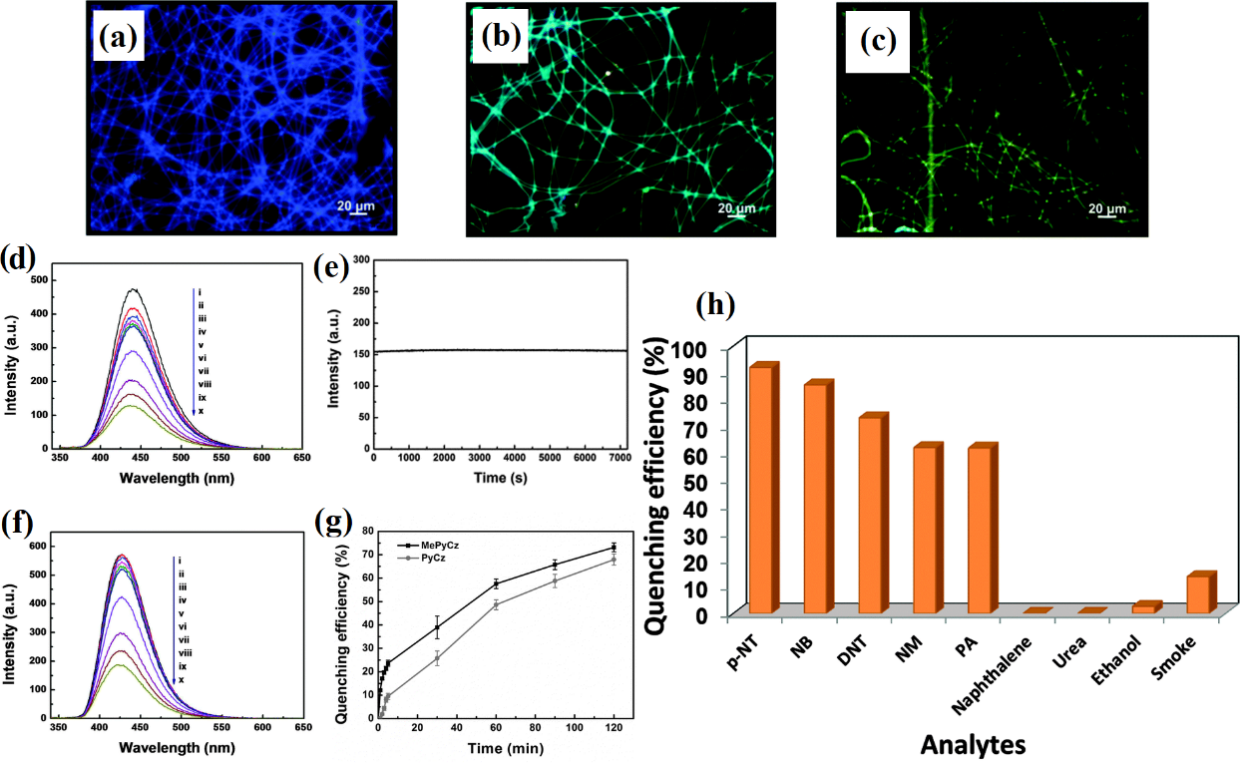
FLM images of (a) PEO/MePyCz/ThFO (*V*_MePyCz_:*V*_ThFO_ 16:1), (b) PEO/MePyCz/ThFO (*V*_MePyCz_:*V*_ThFO_ 4:1) and (c) PEO/ThFO hybrid nanofibers. (d) Time-dependent fluorescence quenching process of PEO/MePyCz fibrous film toward DNT vapour (from i to x: 0, 1, 2, 3, 4, 5, 30, 60, 90, 120 min; λ_ex_ = 330 nm). (e) Time course curve of PEO/MePyCz hybrid fibrous film in ambient air without DNT. (f) Time-dependent fluorescence quenching process of PEO/PyCz fibrous film toward DNT in vapour (from i to x: 0, 1, 2, 3, 4, 5, 30, 60, 90, 120 min; λ_ex_ = 330 nm). (g) The quenching efficiency curves versus exposure time with standard deviation error bars of three batches prepared at different times. (h) Fluorescence quenching efficiencies of PEO/MePyCz electrospun nanofibrous films toward various analytes. Reproduced from [274], copyright 2015 Royal Society of Chemistry.

Fluorescent polymer nanofibers are used for the chemical sensing of explosives by electrospinning fluorescent polymer or by doping low cost polymers with fluorophores [274]. Xue et al. [274] has synthesized composite nanofibers based on PEO/MePyCz (polyethylene oxide/4-(2-(2-(2-methoxyethoxy)ethoxy)ethoxy)-9-(pyren-1-yl)-9*H*-carbazole) for nitro-compound colorimetric sensing. The PEO/MePyCz NFs have an average diameter in the range of 0.8 µm to 1.6 µm. Moreover, fluorescence microscopy images (FLM) of the fiber show evenly distributed blue-emitting sensing molecules of MePyCz in PEO nanofibers. The PEO/MePyCz composite nanofibers are further functionalized by ThFO (2-(thiophen-2-yl)-fluoren-9-one) to prepare PEO/MePyCz/ThFO composite colorimetric sensors, which change the emission from blue to cyan to green (Figure 23a–c). The fluorescence spectra of PEO/MePyCz composite nanofibers toward DNT vapour is shown in Figure 23d–g. The quenching efficiency of the PEO/MePyCz composite nanofibers reached to 89% with the 0.5 wt % doping of MePyCz with no decay in ambient environment. The spin-coated film of PEO/MePyCz shows an efficiency of 73%. The selectivity of PEO/MePyCz has also been tested against various nitro-compounds as shown in Figure 23h. The PEO/MePyCz composite nanofibers do not show any quenching toward urea, ethanol and naphthalene [274].

Evanescent optical fiber humidity sensors have been fabricated using PAA nanofibers and tested for 30% to 95% RH [275]. The PAA nanofibers are deposited on the optical core of the fiber optic cord. The samples synthesized with PAA solution with viscosity 47 and 49 cps are named as A and B. Samples made with different deposition times (which corresponds to different densities) are labelled as sample 1 (5 min), sample 2 (15 min), and sample 3 (30 min). The average fiber diameter for samples 1A, 2A and 3A is 1.61 ± 0.28 µm, 1.54 ± 0.32 µm, and 1.51 ± 0.43 µm, respectively. In contrast, the average fiber diameter for samples 1B, 2B and 3B is 0.59 ± 0.15 µm, 0.48 ± 0.09 µm and 0.53 ± 0.18 µm, respectively. The sensing characteristics of these optical fiber sensors depend dramatically on the electrospun nanofiber diameter and density of the electrospun nanofiber mat. The relative absorption spectrum of the sensor 1B shows that the absorption decreases with an increase in RH. The effect means that higher power is transmitted with higher relative humidity, resulting in lower absorption values. On the other hand, the 1A sensor with a lower density of nanofibers shows the opposite response to that of sample 1B. Losses from these electrospun nanofibers results in a decrease in optical transmittance. Highly dense nanofibers (3A and 3B) show slow response and high levels of hysteresis. The best results are obtained from the 1B samples that have a thinner diameter and high fiber density. The response time for these samples is 340 ms, with a recovery time of 210 ms [275].

**Figure 24:**
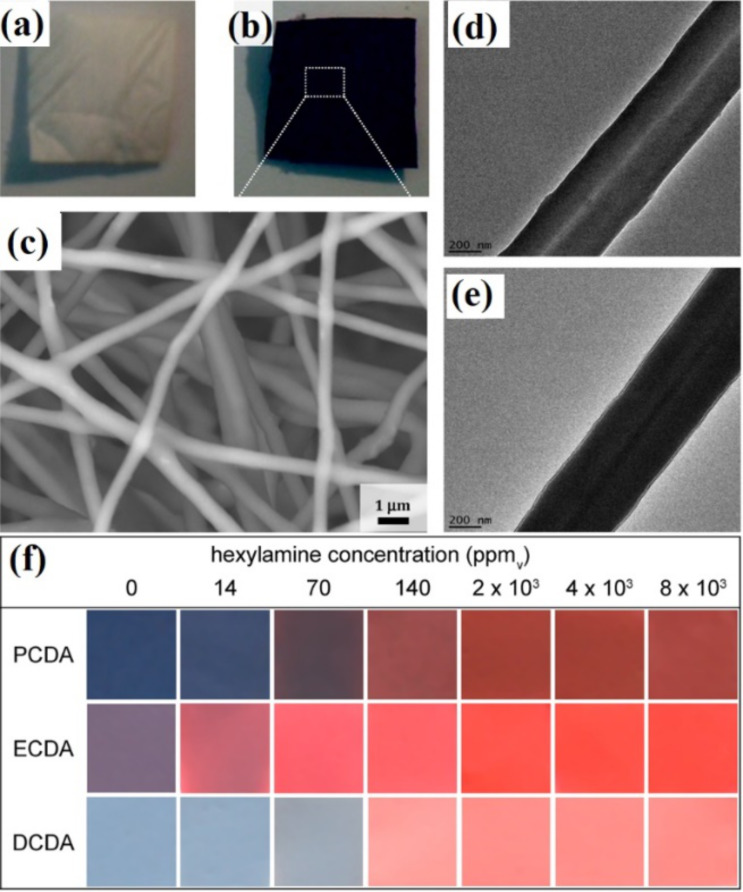
Photographs of electrospun fiber mat embedded with PCDA–biotin before (a) and after (b) UV-irradiation (1 mW cm^−1^) for 5 min. (c) SEM image of the nanofibers containing polymerized PCDA–biotin. (d, e) TEM images of PCDA–biotin nanofibers before and after UV-irradiation. (f) Flatbed scanner images illustrating the colorimetric response of a polydiacetylene-embedded nanofiber sensor array to various concentrations of hexylamine vapour. Reproduced from [276], copyright 2014 ACS.

Davis et al. [276] have developed a dual-mode optical sensor with electrospun nanofibers embedded with various polydiacetylene (PDAs). The four most common solvents have been identified using solvent-dependent fluorescent transition of nanofibers. Moreover, biomolecules has also been detected by biotinylated-PCDA monomers embedded into silica reinforced nanofiber mats. The nanofibrous mats are produced with a uniform fiber distribution of 400–600 nm for fluorescence experiments. PDA nanofibers show excellent fluorescence detection with a distinct fluorescence transition from blue to red against various organic solvents (hexane, methanol, chloroform, and THF). The optical and SEM images of nanofibers encapsulated with PCDA–biotin are shown in Figure 24a–c. The fibrous mat converts into blue phase under UV irradiation. The TEM images of the PEO/TEOS electrospun nanofibers mats encapsulated with PCDA–biotin, before and after UV-irradiation, shows no morphological change (Figure 24d,e). Volatile amine groups are identified using three-component PDA nanofibers with an exposure time of 30 min and results are shown in Figure 24f [276].

Polydimethylsiloxane (PDMS) containing polycaprolactone (PCL) core–shell nanofibers have been synthesized by electrospinning and used as an optical oxygen sensor [277]. The nanofibers consist of two parts PDMS containing 0.1% ruthenium(II) dichloride (Ru(dpp)(Cl)), moisture-cured PDMS containing 0.5% weight doping ratio of ruthenium(II) tetraphenylboron (Ru(dpp)(PB)), and moisture-cured PDMS containing 0.5% weight doping ratio platinum (II) octaethylporphyrin (PtOEP), labelled as S1, S2, and S3. The core–shell nanofibers of S1, S2, and S3 have average diameters of 512 ± 195, 401 ± 131, and 570 ± 192 nm, respectively. The S1, S2, and S3 core–shell fibers absorb visible light at 455 nm and emit red luminescence at 618, 622, and 645 nm, respectively. The intensity of the emission is quenched with an increase in oxygen concentration without any peak shift. The sensitivity of the sensors is represented by the ratio of the intensity of an oxygen-free environment to the intensity of pure oxygen (*I*_0_/*I*_100_). The *I*_0_/*I*_100_ ratios for S1–S3 are 2.97, 4.1, and 24, respectively. The response times of samples S1–S3 are 0.36 ± 0.05 s, 0.28 ± 0.08 s, and 0.49 ± 0.13 s, respectively. In addition, the recovery times are 0.72 ± 0.27 s, 0.51 ± 0.15 s, and 0.70 ± 0.15 s, respectively [277].

Electrospun nanofibers based on analyte-sensitive dyes immobilized in polymer have been used as a sensing material in optochemical (luminescence) sensors for oxygen detection [278–279]. Luminescence lifetime was measured by phase-shift fluorometry with a bluish-green light as an excitation source. Electrospun nanofibers are synthesized by mixing an oxygen-sensitive dye, platinum tetra(pentafluorophenyl) porphyrine (PtTFPP), with polystyrene. The fibers have a band-like morphology with a width of 620 nm and thickness of 100 nm at the thinnest part of the fiber. Moreover, the fibers are nanoporous on the surface which increases the diffusion of the analyte gas. The nanofibers are excited with a wavelength of 505 nm. The electrospun film shows slightly enhanced sensitivity compared with a thick film. The electrospun film shows almost a two times faster response time (2.2 s) compared with that of the thick film (4 s). One drawback with electrospun films is the degradation of a polymer dye due to enhanced accessibility of oxygen; this is commonly known as photo bleaching. With this approach, photo bleaching may be controlled by increasing the thickness of the electrospun film to a moderate range [278–279].

### Future Perspectives and Conclusion

In summary, we have reviewed recent progress and achievements in the rapidly emerging field of gas sensing using electrospun 1D nanostructures. The dominant 1D nanostructures used to date in this field include nanofibers, nanowires, hollow nanofibers and nanotubes although other forms are known. The chemical, electrical, optical and gravimetric response to different target gases are compiled for many materials, including pure semiconducting metal oxides (MOx), MOx functionalized by noble/rare-earth/transition metals, MOx–MOx composites, conjugate polymer–MOx and graphene–MOx composites. In addition, these responses are, to a large degree, dependent on many different fibrous assemblies that are possible, including mixed nanocomposites, double-layer and core–shell structures and hollow forms. A wide range of analytes such as NO_2_, NH_3_, CO, CO_2_, H_2_, H_2_S and volatile organic compounds (e.g., ethanol, toluene, acetone and formaldehyde) can be detected using electrospun 1D nanostructures, with a detection limit as low as 7 ppb and response/recovery times as quick as milliseconds.

Recent advances in the technology, as well as the volume and diversity of outcomes, clearly demonstrate that the electrospinning method offers unique opportunities for development of highly sensitive and selective 1D nanostructures with remarkable specific surface area and high porositity. Electrospun fibers show versatility of function via appropriate integration with specific sensing architectures for particular target outcomes. For example, the design of a particular sensor may be guided by the advantages and disadvantages listed in Table 1 that refers to fiber structure. Table 1 highlights the generic trade-offs between sensitivity, response time and detection limit using 1D nanostructures.

**Table 1:** Advantages and disadvantages of 1D nanostructures.

Structure	Advantage	Disadvantage	References

nanofibers	short recovery time	minimal response, long response time	[95,110,115,144,280–281]
hollow nanofibers/nanotubes	high sensitivity, short response time	long recovery time	[78,102,119,129,133,282]
nanowires	high sensitivity, low detection limit	long response/recovery time	[135]

For 1D materials, we find that gas sensing performance can be improved in two ways:

(i) By enhancing the specific surface area of nanofibers via the synthesis of different morphologies such as micro- or mesoporous fibers, nanobelts, hierarchical nanofibers, and core–shell nanofibers. Such structures can be obtained by using electrospinning alone or in combination with sputtering or chemical vapour deposition [37,69–76,78,80]. The surface reactivity of a chemisorbed oxygen and target gas is increased by introducing hollow nanofibers or nanotubes because a target gas can react with both the inner and the outer walls of the hollow nanofiber or nanotube. On the other hand, with hollow fibers the recovery time increases because a target gas takes more time to desorb completely from the inner walls. Thus, there is a trade-off between sensitivity and recovery time of hollow nanofiber based gas sensors. Moreover, the surface area cannot be increased indefinitely to improve the sensitivity of nanofibers because there is an optimum surface area for which a nanofibrous gas sensor shows maximum sensitivity, regardless of morphology [79,83,132–133,139,141].

(ii) By functionalizing nanofibers with different catalytic materials such as noble metals, rare-earth metals, transition metals and graphene and/or by making composites of two or more materials. Functionalization of electrospun fibers with other materials such as noble metals, metal oxides and carbon materials remarkably improves the sensing performance in terms of sensitivity, selectivity, response and recovery rate by improving charge transport properties and by enabling more surface active sites. In some cases, the effect of crystal facets with high surface reactivity [12–13] significantly influences the sensing performance. Table 2 summarizes the specific characteristics of nanofibrous gas sensors using a range of different materials.

**Table 2:** Advantages and disadvantages of functionalized materials. Note that the response of pure metal oxide nanofibers (Table S2 in Supporting Information File 1) is taken as the reference point.

Material	Advantages	Disadvantages	References

pure MOx	minimal response/recovery time	minimal sensitivity, high operating temperature, low detection limit	[100,105,115,118] [119,144,282–284]
functionalized MOx	high sensitivity, lower detection limit, short response/recovery time	slightly lower operating temperature	[10–11,104,175,178–179] [188,200,209,215,220]
MOx–MOx composites	enhanced selectivity, short response/recovery time, improved operating temperature	slightly improved response, slightly improved detection limit	[222,224–228,230] [235,285–287]
conjugated polymer–conjugated polymer composites or conjugated polymer/non-conjugated polymer–MOx composites	low operating temperature, high selectivity, minimal detection limit, minimal response/recovery time	minimal response	[87,238,241,248,258] [278–279,288–294]
graphene–MOx, CNTs–MOx	lower operating temperature, minimal response/recovery time, minimal detection limit,	minimal response	[89,243–244,246,252] [268,295–296]

Recently, a great deal of attention and research effort has been devoted to the development of electrospun fibers incorporated with functional nanoparticles (NPs) [39–40]. This approach shows good potential for applications involving the self-assembly of anisotropic NPs to generate new functional devices [63]. These materials could be beneficial for gas sensing applications utilizing the properties of both nanostructures and polymers. These hybrid materials need to be explored for all types of gas sensing platforms [57].

We have also outlined the differences between sensing technologies used with electrospun fibers. Table 3 summarizes the four predominant platforms used with gas sensors.

**Table 3:** Advantages and disadvantages of current gas sensing platforms.

Platform	Advantages	Disadvantages	References

conductometric & MEMS	high sensitivity, easily miniaturized	high power requirement, high operating temperature, high response/recovery time	[75,79,89,93,188,215,280,283,285,292,295]
surface acoustic wave (SAW)	low power requirement, room temperature operation	low sensitivity, high response/recovery time	[247–249,251–252]
quartz crystal microbalance (QCM)	low power requirement, room temperature operation, low detection limit	low selectivity, sensitive to humidity, high response/recovery time	[21,256,258,262–263]
optical	fast response time, low power requirement, high selectivity, room temperature operation	low sensitivity, higher detection limit	[2–3,271,275,297]

Many existing gas sensors based on electrospun 1D nanostructures are conductometric, due to their reduced cost, simple operation and potential for device miniaturization. Conductometric sensors are able to detect many different gases with high and fast response. However, for many of these sensors, an operating temperature above 100 °C is a requisite for efficient operation. This requirement, usually related to the effective recovery rate, may limit their extended use under ambient or mobile circumstances. Conductometric gas sensors developed in MEMS operate with much lower power consumption due to their miniaturized size [165,298]. For environmental sensing, as well as for some biomedical applications, it is desirable to deploy gas sensors that operate at room temperature or at least below 100 °C. Operation of gas sensors at these temperatures will result in lower power consumption with potential for associated reductions in weight and/or size. These two feature improvements may pave the way for mobile, multi-locale, selective gas sensors to provide statistically viable data on a wide range of environments. In addition, there is a much lower (or no) risk of ignition if the sensor is deployed to detect flammable or explosive analytes.

Gas sensing platforms other than conductometric, such as resonating or optical platforms offer room temperature operation with enhanced sensing performance. However, optical technology is relatively expensive to fabricate and to operate. Surface acoustic wave sensors operate at room temperature but have limited response and modest detection limits. The solution to this cost/performance problem may come from further development of QCM technology. QCM technology is simple, robust, low cost, and has been shown to produce excellent results even at room temperature [253–254]. For example, QCM technology has been demonstrated to show lower detection limits of 50 ppb and 130 ppb for detection of formaldehyde and NH_3_ gases, respectively [257–258,260].

QCM devices can detect changes in the mass of an adsorbed target gas in picogram quantities and are highly sensitive. However, QCM devices are also sensitive to a humid environments, and thus to date their applications are limited. Nevertheless, these devices have a very short response and recovery time (e.g., 3 s). The incorporation of electrospun 1D nanostructures in QCM devices that operate at room temperature and in combination with several sensors in a single platform is a promising avenue for research which has not yet been sufficiently explored. The potential for QCM devices to combine reproducibility, reliability and stability is an open field of investigation which may lead to excellent sensing systems in the near future. Furthermore, few metal oxides and their composites with other metal oxides, conjugate polymers and/or carbon compounds (e.g., graphene) on QCM sensing platforms have been explored. There is also a need to develop new materials for QCM gas sensors, which are insensitive to humidity, for real world applications.

It is evident from this review that there is a bright future for high-performance chemical sensors thanks to the unique properties of electrospun 1D nanostructures. Furthermore, there is a great opportunity for further development of materials, their functionality and their integration with an array of platforms to provide highly selective and sensitive data on a target gas in a multi-gas environment. The diversity of material compositions now available, in conjunction with novel additives and sophisticated nanostructures, provides a plethora of choices for the aspiring surface or gas phase scientist. The future challenge will be to align these choices with tractable problems that highlight the versatility and value of electrospun 1D nanostructures.

## Supporting Information

Different types of electrospun material based gas sensors. Sensing performance of electrospun pure MOx nanofibers categorized based on the analyte gas. Sensing performance of electrospun metal-doped MOx nanofibers categorized based on the analyte gas. Sensing performance of electrospun MOx–MOx nanofibers categorized based on the analyte gas.

File 1 Summary of electrospun materials and their gas sensing performance.
