# Supplementary material for: Electrospun one-dimensional nanostructures: a new horizon for gas sensing materials
Source: Beilstein J Nanotechnol. 2018 Aug 13;9:2128–70. doi: 10.3762/bjnano.9.202 (PMC6122236; doi:10.3762/bjnano.9.202)
Supplement: File 1 — Summary of electrospun materials and their gas sensing performance. [file Beilstein_J_Nanotechnol-09-2128-s001.pdf]

Supporting Information

for

Electrospun one-dimensional nanostructures: a new horizon for gas sensing materials

Muhammad Imran[1], Nunzio Motta[1], and Mahnaz Shafiei[1,2,]*

Address: [1]Institute for Future Environments and School of Chemistry, Physics, and Mechanical Engineering, Queensland University of Technology (QUT), Brisbane, QLD 4001, Australia and [2]Faculty of Science, Engineering and Technology, Swinburne University of Technology, Hawthorn, VIC 3122, Australia

Email: Mahnaz Shafiei - mshafiei@swin.edu.au

*Corresponding author

Summary of electrospun materials and their gas sensing performance

Table S1: Different types of electrospun material based gas sensors.

	Electrospinning parameters											Sensing Performance					
**Sensing Platform**	**Polymer**	**Solvent**	**Precursor**	**Needle Type**	**Flow Rate**	**Applied Voltage (KV)**	**Working Distance (cm)**	**Post-treatment**	**Material**	**Morphology**	**Diameter**	**Response**	**Optimum Operating Temperature**	**Detection Range**	**Response/Recovery Time**	**Analyte Gas**	**Ref.**
Conductometric	PVP	DMF	$In(NO_3)_3 \cdot xH_2O$	21-gauge needle	0.05 ml/h	20	22	600 °C for 2 h	CuO-In_2O_3 nanocomposite	NFs	40 nm	1.16×10^5/5ppm	150 °C	5 ppm	-/140s	H_2S	[1]
Conductometric	PVP	DMF:ethanol (1:1)	$In(NO_3)_3 \cdot 4.5H_2O$	hypodermic syringe	1.0 ml/h	20	25	600 °C for 1 h	In_2O_3–SnO_2 composite	NFs	40-100 nm	8.1/1ppm	80 °C	1-8.1 ppm	1s/6s	Trimethyl amine	[2]
Conductometric	PVP	Ethanol, DMF	WCl_6	23SP	1 µl/min	14	10	300 – 500 °C for 1 h	WO_3	NFs	20-100 nm	12.4/400 ppb	75 °C	400 ppb	30s/32s	NO_2	[3]
Conductometric	Poly(styrene-co-acrylonitrile)	DMSO	zinc acetate	hypodermic syringe ID = 0.5 mm	1.0 ml/h	20	17	At 500 °C, 700 °C and 900 °C for 2 h	ZnO	NFs	60 nm	6/40 ppm	160 °C and 200 °C	40 ppm	--	NH_3	[4]
Conductometric	PVP	DMF:ethanol (1:1)	P-type $La_{0.7}Sr_{0.3}FeO_3$ NPs	hypodermic syringe	1.0 ml/h	10	20	600 °C for 5 h in air	L_4SnO_2	NFs	20 nm	28/1 ppm	300 °C	1 ppm	9s/15s	C_2H_5OH	[5]
Conductometric	PVAc	DMF	Tin (IV) acetate	stainless steel needle 25 gauge	5 µl/min	16.5	15	500 °C for 2 h in air	SnO_2	NFs	210± 35 nm	57/2.5 ppm	300 °C	125 ppb-2.5 ppm	--	NO_2	[6]
												6.4/2.5 ppm	150 °C			H_2	
Conductometric	PVP	Ethanol, DMF	$Co(NO_3)_2 \cdot 6H_2O$ and rGO	N7-gauge needle	0.2 ml/h	18.5	13.5	800 °C for 30 min	rGO-Co_3O_4 composite	NFs	200-300 nm	53.6%/50 ppm	20 °C	5 -100 ppm	4s/5 min	NH_3	[7]
Conductom	PVA	DI water	$In(CH_3COO)_3$	--	--	20	15	400 °C	In_2O_3	NFs	150-	~540%/10	300 °C	100	--	CO	[8]

	Electrospinning parameters											Sensing Performance					
**Sensing Platform**	**Polymer**	**Solvent**	**Precursor**	**Needle Type**	**Flow Rate**	**Applied Voltage (KV)**	**Working Distance (cm)**	**Post-treatment**	**Material**	**Morphology**	**Diameter**	**Response**	**Optimum Operating Temperature**	**Detection Range**	**Response/Recovery Time**	**Analyte Gas**	**Ref.**
etric								, 500 °C or 600 °C in air for 3 h			200 nm	0 ppm		ppm			
Conductometric	PVP	Ethanol:DMF (1:3)	Indium nitrate ($In(NO_3)_3 \cdot xH_2O$),	stainless steel needle 21 G	1.5 ml/h	15	15	600 °C for 3 h in air	In_2O_3	NRbs	20-300 nm	~68.5/12ppm	200 °C	1 - 17 ppm	43min/93 min	NO_2	[9]
Conductometric	PVAc	DMF	Titanium (IV) propoxide	steel orifice (inner diameter of 450 µm	1.5 ml/h	1.5	10	three step 280 °C, 350 °C, and 420 °C each step 1 h	TiO_2	Porous nanostructured layer (nano-grains)	29-42 nm	~2/2 ppm CO ~3.25/2 ppm NO_2	450 °C	0.25 - 2 ppm	Several min	CO and NO_2	[10]
Conductometric	PVP	DMF:ethanol (1:1)	$In(NO_3)_3$ .4.5H_2O	--	--	20	25	600 °C for 4 h in air	$In_{2-x}Ni_xO_3$	NFs	62 nm	7.2/500 ppb 107.7/10 ppm	70 °C and 130 °C	0.5 - 20 ppm	580s/650s	NO_2	[11]
Conductometric	PVP	DMF:ethanol (1:1)	$In(NO_3)_3$ and $Mg(NO_3)_2 \cdot 6H_2O$	23 G	--	15	15	600°C for 2 h in air	Mg-In_2O_3	NTs	80 nm	173.7/10 ppm	150 °C	0.5 - 10 ppm	--	H_2S	[12]
Conductometric	PANI /PMMA	THF	--	--	--	7	6	-	PAN/PMMA Composite	NFs	2.5-3.5 µm	1.38/1 ppm	RT	1–30 ppm	10s/-	NH_3	[13]
Conductometric	PVP, PAN	DMF	$SnCl_2 \cdot 2H_2O$	--	0.4 ml/h	20	15	500 °C for 4 h in air	SnO_2	NFs	80-400 nm	2.4/1 ppm	150 °C	0.06%	21s/333s	H_2	[14]
Conductometric	PVA	DI water	$SnCl_4 \cdot 5H_2O$	--	--	10	10	300, 500	SnO_2	NFs	100 nm	4.5/10 ppm	330 °C	10 ppm	13s/13.9s	ethanol	[15]

	Electrospinning parameters											Sensing Performance					
Sensing Platform	Polymer	Solvent	Precursor	Needle Type	Flow Rate	Applied Voltage (KV)	Working Distance (cm)	Post-treatment	Material	Morphology	Diameter	Response	Optimum Operating Temperature	Detection Range	Response/Recovery Time	Analyte Gas	Ref.
								and 700 °C for 4 h									
Conductometric	PVP	DMF:ethanol (1:1)	$Zn(NO_3)_2$.$6H_2O$ and $SnCl_2$.$2H_2O$	--	--	--	--	600 °C for 5 h	ZnO – SnO_2	HFs	150 nm	83/20 ppm	260 ℃	200 ppm	4-7s/4-5s	ethanol	[16]
Conductometric	PVP	DMF	$In(NO)_3$ .4 ½ H_2O and $Ce(NO)_3$.$6H_2O$	--	--	16	--	600 °C for 3 h	In_2O_3 – CeO_2	NTs	OD 90-180 nm	498/20 ppm	80 ℃	20 ppm	64s/204s	H_2S	[17]
Conductometric	PVP	DMF	$In(NO)_3$ .4 ½ H_2O and $Al_2(NO)_3$	ID = 0.8 mm	0.25 ml/h	15	--	550 °C for 4 h in air	Al_2O_3 – In_2O_3	NTs	200 nm	0.47/291 ppb	RT	291 ppb-97 ppm	24s/--	NO_x	[18]
Conductometric	PVP	DMF:ethanol (1:1)	$In(NO_3)_3$ and $SnCl_2$	ID = 0.8 mm	0.004 ml/min	15	--	550 °C for 4 h in air	In_2O_3 – SnO_2	NRs	230 nm	8.98/100 ppm	RT	0.1-100 ppm	4.67s/--	NO_x	[19]
Conductometric	PVP	acetic acid and ethanol	$C_{16}H_{36}O_4Ti$ and $In(NO_3)_3.4.5H_2O$	0.8 mm	--	14	--	600 °C for 4 h in air	In_2O_3/TiO_2	NFs	200 nm	41.1%/97 ppm	RT	97 ppb – 97 ppm	3s/--	NO_x	[20]
Conductometric	PVP	DMF:ethanol (1:1)	$In(NO_3)_3$	--	--	15	--	500 °C for 2 h	In_2O_3	NTs<br>NWs	--	23.46/100 ppm<br>14.22/100 ppm	280 °C	100 ppm	1s/57s<br>1s/34s	acetone	[21]
Conductometric	PVP	DMF and ethanol	$Fe(NO_3)_3$. $9H_2O$ and $Al(NO_3)_3$. $9H_2O$	--	--	13	--	550 °C for 4 h	Al-doped α-Fe_2O_3	NTs	OD 90	41.8/50 ppm	240 °C	300 ppb – 500 ppm	20s/60s	ethanol	[22]
Conductometric	PVP	DMF:ethanol (1:1)	$SnCl_2·2H_2O$ and $AlCl_3·6H_2O$	--	--	10	15	600 °C for 2 h	Al-doped SnO_2	NTs	OD 200	7.82/1000 ppb	240 °C	1000 ppb – 100 ppb	--	formaldehyde	[23]

	Electrospinning parameters											Sensing Performance					
**Sensing Platform**	**Polymer**	**Solvent**	**Precursor**	**Needle Type**	**Flow Rate**	**Applied Voltage (KV)**	**Working Distance (cm)**	**Post-treatment**	**Material**	**Morphology**	**Diameter**	**Response**	**Optimum Operating Temperature**	**Detection Range**	**Response/Recovery Time**	**Analyte Gas**	**Ref.**
Conductometric	PVP	DMF:ethanol (1:1)	$In(NO_3)_3.4.5H_2O$ and H_2PtCl_6	ID = 0.8 mm		15	20	700 °C for 4 h	Pt doped In_2O_3	NFs	160–200 nm	1490/600 ppm	200 °C	50 ppm – 600 ppm	60s / 120s	H_2S	[24]
Conductometric	PVP	DMF	$In(NO_3)_3.4.5H_2O$ and $HAuCl_4$	--	--	22	20	550 °C	Au doped In_2O_3	NFs	80 – 100 nm	13.8/500 ppm	140 °C	10 ppm – 4000 ppm	12s/24s	ethanol	[25]
Conductometric	PVA	DI water	zinc acetate and $Ni(NO_3)_2$	--	--	8	20	650 °C for 3 h in air	Ni doped ZnO	NFs	100 nm	16.9/2000 ppm	250 °C	2000 ppm	5s/10 s	C_2H_2	[26]
Conductometric	PMMA	DMF, DI water, PDADMAC	$(NH_4)_6H_2W_{12}O_{40}\cdot xH_2O$<br>$K_2PtCl_4$<br>$K_2PdCl_4$	25G	0.2 ml/min	15	15	500 °C for 1 h in air	WO_3<br><br>Pt – WO_3,	NTs	1.57 µm – 3.37 µ	63.59/ 5 ppm toward NO 1.05/ 5 ppm toward toluene<br><br>4.22/ 5 ppm toward NO 2.74/5 ppm toward toluene	350 °C	50 ppb - 5ppm	--	NO, Toluene	[27]

	Electrospinning parameters											Sensing Performance					
**Sensing Platform**	**Polymer**	**Solvent**	**Precursor**	**Needle Type**	**Flow Rate**	**Applied Voltage (KV)**	**Working Distance (cm)**	**Post-treatment**	**Material**	**Morphology**	**Diameter**	**Response**	**Optimum Operating Temperature**	**Detection Range**	**Response/Recovery Time**	**Analyte Gas**	**Ref.**
									Pd – WO_3			3.89/5 ppm toward NO 2.60/5 ppm toward toluene					
Conductometric	PVP	DMF and ethanol	$C_{16}H_{36}O_4Ti$	--	--	16	--	650 °C for 4 h in air	TiO_2	NRs	500 nm	20/1000 ppm	500 °C	50 - 500 ppm	11-14s/ 4-8s	acetone	[28]
Conductometric	PVP	DMF, Ethanol	$Zn(AC)_2{\cdot}2H_2O$) And $Pr(NO_3)_3{\cdot}6H_2O$	--	--	17	20	600 °C for 3 h in air	Pr-doped ZnO	NFs	180 – 270 nm	3.71/100 ppm	380 °C	20 ppm – 800 ppm	51s/40s	acetic acid	[29]
Conductometric	PVP	DMF:ethanol (1:1)	$SnCl_2{\cdot}2H_2O$ and $ZnCl_2$	ID = 0.8 mm	20 μl/min	10	20	600 °C for 5 h in air	ZnO–SnO_2	NFs	100 – 200 nm	18 / 100 ppm	300 °C	1 - 10000	5s/6s	ethanol	[30]
Conductometric	PMMA	DMF	SWCNTs	--	0.1 – 1.5 ml/h	10 – 30	--	--	PMMA/CNTs	NFs	200–500 nm	1.3/2083 ppm	RT	1 – 3471 ppm	--	methanol	[31]
Conductometric	PVP	glacial acetic , ethanol, and water	$Zn(AC)_2.2H_2O$, hexamethylenetetramine and Tetrabutyl titanate	--	--	20	--	500 °C for 2 h in air	TiO_2/ZnO	(NSs on NFs) heterostructures	70 – 100 nm	15.7/100 ppm	280 °C	10 – 200 ppm	5s/3s	ethanol	[32]
Conductometric	PVP	DMF:ethanol (1:1)	$Zn(AC)_2.2H_2O$ and $PdCl_2$	0.25 mm	0.05 ml/h	18	20	600 °C for 3 h in air	Pd-doped ZnO	NFs	70 – 160 nm	5.5/20 ppm	220 °C	1 – 20 ppm	25-29s /12-17s	CO	[33]
Conductometric	PVP	DI Water	$(NH_4)_6H_2W_{12}O_{40}{\cdot}xH_2O$ and K_2PdCl_4	ID = 0.8 mm OD = 1.6	0.01 ml/ for core	30	15	600 °C for 1 h in air	Pd functionalized WO_3 NTs	NTs	--	17.6/500 ppm	450 °C	10 – 500 ppm	25s/ --	H_2	

	Electrospinning parameters											Sensing Performance					
**Sensing Platform**	**Polymer**	**Solvent**	**Precursor**	**Needle Type**	**Flow Rate**	**Applied Voltage (KV)**	**Working Distance (cm)**	**Post-treatment**	**Material**	**Morphology**	**Diameter**	**Response**	**Optimum Operating Temperature**	**Detection Range**	**Response/Recovery Time**	**Analyte Gas**	**Ref.**
				mm+	and 0.03 – 0.1 ml/h for core												
Conductometric	PVP	DMF:ethanol (1:1)	$In(NO_3)_3$ and $Co(NO_3)_2.6H_2O$	--	--	15	20	500 °C for 2 h in air	Co–In oxide	NFs	50 – 90 nm	41.5/100 ppm	260 °C	1 – 100 ppm	3s/25 s	ethanol	[35]
										NTs	150 – 200 nm	93.1/100 ppm			3s/72 s		
Conductometric	PVA	DI water	$Zn(AC)_2$	0.51 mm	0.5 ml/h	10	20	700 °C for 2 h in air	ZnO	NFs	150 – 200 nm	15/5 ppm	300 °C	1 ppm – 5 ppm	--	CO	[36]
Conductometric	PMMA, PS, PPy	DMF	BPO	--	6 ml/h	8 – 9	10	--	PPy/PMMA	NFs	450 nm	14%/150 ppm	--	150 ppm	300s/-	NH_3	[37]
Conductometric	PCL	HCSA, HFIP	PANI	--	0.3 ml/h	5.8	55		PANI–PCL	NFs	150 nm	2.88%/ppm	--	1 – 75% saturation	--	H_2O vapours	[38]
												40.0%/ppm		0.5 ppm – 100 ppm		NH_3	
												251%/ppm		0.1 ppm – 10 ppm		NO_2	
Conductometric	PVP	Water, ethanol	$In(NO_3)_3.4.5H_2O$	ID = 0.45mm, OD = 0.5 mm	--	20	20	500 °C for 1 h and then 700 °C for 2 h	In_2O_3	NFs	50 – 150 nm	~3.7/30 ppm (V/v)	220 °C	1 ppm – 30 ppm	6s/10 s	ethanol	[39]
Conductom	PVP	DMF:C_2H_5OH:CH_3	$In(NO_3)_3·4.5H_2O$	--	0.4	15	15	600 °C	In_2O_3	NTs	100	11.9/50	240 °C	1 ppm	10s/1	HCHO	[40]

	Electrospinning parameters											Sensing Performance					
**Sensing Platform**	**Polymer**	**Solvent**	**Precursor**	**Needle Type**	**Flow Rate**	**Applied Voltage (KV)**	**Working Distance (cm)**	**Post-treatment**	**Material**	**Morphology**	**Diameter**	**Response**	**Optimum Operating Temperature**	**Detection Range**	**Response/Recovery Time**	**Analyte Gas**	**Ref.**
etric		COOH			ml/h for inner core – 0.6 ml/h for outer core			for 3 h in air			nm – 1100 nm	ppm		– 800 ppm	5s		
Conductometric	PVP	DMF, ethanol	$ZnNO_3$ and chromium nitrate	--	--	12	15	600 °C for 5 h in air	Cr_2O_3-sensitized ZnO	NFs	80 nm – 130 nm	24/100 ppm	300 °C	1 ppm – 200 ppm	1s/5s	ethanol	[41]
Conductometric	PVAc	DMF, ethanol	$CuCl_2 \cdot 2H_2O$ and $SnCl_2 \cdot 2H_2O$	21G	0.03 ml/h	15	20	700 °C for 1h in air	CuO/SnO_2	Mixed NFs	110 nm	522/10 ppm	300 °C	10 ppm – 100 ppm	1s/305s	H_2S	[42]
Conductometric	PVP	DMF:ethanol (1:1)	$Co(NO_3)_2{\cdot}6H_2O$	21G	0.5 ml/h	20	15	500, 600, and 700 °C for 2 h in an air	Co_3O_4	NFs	100 nm – 200 nm	51.2/100 ppm	301 °C	5 ppm – 100 ppm	22.7s /2.4s	ethanol	[43]
Conductometric	PVP	DMF:ethanol (1:1)	$SnCl_2{\cdot}2H_2O$ and $PdCl_2$	--	--	20	10	600 °C for 2 h air	Pd-doped SnO_2	HNFs	200 nm – 300 nm	24.6/100 ppm<br>11.3/100 ppm<br>8.5/500 ppm<br>1020.6/100 ppm	385 °C and 440 °C	1 – 500 ppm	0.4s/ 11.4s<br>15.1s /4.7s<br>0.8s/ 12.3s<br>1.0s/ 9.6s	H_2<br>CO<br>CH_4<br>C_2H_5OH	[44]
Conductometric	PVP	DMF and ethanol	$In(NO_3)_3{\cdot}4.5H_2O$ and tetra-butyl titanate	--	0.25 ml/h	16	--	550 °C for 4 h in air	TiO_2−In_2O_3	Hemi-micelled NFs	150 nm – 250 nm	95/97 ppm	RT	0.3 ppm – 97 ppm	4.3s/--	NO_2	[45]

	Electrospinning parameters											Sensing Performance					
**Sensing Platform**	**Polymer**	**Solvent**	**Precursor**	**Needle Type**	**Flow Rate**	**Applied Voltage (KV)**	**Working Distance (cm)**	**Post-treatment**	**Material**	**Morphology**	**Diameter**	**Response**	**Optimum Operating Temperature**	**Detection Range**	**Response/Recovery Time**	**Analyte Gas**	**Ref.**
Conductometric	PEO	DI water, CSA and methanol	PANI and $Zn(CH_3COO)_2$	--	0.4 ml/h	18	18	--	PANI/ZnO	NFs	200 nm – 300 nm	133.6/1000 ppm	36 °C	1000 ppm	150s/185s	LPG	[46]
Conductometric	PVP	DMF	$Zn(AC)_2{\cdot}H_2O$	--	--	20	20	600 °C for 3 h in air	ZnO	NFs	150 – 200 nm	51/100 ppm	270 °C	10 – 2000 ppm	7–9s/9 – 11s	ethanol	[47]
Conductometric	PVP	DMF:ethanol (1:1)	$Zn(NO_3)_2$. $6H_2O$ and $In(NO_3)_3$. $4.5H_2O$	--	0.4 ml/h	15	15	500 °C for 2 h in air	IZO	NTs	60 – 80 nm	81.8/100 ppm	275 °C	5 – 5000 ppm	2 – 6s/56 – 63s	ethanol	[48]
Conductometric	PVP	DMF:ethanol (1:1), ammonium persulfate (APS), and HCl	$In(NO_3)_3{\cdot}4.5H_2O$ and polyaniline	0.5 mm	0.5 ml/h	16	16	800 °C for 3 h in air	In_2O_3/PANI	NFs<br>HNFs	850 nm	11.4/1000 ppm<br>52.4/1000 ppm	RT	100 ppm – 1000 ppm	--	NH_3	[49]
Conductometric	PVP	DMF and ethanol	$Zn(CH_3COO)_2{\cdot}H_2O$ and $Mn(CH_3COO)_2$	--	--	15	25	580 °C for 150 min in air	Mn-doped ZnO	NFs	60 nm – 90 nm	203.9/450 ppm	340 °C	50 ppm – 1000 ppm	6s/4s	acetone	[50]
Conductometric	PVP	DMF:ethanol (1:1)	$SnCl_2{\cdot}2H_2O$, $LaCl_3$	--	1 ml/h	20	--	600 °C for 2 h in air	LaOCl-doped SnO_2	NFs	180 nm	3.7/1000 ppm	300 °C	100 ppm – 20000 ppm	24s/92s	CO_2	[51]
Conductometric	PVP	DMF and ethanol	$Zn(NO_3)_2$	0.7 mm	--	22	--	500 °C for 3 h in air	ZnO	HNFs	60 nm	4.7/100 ppb<br>3.0/120 ppb	170 °C	10 ppb – 30 ppm	10s/--<br>100s/--	Nitrotoluene<br>Di-Nitrotoluene	[52]

	Electrospinning parameters											Sensing Performance					
**Sensing Platform**	**Polymer**	**Solvent**	**Precursor**	**Needle Type**	**Flow Rate**	**Applied Voltage (KV)**	**Working Distance (cm)**	**Post-treatment**	**Material**	**Morphology**	**Diameter**	**Response**	**Optimum Operating Temperature**	**Detection Range**	**Response/Recovery Time**	**Analyte Gas**	**Ref.**
															1s/--	Nitromethane	
												29/30 ppm			1s/--	Nitroethane	
												83/30 ppm					
Conductometric	PVP	DMF:ethanol (1:1)	$SnCl_2·2H_2O$ and $Al(NO_3)_3·9H_2O$	1 mm	--	15	20	600 °C for 5h in air	Al-doped SnO_2	NFs	80 nm – 120 nm	7.7/100 ppm	340 °C	10 ppm – 30,000 ppm	3s/2s	H_2	[53]
Conductometric	PVP	DMF:ethanol (1:1)	$SnCl_2·2H_2O$ and $Fe(NO_3)_3·9H_2O$	--	0.4 ml/h	15	15	500 °C for 2 h in air	SnO_2-doped α-Fe_2O_3	NTs	65 nm	27.45/100 ppm	200 °C	5 ppm – 10,000 ppm	3s/14 s	ethanol	[54]
												10.07/100 ppm			4s/12 s	acetone	
Conductometric	PEO	HCSA and $CHCl_3$	PANI	--	--	8	--	--	HCSA-doped PANI	NFs	20–150 nm	94/--	RT	--	32s/20s	methanol	[55]
												94/--					
												97/--			20s/20s	ethanol	
															110s/50s	2-propanol	
Conductometric	PEO	--	PEDOT:PSSA	--	--	10	20	--	PEDOT-PSSA	NFs	5 nm – 50 nm	40%/128000 ppm	RT	--	8s/--	methanol	[56]
												40%/60000 ppm					
												30%/13000 ppm			10s/--	ethanol	
												20%/23000 ppm			20s/--	2-propanol	
												30%/150000 ppm			240s/--	H_2O	

	Electrospinning parameters											Sensing Performance					
**Sensing Platform**	**Polymer**	**Solvent**	**Precursor**	**Needle Type**	**Flow Rate**	**Applied Voltage (KV)**	**Working Distance (cm)**	**Post-treatment**	**Material**	**Morphology**	**Diameter**	**Response**	**Optimum Operating Temperature**	**Detection Range**	**Response/Recovery Time**	**Analyte Gas**	**Ref.**
												20%/247000 ppm			13s/--	NH_3	
												30%/1000 ppm			95s/--	HCl	
															120s/--	NO_2	
Conductometric	PVP	DMF	$In(NO_3)_3·4.5H_2O$	--	--	16	15	600 °C for 2 h in air	In_2O_3	NTs<br>NWs	80 nm<br>120 nm	166.6/20 ppm<br>141/20 ppm	RT	1-100 ppm	287s/636s<br>199s/317s	H_2S	[57]
Conductometric	PVP	DMF and ethanol	$In(NO_3)_3$ and $Fe(NO_3)_3$	--	--	15	20	550 ºC fir 2 h	Fe_2O_3-In_2O_3 composite	NTs	200 nm	33/100 ppm	240 ºC	1-100 ppm	5s /25s	formaldehyde	[58]
Conductometric	PVP	DMF	$Zn(CH_3COO)_2·2H_2O$ and $In(NO_3)_3·xH_2O$	25 G	0.01 ml/h	17	15	600 °C for 2 h in air	ZnO-In_2O_3 composite	NFs	--	119.4/5 ppm	375 °C	0.05 - 5 ppm	1-2s/1603s	trimethylamine	[59]
Conductometric	PVP	DMF	$SnCl_2.2H_2O$ and $In(NO_3)_3.4.5H_2O$	--	0.1 ml/h	15	15	350 °C for 1h - 600 °C for 3 h in air	SnO_2/In_2O_3	NTs	80 – 120 nm	400/500 ppm	300 ºC	250 ppb-500 ppm	60s/97s	formaldehyde	[60]
Conductometric	PVP	DMF and ethanol	$SnCl_2·2H_2O$ and $In(NO_3)_3·41/2H_2O$	--	--	--	5	600 °C for 2 h in air	SnO_2/In_2O_3 composite	NFs	200 – 250 nm	35.69/50 ppm	290 ºC	0.5 ppm – 50 ppm	20s/40s	formaldehyde	[61]
Conductometric	PVP	ethanol and glacial acetic	tetrabutyl titanate and $Zn(Ac)_2·2H_2O$	--	--	20	--	500 °C for 2 h in air	ZnO-TiO_2	NFs	100 - 300 nm	50.6 /500 ppm	320 ºC	20-500 ppm	50.6s /5 - 10s	ethanol	[62]
Conductometric	PVP	DMF, ethanol and Acetic Acid	tungsten chloride and	ID = 1.01 mm	0.5 ml/h	20	15	500 °C for 2 h	In_2O_3 - WO_3	NFs	170 nm	12.9/50 ppm	275 ºC	0.4 ppm –	6s/64 s	acetone	[63]

	Electrospinning parameters											Sensing Performance					
**Sensing Platform**	**Polymer**	**Solvent**	**Precursor**	**Needle Type**	**Flow Rate**	**Applied Voltage (KV)**	**Working Distance (cm)**	**Post-treatment**	**Material**	**Morphology**	**Diameter**	**Response**	**Optimum Operating Temperature**	**Detection Range**	**Response/Recovery Time**	**Analyte Gas**	**Ref.**
			$In(NO_3)_3{\cdot}4.5H_2O$					in air						50 ppm			
Conductometric	PVP	DMF:ethanol (1:1)	$In(NO_3)_3{\cdot}4.5H_2O$ and $Sn(OH)_4$	--	1.0 ml/h	20	25	500 °C for 2 h in air	SnO_2/In_2O_3	NFs	30 – 80 nm	21/1 ppm	RT	0.1 ppm-1 ppm	7s/10 s	NH_3	[64]
Conductometric	--	DMF	PMMA and PANI	hyperdermic needle	0.2 ml/h	20	15	--	PANI/PMMA composite	NFs	400 - 600 nm	77/500 ppm	RT	20-500 ppm	131s/600s	Triethylamine	[65]
Conductometric	PANI/PA6	Formic acid	TiO_2 (sputtered)	--	--	--	--	--	TiO_2-PANI/PA6	NFs	--	18.3/250 ppm	RT	50 ppm – 250 ppm	--	NH_3	[66]
Conductometric	PVP	Acetylacetone, DI water and ethanol	titanium(IV) butoxide	--	--	20	15	600 °C for 3 h	PANI/TiO_2 composite	NFs	72-90 nm	--	RT	25 - 200 ppb	--	NH_3	[67]
Conductometric	PVP	acetic acid and ethanol	EDOT $Ti(OiPr)_4$	23 G	0.3 ml/h	7	5	500 °C for 3 h	TiO_2-PEDOT	Core-sheath nanocable	Core = ~78 nm	0.86%/300 ppb	RT	7-300 ppb	--	NO_2	[68]
												0.222%/1o ppm		675 ppb-10 ppm	--	NH_3	
Conductometric	PVB	DMF and ethanol	$SnCl_2{\cdot}2H_2O$	ID = 0.7 mm	0.2 ml/h	8	15	--	SnO_2 /PPy	NFs	100-200 nm	~6.2%/ppm In range of 1-10.7 ppm	RT	~257 ppb-10.7 ppm	259s/468 s	NH_3	[69]
Conductometric	PAN	DMF	$SnCl_2.2H_2O$	--	--	15	20	800 °C	SnO_2-carbon	NFs	150 - 500 nm	~16.3/100 ppm	200 °C	100-35,000 ppm	4s/16s	H_2	[70]
Conductometric	PAN	DMF	$SnCl_4.5H_2O$	ID = 0.5 mm	0.4 ml/h	10	20	270 °C for 2 h	Sn-SnO_2/car	NFs	350-400	46.15/1000 ppm	240 °C	10-2000	--	ethanol	[71]

	**Electrospinning parameters**											**Sensing Performance**					
**Sensing Platform**	**Polymer**	**Solvent**	**Precursor**	**Needle Type**	**Flow Rate**	**Applied Voltage (KV)**	**Working Distance (cm)**	**Post-treatment**	**Material**	**Morphology**	**Diameter**	**Response**	**Optimum Operating Temperature**	**Detection Range**	**Response/Recovery Time**	**Analyte Gas**	**Ref.**
								in air, and 800 °C for 30 min in Ar	bon		nm			ppm			
Conductometric	PVA	DI Water	$(CH_3CO_2)_2Zn$ and rGO	21 G	0.03 ml/h	15	20	500 °C for 5 h in air	rGO-ZnO	NFs	150 nm	119/5 ppm	400 ºC	1 ppm	143s/ 259s	NO_2	[72]
Conductometric	PVAc	DMF and ethanol	$SnCl_2.2H_2O$ graphene	--	--	--	--	600 °C for 30 min in air	rGO-SnO_2	NFs	200 - 300 nm	3.13/1ppm	300 °C	1 ppm	51.2 s/330 s	C_7H_8	[73]
Conductometric	PVAc	DMF and ethanol	$SnCl_2.2H_2O$<br>GO<br>$PdCl_2$<br>H2PtCl6.nH_2O	ID = 0.51 mm	0.03 ml/h	15	20	600 ºC for 30 min in air	SnO_2	NFs	--	1.6/1 ppm	200 °C	1 ppm- 5 ppm	--	C_6H_6	[74]
									rGO-SnO_2			3.3/1 ppm				C_6H_6	
									rGO-Pd-SnO_2			8.3/1 ppm				C_6H_6	
									rGO-Pt-SnO_2			255.5%/1 ppm				C_7H_8	
Conductometric	PVP	DMF and ethanol (2:1)	$SnCl_2.2H_2O$<br>rGO	--	--	--	--	450 °C for 2 h in air	rGO/SnO_2	NFs	--	32.2/10 ppm	150 °C	--	6 min/30 min	NO_2	[75]
SAW	PEO	DI Water and ethanol	polyethylene glycol (PEG)	--	--	27	25	--	PEO	NFs	100 – 300 nm	-4 Hz/MHz /30%	RT	10% - 90% saturated vapours	60 – 120 s/60 s	Isopropanol	[76]
												-20 Hz/MHz /30%				nitrobenzene	
												-32 Hz/MHz /30%				toluene	
												-30 Hz/MHz /30%				hydrogen peroxide	

	Electrospinning parameters											Sensing Performance					
Sensing Platform	Polymer	Solvent	Precursor	Needle Type	Flow Rate	Applied Voltage (KV)	Working Distance (cm)	Post-treatment	Material	Morphology	Diameter	Response	Optimum Operating Temperature	Detection Range	Response/Recovery Time	Analyte Gas	Ref.
SAW	--	DMF	PVB	--	0.5 ml/h	6	15	--	PVB	NFs	--	0.425 MHz/90% RH	RT	10 %-90% RH	--	Humidity	[77]
		Chloroform	PEO	--	0.5 ml/h	4	15		PEO			3.428 MHz/90% RH					
		Ethanol	PVP	--	0.5 ml/h	6	15		PVP			8.134 MHz/90% RH					
		DMF	PMMA	--	1 ml/h	6	12		PMMA			0.312 MHz/90% RH					
		DMF	PVDF	--	1 ml/h	6	12		PVDF			0.334 MHz/90% RH					
SAW	PANI	DMF	PVB	--	0.5 ml/h	10	12	100 °C for 2 h	PANI/PVB	NFs	100-200 nm	~75 kHz/%RH from 20 to 90%RH	RT	0.5%-90% RH	1s/2s	Humidity	[77]
SAW	PVP	ethanol	CeO_2	--	--	12	12	65 °C for 12 h	CeO_2-PVP	NFs	450 nm	-2.5 MHz/from 11% to 95% RH	RT	11%-95% RH	~16s/16s	Humidity	[78]
SAW	PVP	DI water	PVP	--	--	12	8.5	--	PVP	NFs	340 nm	5.6kHZ/1%	RT	0.125-1%	10s/200s	Hydrogen	[79]
SAW	PVP	alcohol	MWCNTs, Nafion	--	0.2 ml /h	15	10	--	MWCNTs/Nafion	NFs	--	400 kHz/%RH in the range of 10%-80% RH	RT	1%-80%RH	3s/--	humidity	[80]
QCM	PVP	ethanol	PVP	--	100 µl/h	19	10	--	PVP	Fs	7-12 µm	~220 Hz/5 mg/l	RT	5-30 mg/l	500s/2700 s	ethanol	[81]
QCM	PEI, PS	Water, ethanol,	PS, PEI	--	4	20	15	dried	PEI-PS	NFs	110 -	19	RT	3-140	--	formaldeh	[82]

	Electrospinning parameters											Sensing Performance					
**Sensing Platform**	**Polymer**	**Solvent**	**Precursor**	**Needle Type**	**Flow Rate**	**Applied Voltage (KV)**	**Working Distance (cm)**	**Post-treatment**	**Material**	**Morphology**	**Diameter**	**Response**	**Optimum Operating Temperature**	**Detection Range**	**Response/Recovery Time**	**Analyte Gas**	**Ref.**
		THF, DMF			ml/h			at 25 °C in vacuum for 2 h			870 nm	Hz/140 ppm		ppm		yde	
QCM	PS	Water, ethanol, THF, DMF	PEI, PA 6	--	1 ml/h	20	15	dried at 25 °C in vacuum for 12 h	PEI-PA 6	NFN	26 nm	52.8 Hz/100 ppm	RT	50 ppb-100 ppm	100s/--	formaldehyde	[83]
QCM	PVA	H_2O and ethanol	PAA	--	--	20	5	--	PAA/PVA	NFs	350 nm	60 Hz /1 ppm	RT	130 ppb-200 ppm	10min/60min	NH_3	[84]
QCM	PS-*b*-PMA	Acetone and DMF	PS-*b*-PMA	--	0.8 ml/h	25	19	dried in vacuum at RT for 1 h	PS-*b*-PMA	NFs	261-744 nm	28.2 Hz/50 ppm	RT	1.5-50 ppm	--	NH_3	[85]
QCM	PA and PS	DMF and ethanol	--	--	1 ml/h	25	19	dried for 1 h at 70 °C in vacuum	PA/PS	NFs	488 nm	2.1 Hz/25 ppm	RT	1.5-50 ppm	--	NH_3	[86]
QCM	PVA and PAA	DI water	--	--	--	15	10	dried at 80 °C in vacuum for 2 h	PVA-PAA	NFs	200-330 nm	40 Hz/50 ppm at 0%RH 330 Hz/50 ppm at 60%RH	RT	50-200 ppm	--	NH_3	[87]

	Electrospinning parameters											Sensing Performance					
**Sensing Platform**	**Polymer**	**Solvent**	**Precursor**	**Needle Type**	**Flow Rate**	**Applied Voltage (KV)**	**Working Distance (cm)**	**Post-treatment**	**Material**	**Morphology**	**Diameter**	**Response**	**Optimum Operating Temperature**	**Detection Range**	**Response/Recovery Time**	**Analyte Gas**	**Ref.**
QCM	PS	THF, DMF, acetic acid and ethanol	titanium tetraisopropoxide, PEI	--	4 ml/h	20	20	at 450 °C for 5 h in air	PEI-TiO_2	NFs	625 nm	13.7/100 ppm	RT	1-100 ppm	120s/--	formaldehyde	[88]
QCM	PS	THF, DMF and DI water	carboxyl graphene (G-COOH), PS	--	1 ml /h	25	19	dried at 80 °C in vacuum for 1 h	G-COOH/PS	NFs	569 nm	3.5 Hz/40 ppm	RT	1-40 ppm	--	NH_3	[89]
Optical (photoluminescence spectroscopy)	PAN	DMF	--	0.7 mm	3 ml/h	25	25	--	PAN/ZnO	NFs	250-350 nm	NBE= 0.83 ± 0.04 DLE= 1.31 ± 0.03	RT	150 ppm	--	ethanol	[90]
Optical (FTIR spectroscopy)	PAN	--	Fe_2O_3, ZnO, Sb-SnO_2 NPs	--	--	--	--	--	PAN-MOx	NFs	50 - 150 nm	Highest response for PAN-Fe_2O_3	RT	2000 ppm	--	CO_2	[91]
Optical (fluorescence quenching)	PS	DMF	**P**	--	0.6 ml/h	25	25	40 °C for 12 h	**P**	NFs	800 - 1000 nm	fluorescence quenching efficiency is PA>TNT>DNT>NB with values of 85%, 65%, 25% and 12%	RT	0.5-3 mM	--	PA, TNT, DNT, NB	[92-93]
Optical (absorbance)	PAA	water	--	--	1 ml/h	18.5	22	--	PAA	NFs	0.59± 0.15 µm	2 dB/95% RH	RT	30%-95%	340ms/210 ms	Humidity	[94]

	Electrospinning parameters											Sensing Performance					
**Sensing Platform**	**Polymer**	**Solvent**	**Precursor**	**Needle Type**	**Flow Rate**	**Applied Voltage (KV)**	**Working Distance (cm)**	**Post-treatment**	**Material**	**Morphology**	**Diameter**	**Response**	**Optimum Operating Temperature**	**Detection Range**	**Response/Recovery Time**	**Analyte Gas**	**Ref.**
Optical (fluorescence intensity)	--	HFP, DCM, Toluene	PCL, PDMS	--	1 ml/h	25	20	--	PDMS - PCL	Core-shell NFs	570± 192 nm	24/100%	RT	20-100%	0.49 ± 0.13s /0.70 ± 0.15s	oxygen	[95]
Optical (luminescence)	PS	2-butanone, cationic surfactant hexadecyl-trimethyl ammoniumbromide	PtTFPP	a hypodermic needle with ID = 0.2 mm	0.5 ml/min	10	10	80 ºC at 1 mbar for 1 h	PtTFPP	Band like	Width:620 nm thickness: 100 nm	--	RT	100%	2.2s/--	oxygen	[96-97]
Optical (fluorescence intensity)	PPI	DMAc	--	--	1 ml/h	16	12	60 °C	PPI	NFs	350 ± 54nm	~0.95 a.u./50 ppm	RT	1.25 ppm – 100 ppm	10s/--	HCl	[98]

PVA (polyvinyl alcohol); PEI (Polyethyleneimine); PANI (polyaniline); PMMA (Polymethyl methacrylate); PS (polystyrene); PVP (Polyvinylpyrrolidone); PAA (polyacrylic acid); PAN (Polyacrylonitrile); DMF (Dimethyl Formamide); DMSO (Dimethyl Sulfoxide); THF (Tetra Hydro Furan); PA polymer (Phenyl acetic acid); PPy (polypyrrole); PPI (porphyrinated polyimide); PCL (polycaprolactone); BPO (benzoyl peroxide); HCSA (Camphorsulfonic acid); PEO (polyethylene); CSA ((±)-10-camphorsulfonic acid); LPG (liquefied petroleum gas); HCl (hydrochloric acid); RT (room temperature); DI (distilled water); OD (outer diameter); ID (inner diameter); NFs (nanofibers); NTs (nanotubes); NWs (nanowires); HNFs (hollow nanofibers); NSs (nanosheets); NRs (nanorods); NRbs (nanoribbons); HFs (hollow fibers); rGO (reduced graphene oxide); RH (relative humidity); MWCNTs (multi carbon nanotubes); CNTs (carbon nanotubes); NFN (nanofiber net); NBE (near band emission); DLE (deep level emission); PA (picric acid), TNT (trinitrotoluene), DNT (2,4-dinitrotoluene), NB (nitrobenzene); **P** (benzothiophene based conjugated polymer with sulfur-containing fused rings as the backbone); HFIP (1,1,1,3,3,3-hexafluoro-2-propanol); DCM (Dichloromethane);

PtTFPP (platinum-tetra(pentafluorophenyl) porphyrine); PEDOT (polyethylenedioxythiophene); PSSA (poly(styrene sulfonic acid)); PVB (poly(vinyl butyral)); PVDF (poly(vinylidene fluoride)); DMAc (*N*,*N′*-Dimethyl acetamide)

Table S2: Sensing performance of electrospun pure MOx nanofibers categorized based on the analyte gas.

Material	Morphology	Diameter (nm)	Operating Temperature (°C)	Response	Analyte gas	Response /Recovery time	Reference
TiO_2	NFs	400 – 500	200	4.4/25 ppm	CO	32–86 s/ 84–109 s	[99]
TiO_2	NFs	350 – 500	180	3.4/25 ppm	CO	32–86 s/ 84–109 s	[100]
TiO_2	HNFs	200	RT	Rg 70K/0.1 ppm	CO	-	[101]
SnO_2	NFs	200 – 400	300	3.8/500 ppm	CO	260 s/15 min	[102]
ZnO	NFs	35 – 150	200	1.51/2 ppm	CO	168–237 s/270–350 s	[103]
In_2O_3	NFs	100	300	~540%/100 ppm	CO	-	[8]
TiO_2	NRs	500	500	9.0/10 ppm	Acetone	11–14 s/4–8 s	[28]
WO_3	NFs	275	270	55.6/50 ppm	Acetone	6-13 s/4-9 s	[104]
WO_3	NTs	200	250	19.7/40 ppm	Acetone	5 s/22 s	[105]
SnO_2	NBs	140	260	6.7/5 ppm	Acetone	38 s / 9 s	[106]
ZnO	HNFs	145	220	7.1/1 ppm	Acetone	12–17 s/11–23 s	[107]
ZnO	NTs	95	500	12.3/2000 ppm	Acetone	5 s/10 s	[108]
In_2O_3	HNFs	250 – 310	300	151/5 ppm	Acetone	5 s/2 s	[109]
TiO_2	NFs	120 – 200	450	30/50 ppm	NO_2	2–4 min/20 s	[110]
WO_3	NFs	100	75	12.4/400 ppb	NO_2	33 min/38 min	[3, 110-111]
SnO_2	NFs	200 – 400	185	368/50 ppm	NO_2	400 s/200 s	[102, 105]
SnO_2	HNFs	300–500	300	81.4/2 ppm	NO_2	55 s/5 min	[3, 111-112]
In_2O_3	NRs	20 – 300	200	~68.5/12 ppm	NO_2	43 min/93 min	[9, 104]
α-Fe_2O_3	NFs	150 – 280	300	2.3/100 ppm	Ethanol	3 s/5 s	[105, 113]
SnO_2	NTs	200	300	76/500 ppm	Ethanol	12 s/8 s	[113-114]
SnO_2	NFs	100	330	4.5/10 ppm	Ethanol	13 s/13.9 s	[14-15]

Material	Morphology	Diameter (nm)	Operating Temperature (ºC)	Response	Analyte gas	Response /Recovery time	Reference
In_2O_3	NFs	160 – 200	300	379/15000 ppm	Ethanol	1 s/5 s	[114-115]
In_2O_3	NFs	30 – 100	220	~3.75/30 ppm	Ethanol	6 s/10 s	[39, 106]
Co_3O_4	NFs	100 – 200	301	51.2/100 ppm	Ethanol	7.9s-22.7 s/58.7s-2.4 s	[43]
SnO_2	NTs	80 – 400	150	2.4/1.0 ppm	H_2	21 s/333 s	[14-15]
ZnO	NTs	200	200	3.6/100 ppm	H_2	20 min/10 min	[102, 116]
ZnO	NFs	250	350	109.1/10 ppm	H_2	-	[102, 117]
ZnO	NFs	500 – 600	RT	12.61/100 ppm	Formaldehyde	32 s/17 s	[107, 118]
In_2O_3	NFs	150 - 200	340	3.11/100 ppm	Formaldehyde	18 s/17 s	[118-119]
In_2O_3	NWs	120	RT	141.1/20 ppm	H_2S	199 s/317 s	[57]
In_2O_3	NTs	80	RT	166.6/20 ppm	H_2S	287 s/636 s	[57]
In_2O_3	NTs	370	340	5.88/40 ppm	Toluene	3 s/17 s	[39, 120]
WO_3	NFs	200	200	5.5/100 ppm	NH_3	1 s/5 s	[105]

NBs (nanobelts)

Table S3: Sensing performance of electrospun metal doped MOx nanofibers categorized based on the analyte gas.

Material	Structure (NTs/NFs/HNFs/NRs/NWs)	Dopant	Temperature (°C)	Response	Detection Limit	Response/recovery time	Analyte gas	Reference
SnO_2	HNFs	Y	300	174/500 ppm	50 ppm	9-30s/6-9s	Acetone	[22, 121]
SnO_2	HNFs	Ni	340	64.9/100 ppm	2 ppm	7s/30s	Acetone	[122]
SnO_2	NFs	Pd	275	98.8/100 ppm	1 ppm		Acetone	[123]
SnO_2	HNFs	Ag	160	117/200 ppm	5 ppm	6s/10s	Acetone	[124]
α-Fe_2O_3	NTs	La	240	26/50 ppm	1 ppm	3s/10s	Acetone	[125]
α-Fe_2O_3	NTs	Nd	240	44/50 ppm	500 ppb	19s/50s	Acetone	[126]
α-Fe_2O_3	NTs	Sm	240	33/50 ppm	500 ppb	6s/11s	Acetone	[127]
α-Fe_2O_3	NTs	Ce	240	21.5/50 ppm	1 ppm	3s/8s	Acetone	[128]
α-Fe_2O_3	NTs	Ca	200	24.9/100	5 ppm	1s/3s	Acetone	[129]
ZnO	NFs	Ce	230	71.2/500 ppm	10 ppm		Acetone	[130]
ZnO	HNFs	Ce	260	75.04/100 ppm	5 ppm	-	Acetone	[131]
ZnO	NFs	Mn	340	76.2/50 ppm	50 ppm	17s/4s	Acetone	[132]
ZnO	NFs	Co	360	16/100 ppm	5 ppm	6s/4s	Acetone	[133]
ZnO	NFs	La	340	64/200 ppm	-	16s/8s	Acetone	[134]
WO_3	NFs	La_2O_3	350	12.7/100 ppm	0.8 ppm		Acetone	[135]
WO_3	HNFs	Cu	300	6.43/20 ppm	0.25 ppm	5s/20s	Acetone	[136]
WO_3	Hemitube	Pt	350	4.11/2 ppm	120 ppb	-	Acetone	[137]
In_2O_3	NTs	Eu	240	20/20 ppm	200 ppb	3s/90s	Acetone	[138]
α-Fe_2O_3	NTs	Al_2O_3	240	41.8/50 ppm	300 ppb	20s/60s	Ethanol	[22]
α-Fe_2O_3	NTs	Ca	200	26.8/100	5 ppm	1s/26s	Ethanol	[129]
SnO_2	NFs	Cu	300	56/500 ppm	5 ppm	1s/10s	Ethanol	[139]
SnO_2	HNFs	Pr	300	64.33/200 ppm	1 ppm	116s/11s	Ethanol	[140-141]
SnO_2	NFs	Sr	260	18.9/100 ppm	-	2s/8s	Ethanol	[142]

Material	Structure (NTs/NFs/HNFs/NRs/NWs)	Dopant	Temperature (°C)	Response	Detection Limit	Response/recovery time	Analyte gas	Reference
SnO_2	HNFs	Yb	340	170/500 ppm	-	7s/8s	Ethanol	[139, 143]
SnO_2	HNFs	Ce	250	~265/50 ppm	-	-	Ethanol	[144]
SnO_2	NFs	Fe	300	15.3/100 ppm	10 ppm	1s/3s	Ethanol	[145]
SnO_2	NFs	Co	300	40.1/100 ppm	2 ppm	-	Ethanol	[146]
ZnO	NTs	In	275	81.7/100 ppm	-	-	Ethanol	[48]
ZnO	NFs	Er	240	37.3/200 ppm	1 ppm	12s/3s	Ethanol	[132]
ZnO	NFs	Al	250	8.6/100 ppm	-	5s/9s	Ethanol	[11]
ZnO	NFs	Cr_2O_3	300	24/100 ppm	1 ppm	1s/5s	Ethanol	[41]
In_2O_3	NWs	Co	300	16.5/100 ppm	-	2s/3s	Ethanol	[140, 147]
In_2O_3	NFs	Pd	200	18/50 ppm	1 ppm	1s/10s	Ethanol	[148-149]
In_2O_3	NTs	Mg	250	25.82/100 ppm	-	-	Ethanol	[12, 150]
In_2O_3	NTs	Eu_2O_3	260	44/50 ppm	0.2 ppm	3s/21s	Ethanol	[151]
In_2O_3	NFs	Au	140	13.8/500 ppm	50 ppm	12s/24s	Ethanol	[25]
SnO_2	NFs	Co_3O_4	300	38/100 ppm	-	-	Ethanol	[152]
NiO	NTs	Pt	400	11.7/100 ppm	1 ppm	-	Ethanol	[153]
ZnO-SnO_2	HNFs	Ag	200	128.6/100 ppm	1 ppm	-	Ethanol	[154]
α-Fe_2O_3	NWs	Pt	175	157/10 ppm	-	-	H_2S	[141]
ZnO	NFs	Cu	230	18.7/10 ppm	-	18s/20s	H_2S	[143, 155]
In_2O_3	NTs	Mg	150	173.14/10 ppm	-	-	H_2S	[12, 121]
In_2O_3	NFs	V	90	13.9/50 ppm	-	15s/18s	H_2S	[144, 156]
In_2O_3	NFs	Pt	200	1490/600 ppm	50 ppm	60s/120s	H_2S	[24]
SnO_2	HNFs	CuO	125	410/10 ppm	-	-	H_2S	[157]
SnO_2	NFs	Pt	300	5100/20 ppm	-	-	H_2S	[150]
WO_3	NFs	Pd	350	1.36/1 ppm	1 ppm	-	H_2S	[158]
TiO_2	NFs	Ag	350	~120/1 ppm	1 ppm	-	H_2S	[159]

Material	Structure (NTs/NFs/HNFs/NRs/NWs)	Dopant	Temperature (°C)	Response	Detection Limit	Response/recovery time	Analyte gas	Reference
SnO_2	NTs	Al	240	7.82/1000 ppb	-	-	Formaldehyde	[23]
SnO_2	HNFs	Pd	160	18.8/100 ppm	-	2s/7s	Formaldehyde	[160]
In_2O_3	NTs	Er	260	12/20 ppm	100 ppb	5s/38s	Formaldehyde	[122, 161]
In_2O_3	Ruptured NTs	Nd	240	46.8/100 ppm	100 ppb	8s/22s	Formaldehyde	[162]
In_2O_3	NTs	Nd	240	44.6/100 ppm	100 ppb	15s/50s	Formaldehyde	[163]
In_2O_3	NTs	Sm	240	54.37/100 ppm	100 ppb	9s/40s	Formaldehyde	[164]
In_2O_3	NFs	Ag	115	28/50 ppm	5 ppm	5s/10s	Formaldehyde	[165]
α-Fe_2O_3	NTs	SnO_2	220	25.4/500 ppm	1 ppm	-	Formaldehyde	[166]
SnO_2	NFs	V_2O_5	325	6.32/25 ppm	-	-	benzene	[167]
SnO_2	NFs	Al	340	7.7/100 ppm	10 ppm	-	H_2	[53]
SnO_2	NFs	Co	330	24/100 ppm	-	2s/3s	H_2	[149]
ZnO	NFs	Pr	380	7.38/400 ppm	-	51s/40s	acetic acid	[29]
ZnO	NFs	Ni	250	16.9/2000 ppm	-	5s/10s	C_2H_2	[26]
SnO_2	NFs	Au	300	18.98/10 ppm	1 ppm	-	CO	[168]
SnO_2	NFs	Au	300	84/5ppm	-	22s/235s	CO	[169]
ZnO	NFs	Pd	220	5.5/20 ppm	1 ppm	25-29s/12-17s	CO	[33]
NiO	NTs	W	375	8.74/200 ppm	15 ppm	178s/152s	xylene	[170]
TiO_2	NFs	Pd	180	38/2.1 ppm	0.16 ppm	-	NO_2	[171]
SnO_2	NFs	Pt	300	11.9/10 ppm	1 ppm	-	Toluene	[172]
WO_3	NFs	Pd	350	5.5/1 ppm	20 ppb	10.9s/16.1s	Toluene	[158]
WO_3	NFs	Au	250	229.7/100 ppm	1 ppm	5–43 s/ 10–122 s	n-butanol	[173]

Table S4: Sensing performance of electrospun MO_x-MO_x nanofibers categorized based on the analyte gas.

Material	Structure	Diameter (nm)	Operating Temperature (°C)	Response	Detection limit	Response/recovery time	Analyte gas	Reference
NiO/SnO_2	Heterojunctions NFs	180	300	27.5/100 ppm	50 ppb	2.9s/4.7s	Ethanol	[174]
SiO_2@SnO_2	core–shell NFs	160 – 320	-	37/200 ppm	50 ppm	13s/16s	Ethanol	[175]
SnO_2–ZnO	Heterostructured NFs	50 – 80	300	78/100 ppm	5 ppm	25s/9s	Ethanol	[176]
ZnO–SnO_2	HNFs	150	260	168.3/200 ppm	20 ppm	4-7s/4-5s	Ethanol	[16]
SnO_2/CeO_2	NTs	200	320	49.1/100 ppm	50 ppm	11s/10s	Ethanol	[177]
ZnO–SnO_2	Core-shell hetrostructure	370	200	392.29/100 ppm	5 ppm	74s/12s	Ethanol	[178]
SnO_2/α-Fe_2O_3	NTs	65	200	27.45/100 ppm	5 ppm	3s/14s	Ethanol	[54]
Sn-SnO_2/C	heterostructure NFs	350 – 400	220	46.15/1000 ppm	10 ppm	-	Ethanol	[179]
SnO_2/α-Fe_2O_3	hierarchically Core-shell HNFs	100 – 200	340	20.370/100 ppm	2 ppm	15s/25s	Ethanol	[180]
SnO_2/ZnO	Composite NFs	60 – 80	360	17.5/3000 ppm	27.7	5s/1s	Ethanol	[181]
ZnO–TiO_2	Hierarchical heterojunctions NFs	100 – 300 nm	320	50.6/500 ppm	20 ppm	50.6s/5–10 s	Ethanol	[62]
NiO-In_2O_3	Composite NFs	152	300	78/100 ppm	20 ppm	-	Ethanol	[182]
Eu_2O_3–In_2O_3	Composite NTs	160	260	44/50 ppm	0.2 ppm	3s/21s	Ethanol	[151]
TiO_2-SnO_2	core–shell heterostructure NFs	120 – 250	280	13.7/100 ppm	10 ppm	2s/60s	Acetone	[183]
SnO_2–ZnO	Heterojunctions NFs	120	300	84/100 ppm	5 ppm	19s/9s	Acetone	[184]
SnO_2/α-Fe_2O_3	NTs	65	200	10.07/100 ppm	5 ppm	4s/12s	Acetone	[54]
SnO_2/α-Fe_2O_3	hierarchically Core-shell HNFs	100 – 200	340	30.363/100 ppm	2 ppm	5s/13s	Acetone	[180]
a-Fe_2O_3/SnO_2	Cage-like composite NFs	160	275	5.3/100 ppm	100 ppm	1.5s/2.5s	Acetone	[185]
ZnO–In_2O_3	Composite NTs	300	280	43.2/60 ppm	0.125 ppm	5s/25s	Acetone	[186]
In_2O_3–Fe_2O_3	Composite NTs	130	240	25/100 ppm	1 ppm	3s/7 s	Acetone	[187]
In_2O_3–WO_3	Heterojunction NFs	170	275	12.9/50 ppm	0.4 ppm	6s/64s	Acetone	[63]
α-Fe_2O_3/SnO_2	Heterostructure NTs	90	300	33.4/100 ppm	1 ppm	4.9–5.9 s/15.8–22.5s	Acetone	[188]
SnO_2/In_2O_3	Hetrojunction NTs	80 – 120	300	118/50 ppm	250 ppb	60s/97s	Formaldehyde	[60]
SnO_2/In_2O_3	Hetero HNFs (nano needles on NTs)	450 – 500	290	13.85/10 ppm	0.5 ppm	20s/40s	Formaldehyde	[61]
SnO_2/In_2O_3	hetero-NFs	100 – 250	375	7.5/10 ppm	0.5 ppm	-	Formaldehyde	[189]
In_2O_3–Fe_2O_3	Composite NTs	130	260	15/100 ppm	1 ppm	4s/7 s	Formaldehyde	[187]
In_2O_3 – SnO_2	Hetrojunction NRs	230 – 470	RT	8.98/100 ppm	0.1 ppm	4.67 s/-	NO_x	[19]
ZnO–SnO_2	Heterostructure (NF–NWstem-branch)	120	350	35/0.1 ppm	0.1 ppm	-	NO_2	[190]
SnO_2–ZnO	Composite NFs	55 – 80	200	105/4 ppm	400 ppb	-	NO_2	[191]
In_2O_3 Beads@TiO_2−In_2O_3	Beads on composite NFs	150 – 250	RT	95/97 ppm	0.3 ppm	4.3s/-	NO_2	[45]
In_2O_3/TiO_2	Mesoporous NFs	200	RT	41.1%/97 ppm	97 ppb	3s/-	NO_x	[20]
Al_2O_3–In_2O_3	Mesoporous composite NFs	200	RT	100/97 ppm	291 ppb	28s/-	NO_x	[18]
SnO_2–ZnO	HNFs	80 – 160	190	15.6/50 ppm	1 ppm	6-11s/12-23s	Toluene	[192]
NiO–SnO_2	Composite NFs	80 – 160	330	11/50 ppm	50 ppm	1.2 s/ 4 s	Toluene	[60, 193]
ZnO–SnO_2	Composite NFs	100 – 200	360	9.8/100 ppm	10 ppm	5s/6s	Toluene	[183, 194]

**Material**	**Structure**	**Diameter (nm)**	**Operating Temperature (°C)**	**Response**	**Detection limit**	**Response/recovery time**	**Analyte gas**	**Reference**
In_2O_3–SnO_2	Composite NFs	40 – 100	80	8.1/1 ppm	50 ppb	1s/6s	Trimethyl amine	[2]
NiO/ZnO	Heterojunction NFs	100	260	892/100 ppm	0.5 ppm	30s/35s	trimethylamine	[195]
In_2O_3–SnO_2	Composite NFs	60 – 100	280	~29/100 ppm	2 ppm	8s/15s	Methanol	[196]
SnO_2-ZnO	Heterostructure HNFs	160 – 380	350	~9.0/10 ppm	1 ppm	20s/40s	Methanol	[197]
CuO/SnO_2	Composite NFs	~300	200	23/1 ppm	0.01 ppm	23s/25s	H_2S	[198]
ZnO–CuO	Composite NFs	150 – 200	150	4489.9/10 ppm	1 ppm	-	H_2S	[199]
CuO/SnO_2	Mixed NFs	110	300	522/10 ppm	10 ppm	1s/305s	H_2S	[42]
SnO_2–CuO	Heterojunctions NFs	130 – 200	235	95/10 ppm	-	37s/80s	CO	[200]
SnO_2 – C	Hierarchical heterostructured NFs (nanosheets on NFs)	150 – 500	200	~16.5/100 ppm	1 ppm	4s/16s	H_2	[70]
ZnO/$CoNiO_2$	Composite HNFs	60 – 80	220	240/100 ppm	-	8s/11s	NH_3	[201]

NRs: Nanorods, NBs: Nanobelts

References

[1] Liang, X.; Kim, T.-H.; Yoon, J.-W.; Kwak, C.-H.; Lee, J.-H., *Sensors and Actuators B: Chemical* **2015,** *209*, 934-942.

[2] Qi, Q.; Zou, Y.-C.; Fan, M.-H.; Liu, Y.-P.; Gao, S.; Wang, P.-P.; He, Y.; Wang, D.-J.; Li, G.-D., *Sensors and Actuators B: Chemical* **2014,** *203*, 111-117.

[3] Giancaterini, L.; Emamjomeh, S. M.; De Marcellis, A.; Palange, E.; Resmini, A.; Anselmi-Tamburini, U.; Cantalini, C., *Sensors and Actuators B: Chemical* **2016,** *229*, 387-395.

[4] Senthil, T.; Anandhan, S., *Journal of colloid and interface science* **2014,** *432*, 285-296.

[5] Qi, Q.; Zhao, J.; Xuan, R.-F.; Wang, P.-P.; Feng, L.-L.; Zhou, L.-J.; Wang, D.-J.; Li, G.-D., *Sensors and Actuators B: Chemical* **2014,** *191*, 659-665.

[6] Jang, B.-H.; Landau, O.; Choi, S.-J.; Shin, J.; Rothschild, A.; Kim, I.-D., *Sensors and Actuators B: Chemical* **2013,** *188*, 156-168.

[7] Feng, Q.; Li, X.; Wang, J.; Gaskov, A. M., *Sensors and Actuators B: Chemical* **2016,** *222*, 864-870.

[8] Lim, S. K.; Hwang, S.-H.; Chang, D.; Kim, S., *Sensors and Actuators B: Chemical* **2010,** *149*, 28-33.

[9] Sowti Khiabani, P.; Hosseinmardi, A.; Marzbanrad, E.; Ghashghaie, S.; Zamani, C.; Keyanpour-Rad, M.; Raissi, B., *Sensors and Actuators B: Chemical* **2012,** *162*, 102-107.

[10] Landau, O.; Rothschild, A., *Sensors and Actuators B: Chemical* **2012,** *171–172*, 118-126.

[11] Zhao, M.; Wang, X.; Cheng, J.; Zhang, L.; Jia, J.; Li, X., *Current Applied Physics* **2013,** *13*, 403-407.

[12] Zhao, C.; Huang, B.; Xie, E.; Zhou, J.; Zhang, Z., *Sensors and Actuators B: Chemical* **2015,** *207, Part A*, 313-320.

[13] Zhang, H.-D.; Tang, C.-C.; Long, Y.-Z.; Zhang, J.-C.; Huang, R.; Li, J.-J.; Gu, C.-Z., *Sensors and Actuators A: Physical* **2014,** *219*, 123-127.

[14] Ab Kadir, R.; Li, Z.; Sadek, A. Z.; Abdul Rani, R.; Zoolfakar, A. S.; Field, M. R.; Ou, J. Z.; Chrimes, A. F.; Kalantar-zadeh, K., *The Journal of Physical Chemistry C* **2014,** *118*, 3129-3139.

[15] Zhang, Y.; He, X.; Li, J.; Miao, Z.; Huang, F., *Sensors and Actuators B: Chemical* **2008,** *132*, 67-73.

[16] Wan, G. X.; Ma, S. Y.; Sun, X. W.; Sun, A. M.; Li, X. B.; Luo, J.; Li, W. Q.; Wang, C. Y., *Materials Letters* **2015,** *145*, 48-51.

[17] Xu, L.; Song, H.; Dong, B.; Wang, Y.; Chen, J.; Bai, X., *Inorg. Chem.* **2010,** *49*, 10590-10597.

[18] Gao, J.; Wang, L.; Kan, K.; Xu, S.; Jing, L.; Liu, S.; Shen, P.; Li, L.; Shi, K., *Journal of Materials Chemistry A* **2014,** *2*, 949-956.

[19] Xu, S.; Gao, J.; Wang, L.; Kan, K.; Xie, Y.; Shen, P.; Li, L.; Shi, K., *Nanoscale* **2015,** *7*, 14643-14651.

[20] Wu, H.; Kan, K.; Wang, L.; Zhang, G.; Yang, Y.; Li, H.; Jing, L.; Shen, P.; Li, L.; Shi, K., *CrystEngComm* **2014,** *16*, 9116-9124.

[21] Liu, L.; Li, S.; Guo, X.; Wang, L.; Liu, L.; Wang, X., *Journal of Materials Science: Materials in Electronics* **2016,** *27*, 5153-5157.

[22] Su, C.; Li, Y.; He, Y.; Liu, L.; Wang, X.; Liu, L., *Materials Science in Semiconductor Processing* **2015,** *39*, 49-53.

[23] Wu, J.; Huang, Q.; Zeng, D.; Zhang, S.; Yang, L.; Xia, D.; Xiong, Z.; Xie, C., *Sensors and Actuators B: Chemical* **2014,** *198*, 62-69.

[24] Zheng, W.; Lu, X.; Wang, W.; Li, Z.; Zhang, H.; Wang, Z.; Xu, X.; Li, S.; Wang, C., *Journal of Colloid and Interface Science* **2009,** *338*, 366-370.

[25] Xu, X.; Fan, H.; Liu, Y.; Wang, L.; Zhang, T., *Sensors and Actuators B: Chemical* **2011,** *160*, 713-719.

[26] Wang, X.; Zhao, M.; Liu, F.; Jia, J.; Li, X.; Cao, L., *Ceramics International* **2013,** *39*, 2883-2887.

[27] Koo, W.-T.; Choi, S.-J.; Kim, N.-H.; Jang, J.-S.; Kim, I.-D., *Sensors and Actuators B: Chemical* **2016,** *223*, 301-310.

[28] Bian, H.; Ma, S.; Sun, A.; Xu, X.; Yang, G.; Gao, J.; Zhang, Z.; Zhu, H., *Superlattices and Microstructures* **2015,** *81*, 107-113.

[29] Wang, C.; Ma, S.; Sun, A.; Qin, R.; Yang, F.; Li, X.; Li, F.; Yang, X., *Sensors and Actuators B: Chemical* **2014,** *193*, 326-333.

[30] Song, X.; Liu, L., *Sensors and Actuators A: Physical* **2009,** *154*, 175-179.

[31] Han, L.; Andrady, A. L.; Ensor, D. S., *Sensors and Actuators B: Chemical* **2013,** *186*, 52-55.

[32] Lou, Z.; Deng, J.; Wang, L.; Wang, R.; Fei, T.; Zhang, T., *RSC Advances* **2013,** *3*, 3131-3136.

[33] Wei, S.; Yu, Y.; Zhou, M., *Materials Letters* **2010,** *64*, 2284-2286.

[34] Choi, S. J.; Chattopadhyay, S.; Kim, J. J.; Kim, S. J.; Tuller, H. L.; Rutledge, G. C.; Kim, I. D., *Nanoscale* **2016,** *8*, 9159-9166.

[35] Liu, C.; Chi, X.; Liu, X.; Wang, S., *Journal of Alloys and Compounds* **2014,** *616*, 208-212.

[36] Katoch, A.; Sun, G.-J.; Choi, S.-W.; Byun, J.-H.; Kim, S. S., *Sensors and Actuators B: Chemical* **2013,** *185*, 411-416.

[37] Bai, H.; Zhao, L.; Lu, C.; Li, C.; Shi, G., *Polymer* **2009,** *50*, 3292-3301.

[38] Low, K.; Horner, C. B.; Li, C.; Ico, G.; Bosze, W.; Myung, N. V.; Nam, J., *Sensors and Actuators B: Chemical* **2015,** *207, Part A*, 235-242.

[39] Lu, Q.; Wang, C.; Liu, S.; Ren, M., *Materials Transactions* **2011,** *52*, 1206-1210.

[40] Cao, J.; Dou, H.; Zhang, H.; Mei, H.; Liu, S.; Fei, T.; Wang, R.; Wang, L.; Zhang, T., *Sensors and Actuators B: Chemical* **2014,** *198*, 180-187.

[41] Wang, W.; Li, Z.; Zheng, W.; Huang, H.; Wang, C.; Sun, J., *Sensors and Actuators B: Chemical* **2010,** *143*, 754-758.

[42] Katoch, A.; Kim, J. H.; Kim, S. S., *Journal of Nanoscience and Nanotechnology* **2015,** *15*, 8637-8641.

[43] Yoon, J.-W.; Choi, J.-K.; Lee, J.-H., *Sensors and Actuators B: Chemical* **2012,** *161*, 570-577.

[44] Choi, J.-K.; Hwang, I.-S.; Kim, S.-J.; Park, J.-S.; Park, S.-S.; Jeong, U.; Kang, Y. C.; Lee, J.-H., *Sensors and Actuators B: Chemical* **2010,** *150*, 191-199.

[45] Wang, L.; Gao, J.; Wu, B.; Kan, K.; Xu, S.; Xie, Y.; Li, L.; Shi, K., *ACS Applied Materials and Interfaces* **2015,** *7*, 27152-27159.

[46] Patil, P. T.; Anwane, R. S.; Kondawar, S. B., *Procedia Materials Science* **2015,** *10*, 195-204.

[47] Wei, S.; Wang, S.; Zhang, Y.; Zhou, M., *Sensors and Actuators B: Chemical* **2014,** *192*, 480-487.

[48] Huang, B.; Zhao, C.; Zhang, M.; Zhang, Z.; Xie, E.; Zhou, J.; Han, W., *Applied Surface Science* **2015,** *349*, 615-621.

[49] Pang, Z.; Nie, Q.; Wei, A.; Yang, J.; Huang, F.; Wei, Q., *Journal of Materials Science* **2017,** *52*, 686-695.

[50] Mao, Y.; Ma, S.; Li, X.; Wang, C.; Li, F.; Yang, X.; Zhu, J.; Ma, L., *Applied Surface Science* **2014,** *298*, 109-115.

[51] Xiong, Y.; Xue, Q.; Ling, C.; Lu, W.; Ding, D.; Zhu, L.; Li, X., *Sensors and Actuators, B: Chemical* **2017,** *241*, 725-734.

[52] Cao, Y.; Zou, X.; Wang, X.; Qian, J.; Bai, N.; Li, G.-D., *Sensors and Actuators B: Chemical* **2016,** *232*, 564-570.

[53] Xu, X.; Sun, J.; Zhang, H.; Wang, Z.; Dong, B.; Jiang, T.; Wang, W.; Li, Z.; Wang, C., *Sensors and Actuators B: Chemical* **2011,** *160*, 858-863.

[54] Zhao, C.; Hu, W.; Zhang, Z.; Zhou, J.; Pan, X.; Xie, E., *Sensors and Actuators B: Chemical* **2014,** *195*, 486-493.

[55] Pinto, N. J.; Ramos, I.; Rojas, R.; Wang, P.-C.; Johnson Jr, A. T., *Sensors and Actuators B: Chemical* **2008,** *129*, 621-627.

[56] Pinto, N. J.; Rivera, D.; Melendez, A.; Ramos, I.; Lim, J. H.; Johnson, A. T. C., *Sensors and Actuators B: Chemical* **2011,** *156*, 849-853.

[57] Xu, L.; Dong, B.; Wang, Y.; Bai, X.; Liu, Q.; Song, H., *Sensors and Actuators B: Chemical* **2010,** *147*, 531-538.

[58] Chi, X.; Liu, C.; Liu, L.; Li, S.; Li, H.; Zhang, X.; Bo, X.; Shan, H., *Materials Science in Semiconductor Processing* **2014,** *18*, 160-164.

[59] Lee, C.-S.; Kim, I.-D.; Lee, J.-H., *Sensors and Actuators B: Chemical* **2013,** *181*, 463-470.

[60] Liu, J.; Li, X.; Chen, X.; Niu, H.; Han, X.; Zhang, T.; Lin, H.; Qu, F., *New Journal of Chemistry* **2016,** *40*, 1756-1764.

[61] Du, H.; Wang, J.; Sun, Y.; Yao, P.; Li, X.; Yu, N., *Sensors and Actuators B: Chemical* **2015,** *206*, 753-763.

[62] Deng, J.; Yu, B.; Lou, Z.; Wang, L.; Wang, R.; Zhang, T., *Sensors and Actuators B: Chemical* **2013,** *184*, 21-26.

[63] Feng, C.; Li, X.; Ma, J.; Sun, Y.; Wang, C.; Sun, P.; Zheng, J.; Lu, G., *Sensors and Actuators B: Chemical* **2015,** *209*, 622-629.

[64] Qi, Q.; Wang, P.-P.; Zhao, J.; Feng, L.-L.; Zhou, L.-J.; Xuan, R.-F.; Liu, Y.-P.; Li, G.-D., *Sensors and Actuators B: Chemical* **2014,** *194*, 440-446.

[65] Ji, S.; Li, Y.; Yang, M., *Sensors and Actuators B: Chemical* **2008,** *133*, 644-649.

[66] Wang, Q.; Dong, X.; Pang, Z.; Du, Y.; Xia, X.; Wei, Q.; Huang, F., *Sensors (Switzerland)* **2012,** *12*, 17046-17057.

[67] Li, Y.; Gong, J.; He, G.; Deng, Y., *Materials Chemistry and Physics* **2011,** *129*, 477-482.

[68] Wang, Y.; Jia, W.; Strout, T.; Ding, Y.; Lei, Y., *Sensors* **2009,** *9*, 6752-6763.

[69] Li, Y.; Ban, H.; Yang, M., *Sensors and Actuators B: Chemical* **2016,** *224*, 449-457.

[70] Wang, Z.; Liu, S.; Jiang, T.; Xu, X.; Zhang, J.; An, C.; Wang, C., *RSC Advances* **2015,** *5*, 64582-64587.

[71] Yan, S.; Wu, Q., *Sensors and Actuators B: Chemical* **2014,** *205*, 329-337.

[72] Abideen, Z. U.; Katoch, A.; Kim, J.-H.; Kwon, Y. J.; Kim, H. W.; Kim, S. S., *Sensors and Actuators B: Chemical* **2015,** *221*, 1499-1507.

[73] Abideen, Z. U.; Park, J. Y.; Kim, H. W.; Kim, S. S., *Nanotechnology* **2017,** *28*, 035501.

[74] Kim, J. H.; Zheng, Y.; Mirzaei, A.; Kim, H. W.; Kim, S. S., *Journal of Electronic Materials* **2017**, 1-11.

[75] Guo, J.; Liu, X.; Wang, H.; Sun, W.; Sun, J., *Materials Letters* **2017,** *209*, 102-105.

[76] Liu, S.; Sun, H.; Nagarajan, R.; Kumar, J.; Gu, Z.; Cho, J.; Kurup, P., *Sensors and Actuators A: Physical* **2011,** *167*, 8-13.

[77] Lin, Q.; Li, Y.; Yang, M., *Analytica Chimica Acta* **2012,** *748*, 73-80.

[78] Liu, Y.; Huang, H.; Wang, L.; Cai, D.; Liu, B.; Wang, D.; Li, Q.; Wang, T., *Sensors and Actuators B: Chemical* **2016,** *223*, 730-737.

[79] He, X.; Arsat, R.; Sadek, A. Z.; Wlodarski, W.; Kalantar-zadeh, K.; Li, J., *Sensors and Actuators B: Chemical* **2010,** *145*, 674-679.

[80] Sheng, L.; Dajing, C.; Yuquan, C., *Nanotechnology* **2011,** *22*, 265504.

[81] Aria, M. M.; Irajizad, A.; Astaraei, F. R.; Shariatpanahi, S. P.; Sarvari, R., *Measurement* **2016,** *78*, 283-288.

[82] Zhang, C.; Wang, X.; Lin, J.; Ding, B.; Yu, J.; Pan, N., *Sensors and Actuators B: Chemical* **2011,** *152*, 316-323.

[83] Ding, B.; Wang, X.; Yu, J.; Wang, M., *Journal of Materials Chemistry* **2011,** *21*, 12784-12792.

[84] Ding, B.; Yamazaki, M.; Shiratori, S., *Sensors and Actuators B: Chemical* **2005,** *106*, 477-483.

[85] Jia, Y.; Yan, C.; Yu, H.; Chen, L.; Dong, F., *Sensors and Actuators B: Chemical* **2014,** *203*, 459-464.

[86] Jia, Y.; Yu, H.; Zhang, Y.; Chen, L.; Dong, F., *Sensors and Actuators B: Chemical* **2015,** *212*, 273-277.

[87] Ding, B.; Kim, J.; Miyazaki, Y.; Shiratori, S., *Sensors and Actuators B: Chemical* **2004,** *101*, 373-380.

[88] Wang, X.; Cui, F.; Lin, J.; Ding, B.; Yu, J.; Al-Deyab, S. S., *Sensors and Actuators B: Chemical* **2012,** *171–172*, 658-665.

[89] Jia, Y.; Chen, L.; Yu, H.; Zhang, Y.; Dong, F., *RSC Advances* **2015,** *5*, 40620-40627.

[90] Viter, R.; Chaaya, A. A.; Iatsunskyi, I.; Nowaczyk, G.; Kovalevskis, K.; Erts, D.; Miele, P.; Smyntyna, V.; Bechelany, M., *Nanotechnology* **2015,** *26*, 105501.

[91] Luoh, R.; Hahn, H. T., *Composites Science and Technology* **2006,** *66*, 2436-2441.

[92] Long, Y.; Chen, H.; Wang, H.; Peng, Z.; Yang, Y.; Zhang, G.; Li, N.; Liu, F.; Pei, J., *Analytica chimica acta* **2012,** *744*, 82-91.

[93] Long, Y.; Chen, H.; Yang, Y.; Wang, H.; Yang, Y.; Li, N.; Li, K.; Pei, J.; Liu, F., *Macromolecules* **2009,** *42*, 6501-6509.

[94] Urrutia, A.; Goicoechea, J.; Rivero, P. J.; Matías, I. R.; Arregui, F. J., *Sensors and Actuators B: Chemical* **2013,** *176*, 569-576.

[95] Xue, R.; Behera, P.; Xu, J.; Viapiano, M. S.; Lannutti, J. J., *Sensors and Actuators B: Chemical* **2014,** *192*, 697-707.

[96] Wolf, C.; Tscherner, M.; Köstler, S., *Sensors and Actuators B: Chemical* **2015,** *209*, 1064-1069.

[97] Wolf, C.; Tscherner, M.; Köstler, S.; Ribitsch, V. Optochemical sensors based on polymer nanofibers with ultra-fast response characteristics. In *Proceedings of SENSORS, 2014 IEEE*, 2014; 950-953.

[98] Lv, Y.-Y.; Wu, J.; Xu, Z.-K., *Sensors and Actuators B: Chemical* **2010,** *148*, 233-239.

[99] Park, J.-A.; Moon, J.; Lee, S.-J.; Kim, S. H.; Zyung, T.; Chu, H. Y., *Materials Letters* **2010,** *64*, 255-257.

[100] Park, J. A.; Moon, J.; Lee, S. J.; Kim, S. H.; Zyung, T.; Chu, H. Y., *Thin Solid Films* **2010,** *518*, 6642-6645.

[101] Zhang, J.; Choi, S.-W.; Kim, S. S., *Journal of Solid State Chemistry* **2011,** *184*, 3008-3013.

[102] Kim, I.-D.; Jeon, E.-K.; Choi, S.-H.; Choi, D.-K.; Tuller, H. L., *Journal of electroceramics* **2010,** *25*, 159-167.

[103] Park, J.-A.; Moon, J.; Lee, S.-J.; Lim, S.-C.; Zyung, T., *Current Applied Physics* **2009,** *9*, S210-S212.

[104] Wei, S.; Zhao, G.; Du, W.; Tian, Q., *Vacuum* **2016,** *124*, 32-39.

[105] Chi, X.; Liu, C.; Liu, L.; Li, Y.; Wang, Z.; Bo, X.; Liu, L.; Su, C., *Sensors and Actuators B: Chemical* **2014,** *194*, 33-37.

[106] Li, W. Q.; Ma, S. Y.; Luo, J.; Mao, Y. Z.; Cheng, L.; Gengzang, D. J.; Xu, X. L.; Yan, S. H., *Materials Letters* **2014,** *132*, 338-341.

[107] Wei, S.; Zhou, M.; Du, W., *Sensors and Actuators B: Chemical* **2011,** *160*, 753-759.

[108] Yu, X.; Song, F.; Zhai, B.; Zheng, C.; Wang, Y., *Physica E: Low-dimensional Systems and Nanostructures* **2013,** *52*, 92-96.

[109] Liang, X.; Jin, G.; Liu, F.; Zhang, X.; An, S.; Ma, J.; Lu, G., *Ceramics International* **2015,** *41*, 13780-13787.

[110] KARIMI, E. Z.; ESMAEILZADEH, J.; MARZBANRAD, E., *Bulletin of Materials Science* **2015,** *38*, 209-214.

[111] Giancaterini, L.; Emamjomeh, S. M.; De Marcellis, A.; Palange, E.; Cantalini, C., *Procedia Engineering* **2015,** *120*, 791-794.

[112] Cho, N. G.; Yang, D. J.; Jin, M.-J.; Kim, H.-G.; Tuller, H. L.; Kim, I.-D., *Sensors and Actuators B: Chemical* **2011,** *160*, 1468-1472.

[113] Zheng, W.; Li, Z.; Zhang, H.; Wang, W.; Wang, Y.; Wang, C., *Materials Research Bulletin* **2009,** *44*, 1432-1436.

[114] Cheng, L.; Ma, S. Y.; Wang, T. T.; Li, X. B.; Luo, J.; Li, W. Q.; Mao, Y. Z.; Gz, D. J., *Materials Letters* **2014,** *131*, 23-26.

[115] Zheng, W.; Lu, X.; Wang, W.; Li, Z.; Zhang, H.; Wang, Y.; Wang, Z.; Wang, C., *Sensors and Actuators B: Chemical* **2009,** *142*, 61-65.

[116] Liu, Y.; Gao, C.; Pan, X.; An, X.; Xie, Y.; Zhou, M.; Song, J.; Zhang, H.; Liu, Z.; Zhao, Q.; Zhang, Y.; Xie, E., *Applied Surface Science* **2011,** *257*, 2264-2268.

[117] Katoch, A.; Choi, S.-W.; Kim, H. W.; Kim, S. S., *Journal of Hazardous Materials* **2015,** *286*, 229-235.

[118] Cui, J.; Shi, L.; Xie, T.; Wang, D.; Lin, Y., *Sensors and Actuators B: Chemical* **2016,** *227*, 220-226.

[119] Li, Z.; Fan, Y.; Zhan, J., *European Journal of Inorganic Chemistry* **2010**, 3348-3353.

[120] Chi, X.; Liu, C.; Zhang, J.; Liu, L.; Li, H.; He, Y.; Bo, X.; Liu, L., *Journal of Semiconductors* **2014,** *35*, 064005.

[121] Cheng, L.; Ma, S. Y.; Li, X. B.; Luo, J.; Li, W. Q.; Li, F. M.; Mao, Y. Z.; Wang, T. T.; Li, Y. F., *Sensors and Actuators B: Chemical* **2014,** *200*, 181-190.

[122] Cheng, J. P.; Wang, B. B.; Zhao, M. G.; Liu, F.; Zhang, X. B., *Sensors and Actuators B: Chemical* **2014,** *190*, 78-85.

[123] Tang, W.; Wang, J.; Qiao, Q.; Liu, Z.; Li, X., *Journal of Materials Science* **2015,** *50*, 2605-2615.

[124] Xu, X.; Chen, Y.; Zhang, G.; Ma, S.; Lu, Y.; Bian, H.; Chen, Q., *Journal of Alloys and Compounds* **2017,** *703*, 572-579.

[125] Shan, H.; Liu, C.; Liu, L.; Li, S.; Wang, L.; Zhang, X.; Bo, X.; Chi, X., *Sensors and Actuators B: Chemical* **2013,** *184*, 243-247.

[126] Su, C.; Zou, Y.; Xu, X.; Liu, L.; Liu, Z.; Liu, L., *Colloids and Surfaces A: Physicochemical and Engineering Aspects* **2015,** *472*, 63-68.

[127] Su, C.; Liu, C.; Liu, L.; Ni, M.; Li, H.; Bo, X.; Liu, L.; Chi, X., *Applied Surface Science* **2014,** *314*, 931-935.

[128] Liu, C.; Shan, H.; Liu, L.; Li, S.; Li, H., *Ceramics International* **2014,** *40*, 2395-2399.

[129] Zhao, C.; Bai, J.; Huang, B.; Wang, Y.; Zhou, J.; Xie, E., *Sensors and Actuators B: Chemical* **2016,** *231*, 552-560.

[130] Wan, G. X.; Ma, S. Y.; Li, X. B.; Li, F. M.; Bian, H. Q.; Zhang, L. P.; Li, W. Q., *Materials Letters* **2014,** *114*, 103-106.

[131] Li, W.; Ma, S.; Yang, G.; Mao, Y.; Luo, J.; Cheng, L.; Gengzang, D.; Xu, X.; Yan, S., *Materials Letters* **2015,** *138*, 188-191.

[132] Sun, Y.; Zhao, Z.; Li, P.; Li, G.; Chen, Y.; Zhang, W.; Hu, J., *Applied Surface Science* **2015,** *356*, 73-80.

[133] Liu, L.; Li, S.; Zhuang, J.; Wang, L.; Zhang, J.; Li, H.; Liu, Z.; Han, Y.; Jiang, X.; Zhang, P., *Sensors and Actuators B: Chemical* **2011,** *155*, 782-788.

[134] Xu, X. L.; Chen, Y.; Ma, S. Y.; Li, W. Q.; Mao, Y. Z., *Sensors and Actuators B: Chemical* **2015,** *213*, 222-233.

[135] Feng, C.; Wang, C.; Cheng, P.; Li, X.; Wang, B.; Guan, Y.; Ma, J.; Zhang, H.; Sun, Y.; Sun, P.; Zheng, J.; Lu, G., *Sensors and Actuators B: Chemical* **2015,** *221*, 434-442.

[136] Bai, X.; Ji, H.; Gao, P.; Zhang, Y.; Sun, X., *Sensors and Actuators B: Chemical* **2014,** *193*, 100-106.

[137] Choi, S.-J.; Lee, I.; Jang, B.-H.; Youn, D.-Y.; Ryu, W.-H.; Park, C. O.; Kim, I.-D., *Analytical chemistry* **2013,** *85*, 1792-1796.

[138] Lian, H.; Wang, G.; Yue, H.; Liu, L.; Guo, X.; Wang, X., *Micro and Nano Letters* **2016,** *11*, 825-827.

[139] Liu, L.; Zhang, T.; Wang, L.; Li, S., *Materials Letters* **2009,** *63*, 2041-2043.

[140] Li, W. Q.; Ma, S. Y.; Li, Y. F.; Li, X. B.; Wang, C. Y.; Yang, X. H.; Cheng, L.; Mao, Y. Z.; Luo, J.; Gengzang, D. J.; Wan, G. X.; Xu, X. L., *Journal of Alloys and Compounds* **2014,** *605*, 80-88.

[141] Guo, L.; Xie, N.; Wang, C.; Kou, X.; Ding, M.; Zhang, H.; Sun, Y.; Song, H.; Wang, Y.; Lu, G., *Sensors and Actuators B: Chemical* **2018,** *255*, 1015-1023.

[142] Jiang, Z.; Jiang, T.; Wang, J.; Wang, Z.; Xu, X.; Wang, Z.; Zhao, R.; Li, Z.; Wang, C., *Journal of Colloid and Interface Science* **2015,** *437*, 252-258.

[143] Wang, T. T.; Ma, S. Y.; Cheng, L.; Luo, J.; Jiang, X. H.; Jin, W. X., *Sensors and Actuators B: Chemical* **2015,** *216*, 212-220.

[144] Mohanapriya, P.; Segawa, H.; Watanabe, K.; Watanabe, K.; Samitsu, S.; Natarajan, T. S.; Jaya, N. V.; Ohashi, N., *Sensors and Actuators B: Chemical* **2013,** *188*, 872-878.

[145] Wang, Z.; Liu, L., *Materials Letters* **2009,** *63*, 917-919.

[146] Kou, X.; Wang, C.; Ding, M.; Feng, C.; Li, X.; Ma, J.; Zhang, H.; Sun, Y.; Lu, G., *Sensors and Actuators, B: Chemical* **2016,** *236*, 425-432.

[147] Li, Z.; Dzenis, Y., *Talanta* **2011,** *85*, 82-85.

[148] Liu, L.; Zhang, T.; Li, S.; Wang, L.; Tian, Y., *Materials Letters* **2009,** *63*, 1975-1977.

[149] Liu, L.; Guo, C.; Li, S.; Wang, L.; Dong, Q.; Li, W., *Sensors and Actuators B: Chemical* **2010,** *150*, 806-810.

[150] Dong, K.-Y.; Choi, J.-K.; Hwang, I.-S.; Lee, J.-W.; Kang, B. H.; Ham, D.-J.; Lee, J.-H.; Ju, B.-K., *Sensors and Actuators B: Chemical* **2011,** *157*, 154-161.

[151] Lian, H.; Feng, Y.; Wang, Z.; Liu, L.; Guo, X.; Wang, X., *Applied Physics A: Materials Science and Processing* **2017,** *123*, 123:158.

[152] Chen, D. D.; Li, Z.; Jin, X.; Yi, J. X., *Chin. J. Chem. Phys.* **2017,** *30*, 474-478.

[153] Cho, N. G.; Woo, H.-S.; Lee, J.-H.; Kim, I.-D., *Chemical Communications* **2011,** *47*, 11300-11302.

[154] Ma, L.; Ma, S. Y.; Kang, H.; Shen, X. F.; Wang, T. T.; Jiang, X. H.; Chen, Q., *Materials Letters* **2017,** *209*, 188-192.

[155] Zhao, M.; Wang, X.; Ning, L.; Jia, J.; Li, X.; Cao, L., *Sensors and Actuators B: Chemical* **2011,** *156*, 588-592.

[156] Liu, J.; Guo, W.; Qu, F.; Feng, C.; Li, C.; Zhu, L.; Zhou, J.; Ruan, S.; Chen, W., *Ceramics International* **2014,** *40*, 6685-6689.

[157] Yang, J.; Gao, C.; Yang, H.; Wang, X.; Jia, J., *Eur. Phys. J. Appl. Phys.* **2017,** *79*, 30101.

[158] Kim, N.-H.; Choi, S.-J.; Yang, D.-J.; Bae, J.; Park, J.; Kim, I.-D., *Sensors and Actuators B: Chemical* **2014,** *193*, 574-581.

[159] Ma, S.; Jia, J.; Tian, Y.; Cao, L.; Shi, S.; Li, X.; Wang, X., *Ceramics International* **2016,** *42*, 2041-2044.

[160] Lin, Y.; Wei, W.; Li, Y.; Li, F.; Zhou, J.; Sun, D.; Chen, Y.; Ruan, S., *Journal of Alloys and Compounds* **2015,** *651*, 690-698.

[161] Wang, X.; Zhang, J.; Wang, L.; Li, S.; Liu, L.; Su, C.; Liu, L., *Journal of Materials Science & Technology* **2015,** *31*, 1175-1180.

[162] Wang, X.; Li, H.; Ni, M.; Wang, L.; Liu, L.; Wang, H.; Guo, X., *Journal of Electronic Materials* **2017,** *46*, 363-369.

[163] Wang, X.; Zhang, J.; He, Y.; Wang, L.; Liu, L.; Wang, H.; Guo, X.; Lian, H., *Chemical Physics Letters* **2016,** *658*, 319-323.

[164] Liu, C.; Wang, X.; Xie, F.; Liu, L.; Ruan, S., *Journal of Materials Science: Materials in Electronics* **2016,** *27*, 9870-9876.

[165] Wang, J.; Zou, B.; Ruan, S.; Zhao, J.; Chen, Q.; Wu, F., *Materials Letters* **2009,** *63*, 1750-1753.

[166] He, Y.; Wang, D.; Ge, F.; Liu, L., *Journal of Semiconductors* **2015,** *36*, 083005.

[167] Feng, C.; Li, X.; Wang, C.; Sun, Y.; Zheng, J.; Lu, G., *RSC Advances* **2014,** *4*, 47549-47555.

[168] Kim, J. H.; Zheng, Y.; Mirzaei, A.; Kim, S. S., *Korean Journal of Materials Research* **2016,** *26*, 741-750.

[169] Katoch, A.; Byun, J.-H.; Choi, S.-W.; Kim, S. S., *Sensors and Actuators B: Chemical* **2014,** *202*, 38-45.

[170] Feng, C.; Wang, C.; Zhang, H.; Li, X.; Wang, C.; Cheng, P.; Ma, J.; Sun, P.; Gao, Y.; Zhang, H.; Sun, Y.; Zheng, J.; Lu, G., *Sensors and Actuators B: Chemical* **2015,** *221*, 1475-1482.

[171] Moon, J.; Park, J.-A.; Lee, S.-J.; Zyung, T.; Kim, I.-D., *Sensors and Actuators B: Chemical* **2010,** *149*, 301-305.

[172] Kim, J. H.; Abideen, Z. U.; Zheng, Y.; Kim, S. S., *Sensors* **2016,** *16*, 1857.

[173] Yang, X.; Salles, V.; Kaneti, Y. V.; Liu, M.; Maillard, M.; Journet, C.; Jiang, X.; Brioude, A., *Sensors and Actuators B: Chemical* **2015,** *220*, 1112-1119.

[174] Wang, Y.; Zhang, H.; Sun, X., *Applied Surface Science* **2016,** *389*, 514-520.

[175] Liu, Y.; Yang, P.; Li, J.; Matras-Postolek, K.; Yue, Y.; Huang, B., *RSC Advances* **2016,** *6*, 13371-13376.

[176] Yan, S. H.; Ma, S. Y.; Li, W. Q.; Xu, X. L.; Cheng, L.; Song, H. S.; Liang, X. Y., *Sens Actuators, B Chem* **2015,** *221*, 88-95.

[177] Liu, Y.; Yang, P.; Li, J.; Matras-Postolek, K.; Yue, Y.; Huang, B., *RSC Advances* **2015,** *5*, 98500-98507.

[178] Li, W.; Ma, S.; Li, Y.; Yang, G.; Mao, Y.; Luo, J.; Gengzang, D.; Xu, X.; Yan, S., *Sens Actuators, B Chem* **2015,** *211*, 392-402.

[179] Yan, S.; Wu, Q., *Sens Actuators, B Chem* **2014,** *205*, 329-337.

[180] Wang, B. B.; Fu, X. X.; Liu, F.; Shi, S. L.; Cheng, J. P.; Zhang, X. B., *Journal of Alloys and Compounds* **2014,** *587*, 82-89.

[181] Khorami, H. A.; Keyanpour-Rad, M.; Vaezi, M. R., *Applied Surface Science* **2011,** *257*, 7988-7992.

[182] Yan, C.; Lu, H.; Gao, J.; Zhu, G.; Yin, F.; Yang, Z.; Liu, Q.; Li, G., *Journal of Alloys and Compounds* **2017,** *699*, 567-574.

[183] Li, F.; Gao, X.; Wang, R.; Zhang, T.; Lu, G., *Sensors and Actuators, B: Chemical* **2016**, 812-819.

[184] Yan, S. H.; Ma, S. Y.; Xu, X. L.; Li, W. Q.; Luo, J.; Jin, W. X.; Wang, T. T.; Jiang, X. H.; Lu, Y.; Song, H. S., *Materials Letters* **2015,** *159*, 447-450.

[185] Li, X.; Zhang, H.; Feng, C.; Sun, Y.; Ma, J.; Wang, C.; Lu, G., *RSC Advances* **2014,** *4*, 27552-27555.

[186] Chi, X.; Liu, C.; Li, Y.; Li, H.; Liu, L.; Bo, X.; Liu, L.; Su, C., *Materials Science in Semiconductor Processing* **2014,** *27*, 494-499.

[187] Liu, L.; Li, S.; Guo, X.; He, Y.; Wang, L., *Journal of Semiconductors* **2016,** *37*, 013005.

[188] Yu, Q.; Zhu, J.; Xu, Z.; Huang, X., *Sensors and Actuators B: Chemical* **2015,** *213*, 27-34.

[189] Du, H.; Wang, J.; Su, M.; Yao, P.; Zheng, Y.; Yu, N., *Sensors and Actuators B: Chemical* **2012,** *166–167*, 746-752.

[190] Choi, S.-W.; Katoch, A.; Sun, G.-J.; Kim, S. S., *Sensors and Actuators B: Chemical* **2013,** *181*, 787-794.

[191] Park, J.-A.; Moon, J.; Lee, S.-J.; Kim, S. H.; Chu, H. Y.; Zyung, T., *Sensors and Actuators B: Chemical* **2010,** *145*, 592-595.

[192] Wei, S.; Zhang, Y.; Zhou, M., *Solid State Communications* **2011,** *151*, 895-899.

[193] Liu, L.; Zhang, Y.; Wang, G.; Li, S.; Wang, L.; Han, Y.; Jiang, X.; Wei, A., *Sensors and Actuators B: Chemical* **2011,** *160*, 448-454.

[194] Song, X.; Zhang, D.; Fan, M., *Applied Surface Science* **2009,** *255*, 7343-7347.

[195] Li, C.; Feng, C.; Qu, F.; Liu, J.; Zhu, L.; Lin, Y.; Wang, Y.; Li, F.; Zhou, J.; Ruan, S., *Sensors and Actuators B: Chemical* **2015,** *207, Part A*, 90-96.

[196] Zheng, W.; Lu, X.; Wang, W.; Dong, B.; Zhang, H.; Wang, Z.; Xu, X.; Wang, C., *Journal of the American Ceramic Society* **2010,** *93*, 15-17.

[197] Tang, W.; Wang, J.; Yao, P.; Li, X., *Sensors and Actuators B: Chemical* **2014,** *192*, 543-549.

[198] Zhao, Y.; He, X.; Li, J.; Gao, X.; Jia, J., *Sensors and Actuators B: Chemical* **2012,** *165*, 82-87.

[199] Katoch, A.; Choi, S.-W.; Kim, J.-H.; Lee, J. H.; Lee, J.-S.; Kim, S. S., *Sensors and Actuators B: Chemical* **2015,** *214*, 111-116.

[200] Bai, S.; Guo, W.; Sun, J.; Li, J.; Tian, Y.; Chen, A.; Luo, R.; Li, D., *Sensors and Actuators B: Chemical* **2016,** *226*, 96-103.

[201] Alali, K. T.; Liu, T.; Liu, J.; Liu, Q.; Fertassi, M. A.; Li, Z.; Wang, J., *Journal of Alloys and Compounds* **2017,** *702*, 20-30.
